# Acknowledgment to Reviewers of *Molecules* in 2021

**DOI:** 10.3390/molecules27041233

**Published:** 2022-02-12

**Authors:** Molecules Editorial Office

**Affiliations:** MDPI AG, St. Alban-Anlage 66, 4052 Basel, Switzerland

Rigorous peer-reviews are the basis of high-quality academic publishing. Thanks to the great efforts of our reviewers, *Molecules* was able to maintain its standards for the high quality of its published papers. Thanks to the contribution of our reviewers, in 2021, the median time to first decision was 14 days, and the median time to publication was 34 days. The editors would like to extend their gratitude and recognition to the following reviewers for their precious time and dedication, regardless of whether the papers they reviewed were finally published:
Aadil, Rana MuahammadLittle, R. DanAbakumov, ArtemLitwin, TomaszAbankwa, DanielLiu, BaiquanAbate, GiuliaLiu, Bing-LanAbbasi, Sumra WajidLiu, ChaoxingAbbasi-Maleki, SaeidLiu, Cheng-HsienAbbaspour-Ravasjani, SoheilLiu, ChengweiAbbate, SergioLiu, Chia-ChiAbbehausen, CamillaLiu, ChunliAbbrent, SabinaLiu, FengAbd El-Aziz, Tarek MohamedLiu, FufengAbd El-Lateef, HanyLiu, GangAbd Razak, Saiful IzwanLiu, HaiAbdallah, Mohamed F.Liu, HaijunAbdelaal, ElsayedLiu, HongbingAbdel-Azeem, Ahmed MohamedLiu, JianningAbd-El-Aziz, AhmadLiu, JianyongAbdelaziz, Omar Y.Liu, JieminAbdelbaky, MohammedLiu, JinlongAbdelfattah, MohamedLiu, Jun-JenAbd-ElGawad, Ahmed M.Liu, KuanAbdelwhab, El-Sayed M.Liu, KunAbdennebi-Najar, LatifaLiu, LishaAboudzadeh, M. AliLiu, PengzhanAbouelenein, DoaaLiu, QingfengAbraham, Wolf-RainerLiu, QuanlinAbrahão Nencioni, Ana LeonorLiu, RuiAbramova, Tatyana V.Liu, ShaofengAbulkhair, Hamada S.Liu, Shiuh-TzungAccioni, FrancescaLiu, TaihongAcedo, Jeella Z.Liu, WenbinAceto, MaurizioLiu, XinAcevedo, Juan JoseLiu, YangAcharya, DhirajLiu, YanhongAchelle, SylvainLiu, YaodongAchour, BrahimLiu, YongfengAćimović, MilicaLiu, YongqiangAci-Sèche, SamiaLiu, YouzhongAcquaviva, RosariaLiu, YuAcunha, TanizeLiu, ZenggenAdamczak, ArturLiu, ZhiAdamczyk, GretaLiu, ZhirongAdamczyk-Woźniak, AgnieszkaLiu, ZileiAdamenko, KingaLiwo, AdamAdams, Brian D.Llata, Marta RuizAdamski, ZbigniewLo Faro, Maria JosèAdamus, GrażynaLobato Tapia, Carlos AlbertoAdebo, OluwafemiLobo-Junior, Eulicio De OliveiraAdewale, OlusolaLobón, Natividad ChavesAdhikari, Gopi ChandraLocascio, AnnamariaAdhikary, AmitavaLogan, IsabelleAdil, MohdLoganina, ValentinaAdil, Syed FarooqLoginov, Dmitry A.Adnan, MdLoglio, GiuseppeAdnan, MohdLoi, MartinaAdriana, PauceanLoiola, Adonay RodriguesAfify, Ahmed SabryLoizzo, Monica R.Afonso, Carlos M.Lojkova, LeaAfrin, SadiaLojou, ElisabethAfsari, MortezaLokesh, Belur R.Agamennone, MariangelaLokstein, HeikoAghazada, SadigLombardi, TommasoAgrawal, DineshLomberget, ThierryAguado, JulioLommel, MarcusAgudelo, AndrésLomonaco, TommasoAguiar, AndréLončar, Mirela BausAguiar, CristianeLondoño, Martha ElenaAguiar, CristinaLongato, Giovanna BarbariniAguilar-Zarate, PedroLongo, AngelaAguirre, Miguel ÁngelLonguespee, RemiAguirre-Álvarez, GabrielLönnberg, HarriAgustín Panadero, RubénLopes, Carla MartinsAgustin, DominiqueLopes, GracilianaAhamad, ShahzaibLopes, SusyAhanger, Mohammad AbassLopez De Las Hazas, Maria-CarmenAhmad, KhurshidLópez Lafuente, AntonioAhmad, Mohammad ZakiLópez Malo, DanielAhmadpoor, FatemehLópez, Jesús AdriánAhmed, QamarLopez, Jose CristobalAhmed, ShabbirLópez, Kelly Johana FigueroaAhmed, TofayelLópez, LluviaAhn, SangdooLópez-Ahumada, Guadalupe A.Ahsan Saeed, MuhammadLopez-Albarran, PabloAhsan, WaquarLópez-Coca, IgnacioAhumada, GuillermoLopez-Escamez, Jose AntonioAiello, FrancescaLópez-Fernández, AurelioAili, SamiraLópez-Guerrero, María del MarAires, AlfredoLópez-Jiménez, ElenaAisa, Haji AkberLópez-Miranda, VisitaciónAit Kaddour, AbderrahmaneLoppinet, BenoitAitipamula, SrinivasuluLordan, RonanAitken, R. AlanLorenc-Koci, ElżbietaAja, AravamudhanLorente, CarolinaAjay, Amrendra K.Lorenz, HeikeAjduković, JovanaLorenzetti, StefanoAjibade, Peter A.Lorenzo, ArnaboldiAjori, ShahramLorenzo, Jose M.Akamatsu, KenLorenzo-Morales, JacobAkashi, HaruoLorimier, SandrineAkçan, RamazanLotina-Hennsen, BlasAkhade, Sneha A.Loureiro, Joana A.Akhavan, OmidLourenço, AnaAkil, SuzannaLourenço, CéliaAkimov, Sergey A.Lovrić, MarioAksenov, SergeyLoza-Mejía, Marco AntonioAkurathi, GopalakrishnaLozano Guerrero, Antonio JoséAl Moustafa, Ala-EddinLožienė, KristinaAl Rugaie, OsamahLozinsky, VladimirAl-Agamy, Mohamed H.Lu, Chia-JungAlagarasu, KalichamyLu, Chih-HaoAlaimo, AlessandroLu, Hiep ThuanAlam, MahboobLu, LehuiAlam, Md BadrulLu, QiongqiongAlam, ParvejLu, ShaoyongAlañon, ElenaLu, TaoAlao, John PatrickLu, XiaoAlarcon, JulioLu, XinyiAlarif, Walied MohamedLu, YangfanAl-Baldawi, Israa Abdul WahabLu, YongboAlbano, GianluigiLu, ZhenweiAlbarracín, Sonia L.Lu, ZhiyingAlberto, VertovaLubin-Germain, NadègeAlbo, JonathanLuca, AndreiAlbulescu, Irina C.Lucchese, AlessandraAlbuquerque, HélioLucena, RafaelAlcarazo, ManuelLucenti, ElenaAlcocer-Gómez, ElísabetLučić, BonoAlcolea Palafox, MauricioLuck, RudyAlcolea, VeronicaLuckham, PaulAlduina, RosaŁuczaj, WojciechAleandri, SimoneLudwiczuk, AgnieszkaAleixandre, ManuelLudwig, IziarAlejandro-Martín, SergueiLuís, ÂngeloAlejo-Armijo, AlfonsoLujanienė, GalinaAleksandar, CingelŁukasiewicz, MarcinAleksić, IvanaLukić, MarinaAleluia, Milena MagalhãesLukšič, MihaAlessenko, AliceLuliński, PiotrAlessio, NicolaLuna Moreno, DonatoAlexa, ErsiliaLundin, LisaAlexander, WilliamLunge, Vagner RicardoAlexiou, ChristophLungu, Claudiu N.Alexis, FrankLunter, DominiqueAl-Fatimi, MohamedLuo, DanmengAlfei, SilvanaLuo, JianquanAlgammal, Abdelazeem M.Luo, Kai-JunAlguacil, FranciscoLuo, QunAl-Horani, Rami A.Luo, TianyiAli, AhmedLuo, WentaiAli, AkhtarLuo, XianAli, GhulamLupiáñez, José A.Ali, Lamiaa M. A.Luque, PrisciAli, Mohd SajidLurie, SusanAli, Moustafa R. K.Lussana, FedericoAli, Syed AzmalLutfy, KabirullahAliaño-González, Maria JoséLuther King, Abia AkebeAlijah, AlexanderLützenkirchen, JohannesAliofkhazraei, MahmoodLuxbacher, ThomasAl-Karmalawy, Ahmed A.Luxen, AndréAl-Khrasani, MahmoudLuzina, OlgaAllahverdiyeva, YagutLuzzio, FredAllais, FlorentŁyczko, JacekAllard-Chamard, HuguesLysova, Anna A.Allende, MiguelŁyszczek, RenataAllison, LizabethLyu, ZhigangAl-Maharik, NawafMa, GuanghuiAlmansa, María SoledadMa, XiaoxiaAlmanza-Pérez, Julio CésarMa, XueyingAlmáši, MiroslavMa, YanpingAlmeida, Adelaide X.Ma, YifanAlmeida, Ana RitaMa, YingqunAlmeida, CatarinaMa, YufeiAlmerico, Anna MariaMa, ZhuoranAl-Mrabeh, AhmadMaccagno, MassimoAlonso, ConcepciónMaccallini, CristinaAlonso-Torre, SaraMaccari, RosannaAlotaibi, MohammadMacDowell, Luis G.Alov, PetkoMacetti, GiovanniAl-Sayah, MohammadMachado, FabricioAlt, HelmutMachado, Michel MansurAltamimi, MohammadMachetti, FabrizioAltosaar, IllimarMachuca, ÁngelaAlvarado-Lassman, AlejandroMachuca-Martínez, FidermanAlvarez Lorenzo, CarmenMaciá, BeatrizAlvarez, AlejandraMaciejewski, HieronimAlvarez, JoséMaciel, CarlosAlves, FranciscoMaciuk, AlexandreAlves, Rita M. B.Maciver, SutherlandAlves, Tiago ViniciusMack, JohnAlves-Silva, Jorge M.MacKay, JohnAlwarthan, AbdulrahmanMaçôas, ErmelindaAlygizakis, NikiforosMadalina, IugaAmadeo, AlidaMadrid, AlejandroAmal, HaithamMadura, IzabelaAmarowicz, RyszardMaeda-Chubachi, TomokoAmero, CarlosMaekawa, MasamitsuAmézqueta, SusanaMaeng, Han-JooAmigo-Benavent, MiryamMaestro, AitorAmin, SamiulMaffei, FrancescaAmmazzalorso, AlessandraMaffia, MicheleAmorim, Cleber A.Mafra, Marcos RogérioAmornlerdpison, DoungpornMagdaleno-Madrigal, Victor M.Amosova, Svetlana V.Maggi, Leonard B.Amovilli, ClaudioMagkoev, Tamerlan T.An, FeifeiMagnaldo, ThierryAn, JieMagnani, AgneseAnas, Andrea Roxanne J.Magoulas, GeorgeAnashkina, ElenaMahat Chhetri, PradhumnaAnastasescu, MihaiMahato, NeelimaAncuceanu, RobertMahdi, ZamaniAndabaka, ŽeljkoMahmood, QaisarAnděra, LadislavMahmoudi-Moghaddam, HadiAndersson, Mats X.Mahmoudvand, HosseinAndicsová Eckstein, AnitaMahmudov, Kamran T.Andonova, VelichkaMahomoodal, FawziAndrade Cetto, AdolfoMahuteau-Betzer, FlorenceAndrade Santana, Maria HelenaMaicas, SergiAndrade, FernandoMaidannyk, ValentynAndreev, Dmitriy NikolaevichMaier, Stelian SergiuAndreeva, MarinaMaina, TheodosiaAndrei, Radu DorinMainini, FrancescoAndreoli, EnricoMaior, IoanaAndreu, InmaculadaMair, Lamar O.Andreu, IreneMaitarad, PhornphimonAndreuzzi, EvaMaiti, SampaAndrianov, AlexanderMaiz, JonAndrianova, MariiaMaj, MalgorzataAndronic, LuminitaMajewska, EwaAndrushchenko, ValeryMajireck, MaxAndryushin, Konstantin P.Majolino, DomenicoAngeletti, Paolo MatteoMakarova, Anna S.Angeli, AndreaMakhaev, Viktor D.Angelici, GaetanoMakinde, Oluwole D.Angelini, PaolaMakosza, MieczyslawAngelopoulou, AthinaMakridakis, ManousosAngelov, BorislavMakris, Dimitris P.Angelov, GeorgeMaksimchuk, NataliyaAngelova, AngelinaMaksimović, JelenaAngelova, SilviaMakuch, EdytaAngelovski, GoranMakunga, Nokwanda P.Angius, FabrizioMakwana, KamleshAngsantikul, PavimolMalafronte, AnnaAniceto, NatáliaMalagodi, MarcoAnimitsa, IrinaMalaka, RatmawatiAnioł, MirosławMalander, SusanneAnisuzzaman, AnisuzzamanMaleczka, Robert E.Anne-Marie, CiobanuMalenović, AnđelijaAnnibal, AndreaMalfa, Giuseppe AntonioAnokwuru, Chinedu ProsperMalfanti, AlessioAnouar, AlamiMalik, MagdalenaAnsari, PrawejMalik, Muhammad ImranAntal, DianaMalikan, MohammadAntal, IstvánMalik-Gajewska, MagdalenaAntoine, Marie-HélèneMaliňák, DávidAntoniewska, AgataMalkin, AlexanderAntonini, GiovanniMallamace, FrancescoAntonio Marinas-Pardo, LuisMallamaci, RosannaAntonov, Alexander S.Mallandrich, MireiaAntonov, LiudmilMalm, AnnaAntunes, AlexandraMalmström, LarsAntunes, DulceMaluleka, Marole M.Antunes, VítorMalviya, GauravAnwander, ReinerMamane, VictorAnwer, KhalidMambu, LengoAnyszka, RafałMamun-Or-Rashid, A. N. M.Aoyagi, ShinobuMan, AdrianAparicio, Francisco J.Man, SimonaApetrei, ConstantinManabe, YukiApfelbaum, Evgeny M.Manaenkov, OlegApostol, Theodora-VeneraManakhov, AntonAppathurai, Narayana P.Manan, Rajith SinghAppell, MichaelManavalan, BalachandranAppendino, GiovanniMancini, InesAprile, CarloMancini, Pedro M. E.Aprodu, IulianaMandal, Tapas K.Aragay, GemmaMandalari, GiuseppinaArakawa, YukiMandić, VilkoArancibia, RodrigoMandrioli, RobertoAranda, AgustínManetti, MirkoArandes, José M.Máñez, SalvadorAraújo, Alberto N.Manfra, MicheleAraujo, JeffersonManfredi, Kirk P.Araújo, João M. M.Manfredini, StefanoAraújo, Maria EduardaMangalagiu, IonelAraújo, Thaíse GonçalvesMangiaterra, GianmarcoAraújo-Ferreira, Arthur GustavoMani, VasudevanAraya-Hermosilla, Esteban AlejandroManiam, SubashaniAraya-Hermosilla, RodrigoManicum, Amanda-Lee EzraAraya-Maturana, RamiroManiewska, JadwigaArbour, ChristineManiglia, BiancaArce, Pedro F.Manini, PaolaArcone, RosariaMannell, HannaArczewska, MartaMannino, GiuseppeAreche, CarlosManolikakes, GeorgArellano, María EvaristaManouras, TheodorosArena, GiuseppeManousi, NataliaArena, KatiaManríquez-Torres, José De JesúsArenas, Luis FernandoMansikkamäki, AkseliAresta, AntonellaMantell, CasimiroArgitis, PanagiotisMantellini, FabioAriadna Thalía, Bernal-MercadoManyakhin, Artem YuArias Santiago, Salvador AntonioManyes, LaraArif, TasleemManzoni, ViníciusAriga, HiroyoshiMao, ZongwanArjmandi, RezaMarabello, DomenicaArmentrout, Peter B.Marabotti, AnnaArmetta, FrancescoMaragou, Niki C.Arnaboldi, SerenaMaramai, SamueleArnal-Herault, CaroleMarasco, GiovanniArnason, JohnMarchand, Patrice A.Aronica, Laura AntonellaMarchetti, FabioArosio, DanielaMarchetti, NicolaArosio, PaoloMarchionni, AndreaArrico, LorenzoMarcinčáková, DanaArrieta, JesúsMarcocci, Maria ElenaArrigo, CiceroMarco-Contelles, Jose LuisArrigoni, ChiaraMarcos, Paula M.Arroba, Ana I.Marczak, WojciechArsiccio, AndreaMare, RosarioArtem’ev, Alexander V.Mareev, EvgeniiArthur, ChrisMarengo, MauroArturo-Perdomo, David E.Margalho, CláudiaArulvasu, ChinnasamyMarginǎ, DenisaArun, YuvarajMargulies, BarryArunagiri, AnoopMaría Evarista, ArellanoArunaksharan, NarayanankuttyMaria, Teresa M. R.Arvanitis, DemetriosMaria-Ferreira, DanieleArzmi, Mohd HafizMarian, EleonoraAsa’ad, FarahMariani, PaoloAsadipooya, KamyarMariappan, KadarkaraisamyAsahara, HaruyasuMarichev, KostiantynAsbell, Penny A.Marietta, FodorAscheri, JoseMarimuthu, ParthibanAsfin, Ruslan E.Marimuthu, ThashreeAsgher, MohdMarin, EliaAshley, JonMarín, Josefa IsasiAshour, Mohamed LotfyMarinelli, EnricoAshraf, Syed AmirMarinescu, MariaAshrafi, AmirmansoorMarini, FedericoAshton, TrentMarinoiu, AdrianaAspri, MariaMarinov, Daniil L.Assaf, Khaleel IbrahimMarinova, KrastankaAssalin, Marcia ReginaMaris, LaubertsAstl, JaromírMariychuk, RuslanAtamas, NataliaMarkiewicz, RoksanaAtanase, LeonardMarkov, AndreyAtanasova, MariyanaMarks, MichaelAtanassova, MariaMarkuszewski, Michal JanAtencio, DanielMarlin, NathalieAthanasiadis, VassilisMarmiroli, NelsonAtia, Mohamed A. M.Marosvolgyi, TamasAtobe, MasakazuMaroušková, AnnaAtrián-Blasco, ElenaMarová, IvanaAttia, YoussefMarques, CarolinaAttila, Papp LajosMarques, Joana A.Aturki, ZeinebMarques, M. MatildeAtzori, CesareMarquez, RonaldAuewarakul, PrasertMárquez-Miranda, ValeriaAuguet, TeresaMarra, AlbertoAussenac, ThierryMarracci, SilviaAvellone, GiuseppeMarszalek, MartaAvena-Bustillos, RobertoMárta, LadányiAverina, Elena B.Martella, GiuseppinaAvetissov, IgorMartens, JürgenAvgeropoulos, ApostolosMartha C., Rosales-HernándezAvila-Nava, AzaliaMartí, SergioAvilés, María DoloresMartin Cordero, CarmenAvilla, JesúsMartin Solans, SantiagoAvin, VijayMartin Vogt, MartinAviñó, AnnaMartin, Hans‐PeterAvitia-Domínguez, ClaudiaMartin, James D.Avolio, RobertoMartin, Jan M. L.Avramopoulos, AggelosMartin, JulietteAvrutina, OlgaMartin, NeginAwate, SanketMartin, RachelAyad, Miriam F.Martina, KatiaAyala-Zavala, Jesus FernandoMartinelli, JonathanAydillo, CarlosMartín-Escolano, RubénAyora-Cañada, María JoséMartinez Alonso, MartaAyora-Talavera, TeresaMartinez Gonzalez, Jose AngelAyub, RabiaMartinez Hernandez, EliasAyuso-Fernandez, IvanMartínez, AlbertoAzad, Mohammad A.Martínez, AnaAzad, Obyedul KalamMartínez, GemaAzaizeh, HassanMartínez, SidoniaAzam, FaizulMartínez-Campa, CarlosAzevedo Batista, AlzirMartínez-Carballo, ElenaAzevedo, LucianaMartinez-Cuezva, AlbertoAzizi, AliMartinez-Fernandez, LaraAzizkhani, MaryamMartínez-García, MarcosAzov, VladMartinez-Gonzalez, Jose AdrianBaba, KoumeiMartínez-Huerta, María VictoriaBabor, MartinMartinez-Martinez, Antonio J.Baby, André RolimMartínez-Navarrete, GemaBacelar, EuniceMartínez-Núñez, Mario AlbertoBach, HoracioMartínez-Rodríguez, SergioBach, Thomas J.Martinez-Vazquez, MarianoBąchor, RemigiuszMartín-Gil, JesúsBäcker, DanielMartinho, José Manuel GasparBácskay, IldikóMartinho, MarlèneBacso, ZsoltMartini, DanielaBączek, KatarzynaMartini, FlorenciaBaczyńska, DagmaraMartiniaková, MonikaBadawi, MichaelMartino, MarcoBadea, MihaelaMartinotti, SimonaBadea, Silviu-LaurentiuMartin-Pendas, AngelBader, OliverMartins, AliceBadía Laíño, RosanaMartins, ArturBadr, AhmedMartins, CatiaBae, Ji-YeongMartins, DianaBae, Jung-WooMartins, NatáliaBae, TaeokMartí-Rujas, JavierBaeza Baeza, Juan JoseMarturano, ValentinaBaeza, AlejandroMartuscelli, MariaBagdziunas, GintautasMaruca, AnnalisaBagetta, GiacintoMarucci, GabriellaBagi, PéterMarušič, MajaBagnoli, LuanaMaruyama, KeiBahadur, AliMarverti, GaetanoBahuguna, AshutoshMaryunina, KseniyaBai, FangMarzinek, JanBai, PengMarzo, TizianoBai, ShuhuaMarzullo, Vincenzo ManuelBai, YuxingMasci, MaurizioBaiardi, AlbertoMascia, GiuseppeBaik, Ku YounMascotto, SimoneBailly, ChristianMasek, AnnaBaj, TomaszMäser, PascalBajda, TomaszMasi, AnnalisaBajguz, AndrzejMasi, MarcoBajusz, DávidMaslak, Veselin R.Bak, AndrzejMasłoń, AdamBakker, HansMasłyk, MaciejBakulina, Olga YuMassa, AntonioBalabanova, VesselaMassari, SerenaBalar, NrupMassoud, SalahBalaș, MihaelaMassue, JulienBalashov, Victor N.Mastana, SarabjitBalawejder, MaciejMasternak, JoannaBalažová, AndreaMasters, EliotBald, IlkoMasu, HyumaBaldassari, SaraMasud, JahangirBaldino, LuciaMasui, ToshiyukiBaldisserotto, AnnaMasullo, MilenaBaldoví Jachán, José JaimeMatamoros, Manuel A.Balestra, DarioMatarrese, PaolaBalicki, SebastianMatassini, CamillaBalijapalli, UmamaheshMatencio, AdriánBalintova, MagdalenaMateo, Carmen ReyesBalla, AndrásMateo, CesarBallauff, MatthiasMaterska, MałgorzataBallaz, Santiago J.Mathé-Allainmat, MoniqueBallesté, Ruben MasMathieu, EtienneBallesteros-Garrido, RafaelMathieu, MarchivieBallesteros-Vivas, DiegoMathivathanan, LogeshBallinger, JimMathiyalagan, PrabhuBalogh, György TiborMatias De Alencara, SeverinoBaltakys, KęstutisMatić, AnitaBaltazar, Luís Gonçalo CorreiaMatijašić, GordanaBalzan, SilvanaMatin, AnaBalzano, FedericaMatino, IsmaelBalzaretti, Claudia MariaMatkowski, AdamBalzer, FrankMato, SusanaBambach, MikeMatos, ManuelBanchelli, MartinaMatović, BrankoBanchero, MauroMatras-Postolek, KatarzynaBandarenka, HannaMátravölgyi, BélaBandow, KenjiroMatsakidou, AnthiaBandyopadhyay, DebasishMatsoukas, ThemisBandyopadhyay, SmarakMatsuda, FumioBanerjee, AnilMatsumura, KiyoshiBang, HyoweonMatsumura, MioBankova, VassyaMatsuo, YukikoBanožić, MarijaMatsushita, YasuyukiBanskota, Arjun H.Matsuya, YujiBañuelos, JorgeMattan, HurevichBao, XiucongMattei, CésarBao, YinyinMattei, FabrizioBao, YongmingMatthews, LorinBaptista, RafaelMattonai, MarcoBar, SukantaMatula, GrzegorzBarajas-Solano, AndresMatusevičius, PauliusBaranac-Stojanović, MarijaMatwijczuk, ArkadiuszBarančík, MiroslavMatziari, MagdaliniBaranek, PhilippeMaurer, StefanieBaranova, EkaterinaMauri, MicheleBaranowska-Kuczko, MartaMauriello, GianluigiBarao, Carlos E.Mauro, Maria VittoriaBarata, Joana F. B.Mauro, MatteoBarba, CarmenMauro, VasconiBarba, IgnasiMaury-Ramírez, AníbalBarbagallo, DavideMauša, GoranBarbara, BreznikMavri, JanezBarbaric, MonikaMavrikou, SofiaBarbarossa, AlexiaMaxwell, AnthonyBarbasiewicz, MichalMayans, JuliaBarbato, MaurizioMayer, ChristianBarberis, ElettraMayer, Rupert L.Barbero, NadiaMayer-Gall, ThomasBarbinta-Patrascu, Marcela ElisabetaMayhan, William G.Barbon, SilviaMaykel, González-TorresBarbosa, ArmenioMazerbourg, SabineBarbosa, Helene SantosMazhukin, DmitriiBarbosa, JoanaMaziejuk, MirosławBarbosa, Raquel De MeloMazivila, SarmentoBarbu, VasilicaMazur, Antonina JoannaBarcena, JuanMazur, MarcelinaBarderas, RodrigoMazur, MichalBarding, Gregory A.Mazzanti, MicheleBari, EliaMazzarino, MonicaBarić, DanijelaMazzoccanti, GiuliaBarnard, AnnaMazzone, GloriaBarodia, Sandeep KumarMazzotta, SarahBaron, ByronMbita, ZukileBaron, FrédéricMcCall, Laura-IsobelBarone, RobertoMcCarthy, PumtiwittBarradas, Thaís NogueiraMcDonald, PeterBarreales, Eva G.McGouran, JoannaBarreca, DavideMcgregor, NeilBarreca, MariliaMcKay, TinaBarreca, SalvatoreMd, ShadabBarresi, ElisabettaMechoulam, RaphaelBarreto, Maria CarmoMedana, ClaudioBarroca, Maria JoãoMedeiros, Adriane Bianchi PedroniBarros, Anerise DeMeder, RogerBarroso Flores, JoaquínMedina Juarez, Luis AngelBarroso, HelenaMedina-Ramirez, AdrianaBarsanti, LauraMedio-Simón, MercedesBarsyte-Lovejoy, DaliaMedved’Ko, Alexey V.Bartas, MartinMedvidović-Kosanović, MartinaBartashevich, EkaterinaMeeta, GeraBartczak, PiotrMeghea, AureliaBartnik, MagdalenaMeghil, MohamedBartoli, MattiaMegia-Fernandez, AliciaBartolini, ManuelaMehbub, Mohammad FerdousBartoloni, Fernando H.Meini, Maria-RocioBarton, Larry L.Meiramkulova, KulyashBartosz, GrzegorzMejía, AndrésBartoszak-Adamska, ElżbietaMelchiorre, DanielaBartuzi, DamianMelgar C., BrunoBarwiolek, MagdalenaMelguizo-Guijarro, ManuelBaryeh, KwakuMelilli, Maria GraziaBaś, SebastianMelin, FredericBasarić, NikolaMellin, PatriciaBaschieri, AndreaMello, Paula Homem DeBasiak, EwelinaMelo, AndréBasile, LiviaMelo, Priscilla SiqueiraBasílio, NunoMemisoglu, GorkemBaskar, VenkitasamyMena Rejón, Gonzalo JoaquínBaskaran, RathinasamyMena-Rejon, Gonzalo J.Bassani, AndreaMendes, FilipaBassetti, MauroMéndez Bolaina, EnriqueBassi, Ana PaulaMendez, FranciscoBassolé, Imaël Henri NestorMendez-Cuadro, DaríoBassolino, LauraMendez-Rojas, MiguelBastola, EbinMéndez-Sánchez, DanielBastos, Erick LeiteMendiola, JoseBastos, Mônica MacedoMendonça, DinaBastos, VerónicaMendoza, SandraBathgate, RoslynMenegazzi, MartaBatina, NikolaMeneghetti, FiorellaBatista, RamonMenendez, DanielBatista-Duharte, AlexanderMenéndez, EstherBattaglini, NicolasMeneses, CarlosBaumgarten, MartinMenezes, Cristiano Ragagnin DeBauzá, AntonioMenezes, Irwin Rose AlencarBavaresco, LuigiMeng, GeBaykov, SergeyMeng, Ling-HongBayón, RocíoMenger, Fredric M.Bazarra, Diego AngostoMenges, Fabian S.Bazwinsky-Wutschke, IvonneMenghini, LuigiBazzicalupo, MiriamMensah, Enoch A.Bazzocchi, Isabel LópezMenyhárd, Dóra K.Beale, DavidMenzorov, Aleksei GBębenek, EwaMerah, OthmaneBeberok, ArturMercante, LuizaBec, Krzysztof B.Merighi, StefaniaBecerra, DianaMerino, GabrielBecher, GeorgMerino-Montiel, PenelopeBechtold, ThomasMerkulov, Daniela ŠojićBeckett, Michael A.Merle, NicolasBecq, FredericMerola, Joseph S.Bednarek, RadoslawMeslet-Cladiere, LaurenceBednarski, HenrykMessina, GiovanniBegines, BelenMessore, AntonellaBehrends, VolkerMeštrović, SenkaBehringer, ErikMethling, KarenBeklemishev, MikhailMettin, RobertBeld, JorisMeyer, FranckBełdowski, PiotrMézes, MiklósBelenguer-Sapiña, CarolinaMezhuev, Ya. O.Beletskaya, Irina PetrovnaMezzetta, AndreaBelkova, Natalia V.Mi, Fwu-LongBellia, FrancescoMi, LanBellich, BarbaraMian, CaterinaBellmann-Sickert, KathrinMiao, LinglingBello, MartinianoMiao, YinglongBello, Nicholas T.Miazga-Karska, MalgorzataBello-Bello, Jericó JabínMicale, NicolaBellucci, StefanoMiccheli, AlfredoBeloglazkina, Elena K.Michaelis, MonikaBelousov, YuryMichalczyk, MariuszBelova, VeraMichálek, MartinBelpassi, LeonardoMichalska, KatarzynaBeltran, María Del Carmen SánchezMicheau, OlivierBelvedere, RaffaellaMichelacci, Yara M.Belyakov, SergeyMichelle, PalmieriBen Kaab, SofieneMichlewska, SylwiaBenaglia, MaurizioMichon, ChristopheBenarba, BachirMicillo, RaffaellaBenčić, ĐaniMickevičius, VytautasBencini, AndreaMicov, AnaBende, AttilaMicutz, MarinBenedé, Juan L.Miękus, NataliaBenfeito, Sofia EsterMielańczyk, AnnaBenfenati, EmilioMiele, RossellaBéni, SzabolcsMielke, Howard W.Benković, MajaMiguel Hernández, BeatrizBensalah, NasrMiguel, Maria Da Graça CostaBento, FatimaMiguel, Trinidad DeBenyhe, SándorMihailović-Stanojević, NevenaBerchová-Bímová, KateřinaMihajlovski, KatarinaBerestetskiy, Alexander O.Mihal’, MárioBeretta, GiangiacomoMihály, JudithBerillo, DmitriyMihaylova, VeronikaBerkecz, RobertMikami, MikioBerlier, GloriaMikes, JaromirBernabé-Antonio, AntonioMikolajczak, PrzemyslawBernard, Marek K.Mikołajczyk, TomaszBernardini, ChiaraMikołajczyk-Bator, KatarzynaBernardini, GiuliaMilán-Carrillo, JorgeBernardino, SusanaMilanese, ChiaraBernardo, SaraMilanova, AneliyaBernascon, LeonardoMilanović, MarijaBernátová, IvetaMilardi, DaniloBernatová, SilvieMilcic, MilosBernhagen, JürgenMilella, LuigiBernhard, WolfgangMiler, MarkoBernhardt, AnneMileva, ElenaBernstein, NiritMileva, MilkaBerrada, HoudaMilic, NatasaBerrino, EmanuelaMilicevic Sephton, SelenaBershitsky, Sergey Y.Milikić, JadrankaBertani, RobertaMilinčić, Danijel D.Bertelli, DavideMillán, ÁngelBerto, SilviaMillán-Martín, SilviaBertoldo, Jean BorgesMiller, GlennBertsouklis, KonstantinosMiller, ReinhardBertuzzi, TerenzioMilosavljević, Milutin M.Berzins, AgrisMilosevic, NatasaBęś, AgnieszkaMiloso, MariarosariaBesalú, EmiliMimica-Dukic, NedaBeslo, DragoMimura, JunseiBest, IvanMin, HyeminBest, StephenMin, Kil SikBettencourt, Ana FranciscaMin, Kyung-SanBeyeh, Ngong KodiahMin, Sun-JoonBezuglov, Vladimir V.Min, Tae-SunBhalothia, DineshMinaev, Boris F.Bhanage, Bhalchandra M.Minami, YasunoriBhandari, Shiva RamMinato, Ken-ichiroBharadwaj, ShivMincheva, RosicaBhattacharyya, SurjenduMine, YuichiBhullar, Khushwant SinghMineev, KonstantinBhusal, RamMing, WuyiBiałek, AgnieszkaMinnelli, CristinaBian, XiaoyingMinorics, RenátaBian, YongzhongMintcheva, NeliBian, YueminMinto, Robert E.Bianchini, FrancescaMinutolo, FilippoBianco, ArmandodorianoMiralles, PabloBiancolillo, AlessandraMiranda, AdelaideBidel, Luc P. R.Miranda, ClaudioBiegańska-Marecik, RóżaMiranda, CristobalBielejewski, MichalMiranda, KatrinaBieliński, Dariusz M.Miranda, MarianaBiely, PeterMiranda, PauloBienvenut, WillyMiras, Haralampos N.Biernasiuk, AnnaMircea, CorneliaBijak, MichałMirković, MiljanaBikić, SinišaMiroshnyk, InnaBil, MonikaMišan, AleksandraBilal, MuhammadMisawa, TakashiBild, WaltherMiseta, AttilaBilewicz, AleksanderMishra, Awdhesh KumarBilia, Anna RitaMishra, MeerambikaBilican, DogaMishra, RohitBiljan, IvanaMishra, RosalinBillotey, ClaireMishra, Sanjay R.Bin, JunMishra, SanketBin, XuMishra, VibhorBinda, ClaudiaMishyna, MaryiaBindra, Jasleen KaurMiskulin, MajaBinkowska, IwonaMitaine-Offer, Anne-ClaireBirková, AnnaMitakou, SofiaBíró, LindaMitani, TakakazuBis, ZbigniewMitić-Ćulafić, DraganaBischof, JohannesMititelu Tartau, LilianaBisi, AlessandraMitra, SwarupBiziuk, Marek K.Mitran, GheorghiţaBizzarri, Bruno MattiaMitrović, MiroslavaBjelić, AnaMitsuhashi, RyojiBlack, DavidMittova, Irina YaBlaga, Alexandra CristinaMitulovic, GoranBlagodatski, ArtemMiu, DanaBlaheta, RomanMiura, NobuakiBlamires, SeanMiyamae, YusakuBlancaflort, LluísMiyashita, MasahiroBlanco, Francisco J.Miyatake, HideyukiBlanco, IgnazioMiyoshi, DaisukeBlanco, MatíasMleko, StanisławBlanco, PilarMlinarić, SelmaBlasiak, JanuszMlostoń, GrzegorzBlay, GonzaloMlynarczyk, DariuszBlazevic, IvicaMnif, WissemBlázquez, EnriqueMó, OtiliaBleilevens, ChristianMoad, GraemeBloise, EnrricoMobbili, GiovannaBlossey, RalfMoccia, StefaniaBlough, Bruce E.Mochane, Mokgaotsa JonasBobba, Kondapa NaiduMocioiu, Oana CătălinaBober-Majnusz, KatarzynaModak, BrendaBobrovsky, AlexeyMoghtadernejad, SaraBocchetta, PatriziaMohajernia, ShivaBoccia, Antonella CaterinaMohamed, Abd El-Moez A.Bocian, SzymonMohamed, SharmarkeBöckmann, LarsMohammad, AkbarBocos-Bintintan, VictorMohammed, AltafBodo, EnricoMohammed, HamdoonBoeglin, AlexMohammed, NameerBoffo, Elisangela F.Mohammed, Salman Afroze AzmiBogado, André L.Mohan, MoodBogani, LapoMohles, VolkerBogdanov, Alexey M.Mohr, FabianBoguń, MaciejMojzych, MariuszBogusławska, JoannaMokhodoeva, OlgaBoguszewska, KarolinaMolčanov, KrešimirBohinc, TanjaMołczan, MarekBöhm, VolkerMoldoveanu, CostelBojarska-Junak, Agnieszka A.Moldoveanu, Serban C.Bojić, MirzaMoler, Jose AntonioBojko, AgnieszkaMolina Salinas, Gloria MaríaBokach, NadezhdaMolinari, NicolaBolaño, SandraMoliner Martinez, YolandaBolanos-Garcia, Victor M.Moliner, AnaBolboaca, SoranaMolíns-Legua, C.Bölcskei, HedvigMollica, AdrianoBoldyreva, ElenaMolnar, OleksandrBolla, Pradeep KumarMoloney, Mark G.Bolotin, Dmitrii S.Momeny, MajidBolt, AliciaMondal, Amit KumarBon, VolodymyrMondal, SantanuBonardd, SebastianMondeshki, MihailBonasia, AnnaMondol, TanumoyBondžić, Aleksandra M.Monfort, AmparoBonferoni, Maria CristinaMonge, DavidBongiovanni Abel, SilvestreMonné, MagnusBonilla-Cruz, JoséMonobe, HirosatoBonnet, PascalMontagud-Romero, SandraBonnet, Pierre-AntoineMontalvo González, EfigeniaBonomo, MatteoMontaño, Gabriel A.Bonvicini, FrancescaMontecucco, FabrizioBonyár, AttilaMonteiro André De Oliveira, Maria Da ConceiçãoBoothello, RioMonteiro, Jorge H.Bora, AlinaMonteiro-Neto, ValérioBorbas, AnikoMontenegro, LuciaBorbas, K. EszterMontero, OlimpioBorch, Troels HolzMontero, SalvadorBordes, ElisabethMontes, EnriqueBorges Cabrera, MilkosMontes, GuilhermeBorges, AlissonMontgomery, Henry EdwardBorges, DanielMonti, DanielaBorges, LigiaMonti, DonatoBorges, RicardoMontone, AngelaBorghi, FrancescaMoon, Geon DaeBorgna, FrancescaMoon, Sung-KwonBorgonovo, GigliolaMoore, KarenBorja, Mark S.Morabito, RossanaBorniger, Jeremy C.Moraes, Caroline CostaBoroń, DariuszMoraes, Valéria R. S.Boros, LászlóMorais Ruela, André LuísBorota, AnaMorais, Tânia S.Borowicz, MarcinMorak-Młodawska, BeataBorowiec, JoannaMorales, PaulaBorowiec, Magdalena SłowikMorales-González, José AntonioBorrajo, PaulaMorales-Rojas, HugoBortolus, MarcoMorante-Zarcero, SoniaBorymska, WeronikaMoravec, ZdeněkBorysiak, SlawomirMorbidelli, LuciaBoscaro, ValentinaMoreira, Daniel CarneiroBosch, ChristineMoreira, Felismina T. C.Boschmann, MichaelMorel, MauricioBoselli, EmanueleMorelli, SabrinaBoshkov, NikolaiMoreno, AndrésBoskou, GeorgeMoreno, EstefaníaBossi, AlbertoMoreno, Juan J.Botana, Ana MaríaMoreno, Maria JoãoBotas, Alexandre M. P.Moreno, Paulo Roberto H.Botelho, Maria FilomenaMoreno-Alcántar, GuillermoBotelho, Raquel Braz AssunçãoMoretti, MassimoBotez, ElisabetaMorgan, MichaelBotta, BrunoMorgenstern, JakobBotta, ElenaMori, HirotadaBottone, Maria GraziaMori, MatteoBoualy, BrahimMorianos, IoannisBoucher, DaveMorikawa, KyojiroBoudier, ArianeMorimoto, JoshuaBougea, AnastasiaMorimoto, MasakazuBoukir, AbdellatifMorimoto, MasanoriBoulatov, RomanMorini, LucaBoulon, Marie-EmmanuelleMorisawa, YusukeBoumba, VassilikiMorita, KentaBouquillon, SandrineMorita, KyojiBourgoin, SandrineMorita, NobuyoshiBourin, MichelMorley, Barbara J.Bouropoulos, NikolaosMorocho-Jácome, AnaBoushaki, ToufikMorozov, Vladimir N.Bousta, DalilaMorris, JamesBouvier, BenjaminMorvan, FrançoisBowman, MichaelMorzycki, Jacek W.Boyarskiy, Vadim P.Mosa, IslamBoyatzis, StamatisMosca Angelucci, DomenicaBoye, AlexMoschini, RobertaBožović, MijatMoseke, ClausBozzelli, Joseph W.Moseley, HunterBrachner, AndreasMosnackova, KatarinaBradshaw, PatrickMot, AugustinBraga, Anna Rafaela CavalcanteMotrescu, IulianaBraga, Maria-HelenaMotta Neves, RobertaBran, José A.Motta, Chiara MariaBranca, FerdinandoMouga, TeresaBrancato, VirginiaMouinga-Ondémé, AugustinBrand, IzabellaMounir, BendahmaneBranowska, DanutaMoussaoui, YounesBraovac, SusanMoya, Antonio A.Bratskaya, SvetlanaMoyá, María LuisaBratu, AnaMoya-Ramírez, IgnacioBrauner, ChristianMozaffari, SaeedBraveenth, RamanaskandaMozo Llamazares, Juan DanielBravi, ElisabettaMpupa, AneleBravo-Sanchez, Micael GerardoMravljak, JanezBrčić Karačonji, IrenaMráz, JaroslavBrea, Jose ManuelMroczkowska-Szerszeń, MajaBreaz, Radu-EugenMroczyńska, KarinaBrebu, MihaiMrówczyńska, LucynaBredikhin, Alexander A.Mu, LinBregier‐Jarzębowska, RomualdaMuceniece, RutaBrehm, MartinMück-Lichtenfeld, ChristianBrejnrod, AskerMucsi, ZoltánBrekalo, IvanaMueller, Thomas J. J.Bren, UrbanMueses, Miguel ÁngelBrender, JeffreyMughal, AmreenBreza, MartinMukai, TakahitoBriançon, StéphanieMukherjee, NirmalyaBrichacek, MatthewMukhopadhyay, TufanBridi, RaquelMulas, MaurizioBrinck, ToreMulero, MiquelBrisset, HuguesMuller, ChristianBritton, JonathanMüller, JoachimBrocławik, EwaMuller, LaurentBroda, Małgorzata A.Müller, NorbertBronowska, AgnieszkaMüller, SimonBrooks, AllenMüller, ThomasBrooks, Benjamin D.Müller-Vahl, Kirsten R.Brophy, Joseph JohnMunekata, Paulo E. S.Brouard, IgnacioMuniz, JuanBrovarets, Volodymyr S.Muñiz-Ramirez, AlethiaBrown, Alan B.Muñoz, AmnerBrown, DennisMuñoz, Marcelo AndrésBrown, Janet E.Muntean, Dana MariaBrown, LindsayMuntean, DeliaBruce, JamesMunteanu, Alexandra CristinaBruijn, Johannes DeMunteanu, CristianBrullo, ChiaraMunteanu, Florentina-DanielaBrun, NicolasMuntyan, Maria S.Brunel, DamienMunusamy, ElangoBrunetti, LeonardoMurakami, TsutomuBruno, VictorMurakami, YasufumiBruns, Carson J.Murale, Dhiraj P.Bruns, RoyMurati, TeutaBruschini, LucaMurcia Mesa, Julie JoseaneBrushi, Marcos LucianoMurcia, PedroBrycki, BogumilMuresan, VladBryja, LeszekMurgia, Marco A.Bryndal, IwonaMurias, MarekBrys, JoannaMurkovic, MichaelBrzeziński, KrzysztofMurphy, ShonaBrzeziński, MichałMurray, Jane S.Bucić-Kojić, AnaMurray, KeithBuckin, VitalyMurtaza, GhulamBuda, ValentinaMurtinho, DinaBuda, Valentina OanaMurzakhanov, Fadis F.Budai-Szűcs, MáriaMurzin, DmitryBūdienė, JurgaMusgrave, IanBudyka, Mikhail F.Musiał, MałgorzataBudzisz, ElżbietaMusiał-Kulik, MonikaBuema, GabrielaMusić, SvetozarBueno-Silva, BrunoMusile, GiacomoBufalieri, FrancescaMussagy, Cassamo UssemaneBugarcic, AndreaMusso, LoanaBuica, AstridMussury, Rosilda M.Buisson, DidierMustafa, AdnanBujak, TomaszMustafa, GhazalaBujak-Gizycka, BeataMustafa, Natali RianikaBujanovic, BiljanaMusumeci, ChiaraBule, MohammedMuszalska-Kolos, IzabelaBuljac, MašaMutalik, SrinivasBuller, JensMuthu, SambanthamBunce, Richard A.Muthukumar, KannanBundaleska, NeliMuthukumaran, JayaramanBunea, AndreaMylonas-Margaritis, IoannisBunev, AlexanderMyong Jae, YooBuoro, Rafael MartosMyongwon Lee, LuciaBuranelo Egea, MarianaNabae, YutaBurchacka, EwaNabais, PaulaBurcul, FrankoNadǎş, George CosminBures, FilipNaeimirad, MohammadrezaBurgalassi, SusiNafis, AhmedBurilov, AleksanderNag, PurbaBurket, JessicaNagae, MasamichiBurlacu, AlexandruNagai, JunkoBurrai, Giovanni PietroNagapudi, KarthikBurroni, LucaNagatoishi, SatoruBursic, VojislavaNagatsu, AkitoBurton, Aaron S.Nagatsugi, FumiBuryak, Aleksey K.Nagel, RaimundBusardo, Francesco PaoloNaghdi, SamiraBuschmann, AlejandroNagles, EdgarBushnev, Dmitriy A.Nagpure, Atul S.Bussetti, GianlorenzoNagulapalli Venkata, KalyanBustos, Diego MartinNagy, ElodBustos, Rosa HelenaNagy, EndreBusuioc, CristinaNagy, TiborBuszewski, BogusławNahain, AbdullahButoi, ElenaNaidu Chitrala, KumaraswamyBuzuk, MarijoNaimi-Jamal, Mohammad RezaBuzzá, Hilde HarbNainu, FirzanByerley, LauriNair, Sandeep SudhakaranBytešníková, ZuzanaNajlah, MohammadCaballero, AntonioNakagaito, Antonio NorioCaballero, IsabelNakagawa, YoshinaoCaballero-Casero, NoeliaNakagawa-Goto, KyokoCabib, SimonaNakajima, TasukuCabral, Benedito J. C.Nakamura, IsseiCabral, HamiltonNakamura, TatsuyaCabrera Orefice, AlfredoNakamura, YoshiyukiCabrera-Barjas, GustavoNakano, ShogoCabrera-González, JustoNakata, NorioCabrerizo, Franco M.Nakatani, YoshihikoCabrini, MarinaNakazawa, HidekiCacciola, FrancescoNakchbandi, InaamCacciola, Nunzio AntonioNallathamby, Prakash D.Cáceres Jensen, LizethlyNalvarte, IvanCadar, OanaNam, Il-WooCadelis, Melissa MichelleNam, Ki HyunCadenas Pliego, GregorioNam, Kung-WooCadinoiu, Anca NiculinaNam, Ok HyungCadiou, CyrilNaman, C. BenjaminCadwallader, Keith R.Nambo, MasakazuCaetano, WilkerNameni, GordonCaffrey, PatrickNamgaladze, DmitryCagno, ValeriaNanda, SatyabrataCahlíková, LucieNanno, MasanobuCai, LishengNano, AdelaCai, WenlongNaoto, ShimizuCaille, FabienNarayanankutty, ArunaksharanCaillol, SylvainNarcisa, MandrasCairns, Warren Raymond LeeNardi, MonicaCairrão, ElisaNarita, AkihiroCajal, YolandaNartop, PınarČakić Semenčić, MojcaNartova, Anna V.Cala, AntonioNash, RobertCalabrese, GiovannaNashchekin, Alexey V.Calabriso, NadiaNasr, FahdCalabrò, Paolo S.Nasreddine, RoubaCalapai, GioacchinoNastuta, Andrei VasileCalcaterra, AndreaNatale, PaoloCalcio-Gaudino, EmanuelaNath, SubhasisaCalderini, OrnellaNathubhai, AmitCalderón-Oliver, MarielNatu, RuchaCaleja, CristinaNavarra, MicheleCalina, DanielaNavarrete-Vazquez, GabrielCalinescu, IoanNavarro, ElisaCălinoiu, Lavinia FlorinaNavarro-Cruz, Addi RhodeCalín-Sánchez, ÁngelNavarro-Hoyos, MirthaCaliogna, LauraNaviglio, DanieleCalixto, Ana RitaNavizet, IsabelleCallahan, Brian P.Nawirska-Olszańska, AgnieszkaCallan, BridgeenNawrocka, AgnieszkaCallone, EmanuelaNawrot, BarbaraCalogeropoulou, TheodoraNaydenova, IzabelaCaltagirone, ClaudiaNazarian-Samani, MasoudCalvano, Cosima DamianaNazarova, AnastasiaCamacho-Alonso, FabioNazzaro, FilomenaCamacho-Ruiz, Rosa MaríaNde, Divine BupCamara, AntonioNdii, MeksianisCâmara, Niels Olsen SaraivaNeagu, Tiberiu-PaulCamele, IppolitoNebija, DashnorCamerel, FranckNechepurenko, Ivan V.Cametti, CesareNedbalcová, KateřinaCammas-Marion, SandrineNedialkov, Paraskev TodorovCammilleri, GaetanoNedyalkova, MiroslavaCampagna, RobertoNeels, OliverCampagne, Jean-MarcNeeraj, JoshiCampalans, AnnaNeffe, Axel T.Campanella, AlessandroNeffe, SlawomirCampbell, Fiona M.Negrea, PetruCampíns-Falcó, PilarNegri, ArmandoCampo, Maria LuisaNegut, IrinaCampos Rosa, Joaquín MaríaNehira, TatsuoCampos, MariaNeilson, AndrewCampos, Vinícius R.Nekvindova, PavlaCampos-Martin, Jose M.Nemukhin, AlexanderCampos-Montiel, Rafael G.Nenajdenko, Valentine G.Camposo Pereira, ArturNeo, Ana Maria GomezCamps, IhosvanyNeophytou, Christiana M.Canadanovic-Brunet, JasnaNervi, CarloCanas, SaraNesterkina, MariiaCandia, Modesto DeNesterova, Oksana V.Canela, Enric I.Netto, Theodoro A.Canela-Garayoa, RamonNettore, Immacolata CristinaCaneva, GiuliaNeugebauer, WitoldCanini, AntonellaNeunert, GrazynaCansian, Rogério LuisNeut, ChristelCanterini, SoniaNeves, JoãoCanuti, ValentinaNg, Ho LeungCao, HongdaNg, Tzi BunCao, HongnanNge, Thi ThiCao, YifengN’Gouemo, ProsperCapacchione, CarmineNguyen, AnhCapasso, RaffaeleNguyen, Ha Vinh LamCapel, JuanNguyen, Hanh ThuyCapon, RobertNguyen, LeeCappellacci, LoredanaNguyen, Ngoc A.Cappillino, PatrickNguyen, ThienCăprărescu, SimonaNi, KaiyuanCaprari, ClaudioNiaura, GediminasCaprioglio, DiegoNicasio, Maria CarmenCapuano, RosamariaNicholas, Aaron D.Caputo, GregoryNickels, Michael L.Cara, EleonoraNico, LinggCarabineiro, SóniaNicolai, EleonoraCara-Jiménez, JorgeNicolai, MarisaCaramujo, Maria JoséNicolas, CyrilCarceller, HectorNicolay, PascalCardá, MiguelNiculau, Edenilson Dos SantosCardador-Martínez, AnabertaNie, ChuanxiongCardea, StefanoNiedzielski, PrzemysławCardellicchio, CosimoNiedzwiecki, LukaszCárdenes, VíctorNielsen, MogensCardia, Maria CristinaNielsen, Vance G.Cardiano, PaolaNiemann, Hartmut H.Cardinali, Daniel P.Niemczak, MichalCardoso, Sílvia AlmeidaNieoczym, DorotaCardoso, Susana M.Nieto, PedroCardoso, VitorNieuwoudt, MichelCardullo, NunzioNiidome, TakuroCarella, Angelo M.Nikas, Ilias P.Cariello, MaricaNikitin, DaniilCarignani, ElisaNikodinovic-Runic, JasminaCarla, QueirósNikolaev, EugeneCarlini, Célia R.Nikolaevskii, Stanislav A.Carlos Salay, LuizNikolaidis, NikolaosCarlotto, SilviaNikolic, LjubisaCarmeli, ShmuelNikolić, Vladimir B.Carmen, MejíaNikovics, KrisztinaCarmine, ColucciniNilsen, AaronCarmona-Ribeiro, Ana M.Nilsen-Hamilton, MaritCarniato, FabioNimse, Satish BalasahebCarocho, Márcio SoaresNirala, Niraj K.Carotenuto, GianfrancoNishi, KosukeCarović-Stanko, KlaudijaNishinaga, TohruCarpentieri, AndreaNishitsuji, ShotaroCarpes, Solange TeresinhaNishiyama, YusukeCarr, Stephen B.Nisi, StefanoCarrapiso, Ana I.Nissapatorn, VeeranootCarrasco, JaimeNisticò, Steven PaulCarreira-Barral, IsraelNitride, ChiaraCarrera Cerritos, RaúlNitulescu, George MihaiCarrera Fernández, CeferinoNitulescu, MihaiCarrieri, AntonioNitzsche, BiancaCarrilho, Rui M. B.Niu, TianhuaCarrillo-López, Luis M.Niu, XuemeiCarrillo-Parra, ArtemioNixon, GemmaCarroll-Portillo, AmandaNoble, JenniferCarrotta, RitaNobre, Marcos Augusto LimaCarrubba, AlessandraNoce, AnnalisaCarta, PaolaNoda, MasafumiCarullo, GabrieleNoel, FrançoisCaruntu, AnaNogara, PabloCaruso, GianlucaNoguchi, YoshihikoCarvajal-Millan, ElizabethNogueira Perez, Juan JoseCarvalho, AngelaNogueira, AntónioCarvalho, José Carlos TavaresNoguera, LuisCarvalho, Lucas BragançaNoman, EfaqCarvalho, LuísaNomngongo, Philiswa NosizoCarvalho, MartaNonoguchi, HiroshiCasadonte, DominickNorman, TrevorCasapullo, AgostinoNorton, Amie E.Casaril, AngelaNosova, EmiliaCasasnovas, RodrigoNovelli, EnricoCaschera, DanielaNovembre, DanielaCascioferro, StellaNovikov, AlexanderCascone, GiuseppeNovikov, ValentinCásedas, GuillermoNovikova, SvetlanaCaseli, LucianoNovo, LuísCasella, GirolamoNovopashina, DaryaCaseri, WalterNowacka, MariaCasertano, MarcelloNowaczyk, JacekCassani, JuliaNowak, RafałCastaldo, RacheleNtaikou, IoannaCastañeda, Javier Eduardo GarciaNtanasis-Stathopoulos, IoannisCastañeda‐Corral, GabrielaNtie-Kang, FideleCastañón, EduardoNtinas, GeorgiosCasteleijn, MarcoNugroho, Robertus Wahyu N.Castellari, MassimoNunes, AlexandraCastilho, Paula C.Nunes, CeciliaCastillo, JuanNunes, MartaCastillo, SandraNuñez, RosarioCastorena-Torres, FabiolaNunez-Gastélum, José AlbertoCastorina, AlessandroNunzianna, DotiCastro, AndreNural, YahyaCastro, FilipaNuţǎ, Diana CameliaCastro, José Javier FernándezNuthikattu, SaivageethiCastro, Mariana S.Nuzillard, Jean-MarcCastro, Tarsila G.Nwaiwu, OgueriCastro-Muñoz, RobertoNxumalo, WinstonCastro-Munozledo, FedericoNycz, Jacek E.Catalán, CesarNyiri, ZoltánCatani, MartinaObata, YasukoCatania, ValentinaObeidat, Belal S.Catanzaro, GiuseppinaOberlies, NicholasCatapano, MariaObrador, ElenaCatar, RusanObreza, AlešCatrouillet, SylvainObuchowski, MichalCatucci, GianlucaOcampo-García, Blanca ElíCauchy, ThomasOcampos, Fernanda Maria MarinsCaulfield, John C.Ochi, RikaCavalcanti, João Henrique F.Ochoa, JulioCavallari, Marco RobertoOcola, Esther J.Cavazza, ChristineO’Connor, MichaelCavic, MilenaOczkowski, MichałCaviglia, Gian PaoloOdagiu, AntoniaCavoski, IvanaOddo, ElisabettaCazorla-Amorós, DiegoOdegard, GregoryCea Mingueza, PilarOezkan, SeldaÇebi, NurOgueri, Kenneth S.Cebova, MartinaOh, Chang-MyungCebulak, TomaszOh, Jae-WookCelano, RitaOh, Kyung TaekCen, YanaOh, Won ChunCena, CíceroO’Hare, TimCenariu, MihaiOhashi, NamiCendrowski, AndrzejOhira, TatsuroCenobio-Galindo, Antonio De JesúsOhno, Katie M.Cepeda, JavierOhno, OsamuCerazy-Waliszewska, JoannaOhta, ShinjiCerchia, CarmenOhta, TomoeCerezo, JavierOhtsu, HiroyoshiCerna, JorgeOhtsuki, SumioČernáková, LuciaOikonomou, Evdokia K.Cerón-Carrasco, José P.Ojha, Gunendra PrasadCerrito, Maria GraziaOjovan, Michael I.Cerutti, Suzete MariaOk, Kang MinCervellati, FrancoOkada, HiroakiCesar Lemes, AiltonOkada, MotohiroCesetti, TizianaOkahashi, NobuyukiCéspedes Acuña, Carlos LeonardoOkaiyeto, KunleCetera, PaolaOkajima, TetsuyaÇetin, Arif EnginO’Keefe, RaymondCézard, ChristineOkińczyc, PiotrChabchoub, FakherOkino, NozomuChachkov, Denis V.Okuda, MasayukiChae, MichaelOkujima, TetsuoChafer, AmparoOkunade, Adewole L.Chai, Tsun-ThaiOkuniewski, AndrzejChaijan, ManatOláh, JuditChaikittisilp, WatcharopOlah, Neli KingaChakraborty, AtanuOlar, RodicaChakraborty, JoydeepOlchowik-Grabarek, EwaChałas, RenataOlczyk, PawełChamcheu, Jean ChristopherOłdziej, StanislawChampagne, Pier AlexandreOlejnik, MalgorzataChan, Kiat HwaOlejnik, MałgorzataChan, William K.Olennikov, DaniilChan, Yu-YiOlewnik-Kruszkowska, EwaChandra, JaninOliart-Ros, Rosa MaríaChandra, PreetiOlías, RaquelChandrasekaran, MurugesanOliva, JosepChang, Cheng-Wei TomOliveira Souza, AndersonChang, Chia-CheOliveira, Boaz G.Chang, Chia-ChenOliveira, CésarChang, Chi-FenOliveira, Denilson F.Chang, Chi-JungOliveira, Guedmiller S.Chang, Ching-YaoOliveira, Hugo M.Chang, Chuang-RungOliveira, J. VladimirChang, Geng-RueiOliveira, JoanaChang, Hai-ChouOliveira, MozanielChang, Hao-XunOliveira, Ricardo Pinheiro De SouzaChang, Hsueh-WeiOliveira, VitorChang, Hsun-ShuoOlivera, Ronald HuarachiChang, KunokOlivero-Verbel, JesusChang, Ling-ChuOlivieri, Alejandro C.Chang, Te-ShengOlivieri, FedericoChang, TingyuOliviero, FrancescaChang, Young-TaeOlivito, FrabrizioChang, Yu-WeiOlivo, GiorgioChao, Louis KuopingOmar, Syed HarisChaouch, Mohamed AymenOmelka, RadoslavChapman, NadineOmidian, KosarChappell, Thomas M.Omura, SeiichiCharles Richard, John LalithOñate-Garzón, JoseCharlier, DanielOnčák, MilanCharmas, BarbaraOndřej, KováříkCharpentier, ThibaultOndrias, KarolCharvet, Claude L.Onduka, ToshimitsuCharykov, Nikolay A.O’Neill, CatherineChasapis, Christos T.O’Neill, LiamChatenet, DavidOniszczuk, AnnaChatre, LaurentOnomoto, KojiChatterjee, NimratOnopiuk, AnnaChatterjee, RuchiraOntawong, AtcharapornChatzidoukas, ChristosOnys’ko, PetroChaudhry, Mohammed QasimOohora, KojiChaudhry, RasulOostenbrink, ChrisChaurand, PierreOpazo, CarlosChaurasia, BhagirathOpiela, JolantaChaurasia, SavitaOprea, ElizaChavali, MurthyOprea, OvidiuChaves, Luíse L.Opsenica, Dejan M.Chavez, Carlos A. SanhuezaOpsenica, IgorChavez, FranciscoOracz, JoannaChecinska, LiliannaOrdaz-Ortiz, José JuanCheekatla, Subba RaoÖrdög, AttilaChen, Bang-YuanOrdóñez-Díaz, José LuisChen, Bin-HueiOrekhov, PhilippChen, ChiachungOrellana-Palma, PatricioChen, Chia-YunOreshonkov, AleksandrChen, Chin-ChaoOrian, LauraChen, Chi-TienOrkusz, AgnieszkaChen, Chuan-FengOrlandini, SerenaChen, Chun-ChiOrlić, SandiChen, DaiqinOrodnez, MarioChen, DongOroian, Mircea AdrianChen, Fang-ChungOrozco, JahirChen, Fen-ErOrrù, RomanoChen, GengjunOrsi, DavideChen, GuoshengOrso, GennyChen, HaoOrtega, FranciscoChen, HongzhangOrtega-Alfaro, María CarmenChen, Hsin-ChunOrtega-Gutierrez, SilviaChen, Hsing-YinOrtega-Lara, WendyChen, Jeff Yi-fuOrtells, Marcelo OscarChen, Jian-LianOrtiz, Maria J.Chen, JiezhongOrtiz-Masiá, DoloresChen, Jing (China)Ortiz-Medina, JosueChen, Jing (UK)Ortiz-Mellet, CarmenChen, Jui-ChiehOrts, JulienChen, JunOrzelska-Górka, JolantaChen, Kuo-HuOsajima, Josy A.Chen, LechuangOsakada, KohtaroChen, LiOshiro, NaomasaChen, Liang-YuOsipova, ZinaidaChen, LingxinOsman, Ahmed I.Chen, MingzhouOsolodkin, DmitryChen, Po-WenOsorio, EdisonChen, QiOsório, Wislei R.Chen, Qiao-HongOstopovici-Halip, LilianaChen, RuiOstos Marcos, Francisco JoséChen, Shih-HengOswald, FranzChen, Shu-HuiOswald, PatrickChen, Sung-FangOta, NobutoshiChen, SuzieOtaka, JunnosukeChen, Tai-YuanOtake, HirokoChen, WeiOtero, CarolinaChen, Wei-RenOtero, MartaChen, WenmiaoOtero-Espinar, Francisco JavierChen, Xiao (China)Otmačić Ćurković, HelenaChen, Xiao (New Zealand)Ottaviano, MargaretChen, Xiao-jiaOtte, Andrew D.Chen, XinOttonelli, MassimoChen, YichengOttoni, Cristiane AngélicaChen, Yung-ChungOu, Shi YiChen, ZhonghuaOurliac-Garnier, IsabelleChen, ZhongningOuvrard, AimericCheng, Fong-YuOuyang, XiaokunCheng, Gui-GuangOves-Costales, DanielCheng, Jai-HongOwczarek, JacekCheng, Juei-tangOyama, DaiCheng, PeiOzawa, AkiraCheng, Wen-HsiOzawa, ShogoCheng, XiaopengOzawa, YoshikiCheng, XiaoqianÖzdemir, AhmetCheng, Yi-ShengOziembłowski, MaciejCheng, Yuan-BinÖzkay, YusufCheong, Kit LeongÖzvegy-Laczka, CsillaCherkaoui-Malki, MustaphaPacheco, NeithChernolovskaya, Elena L.Pacholczyk-Sienicka, BarbaraChernyshov, Ivan YuPacholska-Dudziak, EwaCheung, OceanPacia, Marta Z.Chevalier, FrançoisPaciotti, RobertoChevalier, XavierPadil, Vinod V. T.Chew, Khian-HooiPadilla-Zakour, OlgaChhetri, VijayPadjen, IvanChhikara, NavnidhiPadkina, Marina V.Chi, HaoPadnya, PavelChi, HongPadrão, JorgeChi, XiaodongPadro, Juan M.Chianese, GiuseppinaPadula, Matthew P.Chiang, Meng-TsanPaeng, KijungChiang, Wen-DeePaepegaey, Anne-CécileChiang, Ya-YuPaetz, ChristianChiarini, MarcoPagaduan, Jayson V.Chiau Ming, LongPaganin, Valdecir AntonioChiavaroli, AnnalisaPagano, AlessandraChibiryaev, AndreyPagano, BrunoChickos, James S.Pagano, CinziaChidi, Boredi SilasPagliara, StefanoChien, Hsiu-WenPagnacco, Maja C.Chierotti, Michele R.Pagnotta, StefanoChiesa, Luca M.Pagot, GioeleChillura Martino, DeliaPaizs, CsabaChin, Tan BoonPajchel, ŁukaszChin, Young-wonPajpanova, Tamara I.Chinchilla Salcedo, NuriaPajuste, KarlisChinchilla, RafaelPakravan, ParvanehChini, Maria GiovannaPal, KunalChinnathambi, ShanmugavelPalade, Laurentiu MihaiChintapalli, Sree V.Paladini, GiuseppeChiou, Chun-TangPaladino, AntonellaChiou, Tai-YingPalaniveloo, KishnethChipara, MirceaPalá-Paúl, JesúsChiper, ManuelaPalenzuela, J. AntonioChira, NicoletaPalenzuela, José AntonioChira, Nicoleta-AureliaPall, EmokeChircov, CristinaPallafacchina, GiorgiaChirgwin, John M.Pallag, AnnamáriaChirumbolo, SalvatorePallipurath, Anuradha RadhakrishnanChis, VasilePalma Sircili, MarceloChisté, Renan CamposPalma, Paulo J.Chitescu, Carmen LidiaPalma-Dibb, Regina GuenkaChiu, Hui-WenPalmer, Brian D.Chiurchiù, ValerioPalmieri, ErikaChizhov, Alexander O.Palmour, RobertaChizzola, RemigiusPalocci, CleofeChmielarz, LucjanPaloncýová, MarkétaChmielarz, PawełPalonen, PauliinaCho, Dae WonPálovics, EmeseCho, Kwang-HwiPalumaa, PeepCho, Young-HoPalumbo, OrieleChocholouš, PetrPalyulin, VladimirChoi, Chang-HoonPamies, RamonChoi, InsungPan, FeiChoi, Jae-HongPan, GuoyuChoi, Ki ChoonPan, Kuan-TingChoi, KihwanPan, Shu-YuanChoi, Kwang-YongPan, Wen-YuChoi, Myong YongPanaro, Maria AntoniettaChoi, Yong JunPande Katare, DeepshikhaChoi, YongjuPanderi, IreneChoi, Yoon-SeukPandey, PankajChoi, Young WhanPandey, PuranChojnacki, JarosławPandey, SadanandChołuj, MartaPandíțh, AnúpChorazy, SzymonPandolfi, FabianaChoupina, Altino BrancoPane, CatelloChow, James C. L.Panea, BegoñaChowdhury, PankajPanek, JaroslawChristensen, SørenPanek, RafalChristo, SusanPaneth, AgataChronakis, NikosPanfoli, IsabellaChronopoulou, EvangeliaPang, BoChruszcz-Lipska, KatarzynaPang, LisaChrysina, EvangeliaPang, ShintaroChrzanowska, AlicjaPang, YoonsooChu, SaisaiPanić, ManuelaChu, XiangpingPanighel, AnnaritaChubarov, AlexeyPanneerselvam, RajapandiyanChuck-Hernández, CristinaPanpipat, WorawanChuffa, Luiz Gustavo De AlmeidaPantić, MilicaChukhlovin, Alexei B.Panzella, LuciaChung, Ren-JeiPanzeri, DavideChung, Sheng-HengPao, Chun-WeiChunya, LinPaolella, AndreaChyau, Charng-CherngPaolesse, RobertoChylińska, MartaPaoli, PaolaCiach, AlinaPaolino, MarcoCianciulli, AntoniaPaolo, MilaniCiani, FrancescaPaolone, AnnalisaCiardelli, FrancescoPaolucci, MarinaCiardelli, GianlucaPap, JózsefCiardiello, Maria AntoniettaPapadia, ParideCiarlo, LauraPapadimitriou, Dimitra N.Ciazela, JakubPapadopoulos, Antonios N.Cicalini, IlariaPapadopoulos, GeorgiosCiccarelli, RenataPapadopoulou, LefkotheaCicchi, StefanoPapageorgiou, SpyrosCiccioli, AndreaPapakyriakou, AthanasiosCiccone, LidiaPapamichael, Emmanuel M.Ciccoritti, RobertoPapareddy, Ranjith K.Çiçek, Serhat SezaiPapavassiliou, KostasCicero, ArrigoPapierowska, EwaCicero, Calogero EdoardoPaplinska, MagdalenaCichero, ElenaPappa, AglaiaCichon, NataliaPappalardo, DanielaCichowska, JoannaPappas, ChristosCichowska-Kopczyńska, IwonaPappas, George S.Cierpiszewski, RyszardPapurello, DavideCieslik, WioletaParada-Turska, JolantaCigić, BlažPáramo-García, UlisesCikurel, HaimParastar, HadiCimas, Francisco J.Paredes, AdrianCimpoiu, ClaudiaParedes-López, OctavioCincotta, FabrizioParedes-Madrid, LeonelCinteza, OtiliaParfenova, LyudmilaCioanca, OanaParfenyuk, Elena V.Ciobotaru, Iulia CorinaParikh, PriteshCiopec, MihaelaParisotto, StefanoCipakova, IngridPark, Chan-WooCircu, ViorelPark, Deog-SuCirillo, GiuseppePark, Eun-youngCirri, DamianoPark, GyungsoonCirrincione, GirolamoPark, Jin YongCiszewski, Wojciech MichałPark, JunseongCiulla, MichelePark, Ki SooCiura, KrzesimirPark, Ki TaeCiz, MilanPark, MinCkless, KarinaPark, Min BumClaramunt, Rosa M.Park, Nae-ManClaudia, Salanță LianaPark, Seong-JikClement, CristinaPark, Seung-ChunClement, JoachimPark, Sun YoungClement, YuenParker, WilliamClemente, Miguel PaisParnetti, LucillaClift, CassandraParola, A. JorgeClos Guillen, Maria VictoriaParoul, NataliaCocan, IleanaParra, ClaudioCocovi Solberg, David JaimeParra, MargaritaCodina, GeorgianaParra‐Delgado, HortensiaCoelho, Marsen Garcia PintoParrino, BarbaraCoïsson, Jean DanielParrot, DelphineCojean, SandrineParsons, JasonCojocaru, CorneliuPartyka, AnnaColacio, EnriqueParvez, KhaledColas, CyrilPascal, DievartColdea, Teodora EmiliaPascali, GiancarloColecchia, Salvatore AntonioPaschoal, DiegoColella, MarcoPashkin, OleksiyColetta, AntonioPasini, DarioColetti, CeciliaPasini, MariaceciliaColl, DeysmaPasquini, SilviaColla, Luciane MariaPassero, Luiz Felipe DominguesCollado, AidaPassini, ElisaCollado, DanielPastene-Navarrete, Edgar R.Collins, Stuart G.Pašti, Igor A.Colmati, FlávioPasti, LuisaColnago, Luiz A.Pastor, Isidro M.Colom, XavierPastor, NinaColombo Pimentel, TatianaPastuch-Gawolek, GabrielaColombo, GiorgioPaszkiewicz, SandraColombo, Sara FrancescaPatel, DharmeshkumarColomina, Maria TeresaPatel, Kapil D.Colone, MarisaPatel, KrutikaČolović, Mirjana B.Paterová, PavlaColuccia, AntonioPathuri, GopalColunga Biancatelli, Ruben Manuel LucianoPati, SiddharthaComan, PaulPatil, Santosh S.Comanescu, CezarPatra, BishnubrataComas-Garcia, MauricioPatrizi, BarbaraCombarros, PatriciaPatruno, AntoniaComini, Laura RaquelPatterson, JenniferComis, Alessio DaniloPattison, GrahamComito, Robert J.Paudel, AtmikaConcilio, SimonaPaudel, Keshav RajConde, EloyPauk, VolodymyrCondelli, NicolaPaul, FredericConde-Mejía, CarolinaPaul, SanjoyConradt, ReinhardPaul, UttamConsalvi, SaraPaulino, JoanaConsole, LaraPauss, AndreConstantin, Oana EmiliaPautz, AndreaConstantinescu-Aruxandei, DianaPavao, Mauro SergioConstantinides, ChristosPavela, RomanConsumi, MarcoPavic, KristinaConte, CarmelaPavkov, IvanConti, ClaudiaPavlić, BranimirConti, PaoloPavlos, KlepetsanisContiero, JonasPavlović, NebojsaContin, MarcoPavlovic, RadmilaCoombs, MelaniePawar, NitinCoons, James E.Pawar, ShrikantCopolovic, Dana MariaPawełczyk, AnnaCopolovici, LucianPawelec, BarbaraCoppola, DanielaPawlak, AndrzejCordaro, MarikaPawlak, KatarzynaCordes, TheklaPawłowska, BarbaraCordoba-Diaz, DamianPawlowski, KatharinaCornett, ClausPazourek, JiříCorrêa, Arlene GonçalvesPearce, JohnCorreia, Ana CristinaPearce, NorrieCorreia, IsabelPéč, MartinCorreia, ManuelaPedatella, SilvanaCorreia, Sónia C.Peddapuram, AdithyaCortés, Farid B.Pedone, DeborahCortés, FernandoPedraza Chaverri, JoséCortés-Benítez, FranciscoPeixoto, Maria JoãoCortiella, Mariona GilPejchal, JaroslavCortina, Jose LuisPejin, BorisCorvis, YohannPeláez, RafaelCosano, DanielPelin, CristinaCoscueta, EzequielPeltzer, MercedesCosme, FernandaPeluso, IlariaCosta De Camargo, AdrianoPeña Fernández, María ÁngelesCosta, EduardoPeña Neira, Alvaro IvánCosta, GuyPeña, JavierCosta, Maria Do CéuPeña, Jose IgnacioCosta, StefaniaPeña-Ramos, Etna AídaCosta-Almeida, RaquelPenaranda-Foix, Felipe L.Costabile, AdelePeng, HaoCosta-de-Oliveira, SofiaPeng, Shu-FenCostales, AuroraPeng, Wen-HuangCosta-Nunes, João PedroPeng, XiaoCosta-Pinto, Ana RitaPeng, Xiao-ShuiCostinel, DianaPengo, PaoloCostisor, OtiliaPenha, Frederico MarquesCotârleț, MihaelaPennisi, RosamariaCotea, Valeriu V.Penteado, FilipeCoudert, JeromePeragón, JuanCoutrot, FrédéricPeral, AngelCovington, JamesPercário, SandroCox, JamesPerdih, AndrejCozza, GiorgioPereira, ClaudiaCozzolino, DanielPereira, ElianaCozzolino, FloraPereira, Fernando J.Cozzolino, RosariaPereira, LeonelCravotto, GiancarloPereira, MatheusCrawford, Philip W.Pereira, Renato B.Crespi, StefanoPereira-Maia, EleneCretoiu, DragosPerera, LalithCreutz, SidneyPerera, WilmerCrisan, LuminitaPerestrelo, RosaCrisp, PeterPerestrelo, Rosa Maria De SáCrisp, Ryan W.Perez Castillo, YunierkisCrispi, StefaniaPérez De Vega, María JesúsCrispini, AlessandraPerez Del Pino, AngelCristea, CasteliaPerez, SergeCristóvão, BeataPérez-Alvarez, Jose AngelCrnkovic, AnaPérez-Burillo, SergioCroce, Anna CletaPérez-Casas, SilviaCroci, DiegoPerez-Castineira, JoseCrook, NathanPérez-Cataluña, AlbaCrulhas, Bruno PereiraPérez-Gago, María BernarditaCruz, AndreaPerez-Gregorio, RosaCruz, Homero Reyes De LaPérez-Lamela, ConcepciónCruz, Rodrigo Alves SoaresPérez-Lozano, PilarCruz, Rui M. S.Pérez-Rojas, Jazmin M.Cruz, TiagoPérez-Santos, MartínCruz-Sosa, FranciscoPérez-Vázquez, VictorianoCsávás, MagdolnaPérez-Villanueva, JaimeCsuk, RenéPerfetti, MauroCuadrado, CarmenPergher, Sibele B. C.Cuadrado, IrenePergolizzi, SimonaCubilla-Rios, LuisPerinelli, Diego RomanoCuccovia, Iolanda MideaPerini, MatteoCuffaro, DorettaPerkins-Veazie, PenelopeCui, DongmeiPerkowski, PawelCui, HaiyangPerlepes, Spyros P.Cui, JingnanPerles, JosefinaCui, QingbinPerna, GiuseppeCui, QingyuPero, Maria ElenaCuisset, ArnaudPerpetuini, GiorgiaCukrov, NevenPerreau, FrancoisCulbert, JuliePerri, AnnaCummins, AdrianPerrin, LaraCundari, Thomas R.Perrino, Enrico VitoCuoco, MarioPerrotta, CristianaCurchod, Basile F. E.Perry, John D.Curtis, MarionPeršurić, ŽeljkaCurulli, AntonellaPerucka, IrenaCurutiu, CarmenPeruzynska, MagdalenaCvejić, ŽeljkaPeruzzini, MaurizioCycoń, MariuszPervakov, KirillCypryk, MarekPescetelli, SaraCysewski, PiotrPeška, VratislavCzachor, MarekPessela João, BenevidesCzapik, AgnieszkaPessler, FrankCzaplewska, PaulinaPessoa, Rodrigo SávioCzaplicki, SylwesterPeter Amalathas, AmalrajCzarniecka-Skubina, EwaPeter, MartinCzarnota, RobertPeters, ThomasCzech, BożenaPetersmann, AstridCzechowski, TomaszPetkova, NadezhdaCzekala, WojciechPetkovic, MilenaCzekelius, ConstantinPetr, JanCzemierska, MagdalenaPetrak, JiriCzernek, JiříPetrescu, EmilCzerwińska, MonikaPetretto, GiacomoCzerwiński, MarcinPetri, Uusi-KyynyCzop, MarcinPetrignet, M. JulienCzyrski, AndrzejPetrillo, GiovanniCzyz, Daniel M.Petronilho, AnaCzyż, KatarzynaPetropoulos, SpyridonCzyżowska, AgataPetrotos, KonstantinosĐ. Glišić, BiljanaPetrov, DmitryD. Škrbić, BiljanaPetrović, Jelena T.Da Cunha, Mário Antônio AlvesPetrović, PredragDa Hora, Gabriel Costa A.Petrucci, RobertoDa Rosa, Marcelo BarcellosPetruczynik, AnnaDa Silva Pena, RosinelsonPetti, CarloalbertoDa Silva Souza, Ivan DomicioPetzold, GuillermoDa Silva, João FerreiraPeuchmaur, MarineDa Silva, José P.Peuronen, AnssiDa Silva, Luís C. N.Peveler, WilliamDa Silva, Luis Cláudio NascimentoPeyrot, FabienneDa Silva, Luís PintoPezda, JacekDa Silveira Petruci, João FlávioPezzella, CinziaDa Veiga Junior, Valdir FlorencioPhadtare, VarshaDąbrowska, MonikaPham, Hong Ngoc ThuyDa̧browski, MichałPham, Tien DucDacarro, GiacomoPhan, Chin-SoonDadáková, KateřinaPhapale, Prasad B.Daferera, DimitraPhechkrajang, ChutimaDagnon, SoleyaPhilippe, AllanDagrosa, María AlejandraPhilipsen, HaroldDai, YuminPhillips, David LeeDai, ZhenghongPhillips, DonDal Prá, IlariaPi, RongbiaoDalgarno, ScottPi, TeresaDallavalle, SabrinaPiao, XijunDaloso, Danilo M.Piastowska-Ciesielska, AgnieszkaDama, MuraliPiątczak, EwelinaDamalanka, VishnuPiątkowska, EwaDamaziak, KrzysztofPiccialli, GennaroDamian, Celina MariaPiccialli, IlariaDamkaci, FehmiPicerno, PatriziaDams-Kozlowska, HannaPichler, AnitaDa’na, EnshirahPicimbon, Jean-FrançoisDanac, RamonaPicinelli-Lobo, AnnaDanaher, MartinPico, YolandaDanalev, DanchoPieczonka, Adam M.Dandlen, Susana AnahiPiegza, MichałD’andrea, CristianoPiekoszewski, WojciechD’Andrea, Luca DomenicoPielichowski, KrzysztofDanel, Krzysztof S.Pieper, JörgDanesi, FrancescaPierpaoli, MattiaDang, JunPierson-Wickmann, Anne-CatherineD’Angelo, StefaniaPiesik, DariuszDaniel-da-Silva, Ana LuísaPiestansky, JurajDaniele, AuroraPietropaolo, AdrianaDanielski, RenanPietropolli Charmet, AndreaDanielson, Neil D.Pietrusiewicz, Kazimierz MichałDanila, OctavianPietrzak, AnnaDanilović, Bojana R.Pietrzak, MarekDanko, MartinPietrzak, WioletaDante, SilviaPietrzkowski, ZbigniewDántola, María LauraPiffoux, MaxDarabantu, MirceaPigulski, BartłomiejDarbre, TamisPijning, TjaardD’Archivio, Angelo AntonioPike, Andrew RobertDarenskaya, Marina AleksandrovnaPilan, LuisaDarie-Nita, Raluca NicoletaPilatus, UlrichDas, ViswanathPilic, BrankaDasgupta, SrimoyeePilicheva, BisseraDashti, YousefPilkington, LisaDaszykowski, MichałPilla, VivianeDátka, JerzyPilmane, MāraDatta, SandipanPiluzza, GiannellaDatta, ShubhashisPimentel, CarlosD’Auria, Maria ValeriaPimerzin, AlekseyDávalos, Juán Z.Pimienta, Rodney LacretDavank, VadimPina, MariaDavid, Gabriela IuliaPinakoulaki, EftychiaDavid, Robert OscarPinart, ElisabethDavid, VictorPindelska, EdytaDavidchack, Ruslan L.Pinela, JoséDavídková, MariePiñero, ManuelDavies, CarolinaPinheiro, JoaquinaDávila López, MarcelaPinheiro, José PauloDavis, AlexanderPinho, Ana CatarinaDavis, David A.Pinho, LuísDavletov, AskarPini, ElenaDay, Chi-PingPinilla-Cienfuegos, ElenaDay, PriscillaPinteala, MarianaDayal, NeetuPinto Da Silva, LuísDe Almeida, EdgarPinto, Diana CláudiaDe Almeida, José RafaelPinto, ErnaniDe Alvarenga, Elson SantiagoPinto, LorisDe Amici, MarcoPinto, SaraDe Araujo, ElvinPintus, GianfrancoDe Barros Fernandes, PedroPinu, Farhana R.De Bellis, LuigiPinzi, LucaDe Boni, LeonardoPiosik, JacekDe Caprariis, BenedettaPiotr, BugajskiDe Castro Figueiredo Bordon, KarlaPiotrowicz-Cieślak, AgnieszkaDe Castro Ramalho, TeodoricoPiotrowska, DorotaDe Cerain, Adela LopezPipino, CaterinaDe Chiara, Maria Lucia ValeriaPipiška, MartinDe Curtis, FilippoPiragine, EugeniaDe Falco, BrunaPiras, SandraDe Figueiredo, Eduardo CostaPires, Regina HelenaDe Figueiredo, MarciaPires, Ricardo HugoDe Filippis, BarbaraPirkmajer, SergejDe Haan, NoortjePirmettis, IoannisDe Hoyos Martínez, Pedro LuisPiro, BenoîtDe Jesús Ruíz-Baltazar, ÁlvaroPirota, ValentinaDe La Lastra, José Manuel PérezPisano, Pablo L.De La Parte, Borja HerreroPisarcik, MartinDe Lázaro, Sérgio RicardoPiscopo, AmaliaDe Lima Nobre, Marcos AugustoPiscopo, MarinaDe Los Rios, CristobalPisk, JanaDe Luca, StefaniaPisklak, Dariusz MaciejDe Lurdes Dapkevicius, MariaPiskorz, WitoldDe Marco, IolandaPiskunov, AlexandrDe Marino, SimonaPistelli, LauraDe Martin, RainerPistorio, ValeriaDe Masi, GianlucaPitschmann, VladimirDe Miranda, João Antônio LealPitsikas, NikolaosDe Mol, MaartenPitucha, MonikaDe Oliveira, Ariel ColaçoPivetta, TizianaDe Oliveira, Ivan PiresPiwowarek, KamilDe Oliveira, José Miguel P. FerreiraPizzuti, LucasDe Oliveira, Kléber T.Placido, JerssonDe Oliviera Silva, ElianePlaczek, William J.De Paola, IvanPlanque, ChrisDe Pascual-Teresa, SoniaPlastina, PierluigiDe Pauw, Edwin A.Plata, Jose J.De Paz, Antonio TaberneroPlatella, ChiaraDe Petris, LuigiPlazinski, WojciechDe Queiroz, Vagner TebaldiPlech, TomaszDe Rosa, LuciaPleil, Joachim DieterDe Rosa, Maria CristinaPlenis, AlinaDe Simone, Salvatore GiovanniPliss, Evgenii M.De Souza Gil, EricPlochberger, BirgitDe Souza, CarolinaPłotka-Wasylka, JustynaDe Souza, Gustavo Henrique BiancoPluteanu, FlorentinaDe Vendittis, EmmanuelePlyusnin, Victor F.De Villiers, MelgardtPoapolathep, AmnartDe Vita, DanielaPobiega, KatarzynaDe Wergifosse, MarcPochard, IsabelleDe Zorzi, RitaPoddar, SoumyaDe Zotti, MartaPoddelsky, AndreyDeacon, GlenPodgornov, Fedor V.Debabrat, BaishyaPodlacha, MagdalenaDębowski, MarcinPodleśny, JanuszDec, JanPodlewska, SabinaDeed, Rebecca C.Podlipnik, ČrtomirDefant, AndreaPodolak, IrmaDefrancq, EricPodsiadły, RadosławDegl’innocenti, DonatellaPodsiadły-Paszkowska, AgataDel Angel, Rosa MaríaPogacnik, LeaDel Castillo-Santaella, TeresaPoggini, LorenzoDel Gatto, AnnaritaPogorzelska, AnetaDel Porto, PaolaPogrmic‐Majkic, KristinaDelegou, Ekaterini T.Pohl, RadekDelgado, Daniel RicardoPohlkamp, TheresaDelgado, Sergio NogalesPöhlmann, ChristopherDeligiannakis, YiannisPohlmeier, AndreasDeligiannidou, Georgia-EiriniPoiana, Mariana-AtenaDelivopoulos, EvangelosPoisot, Martha E.Della Malva, AntonellaPoitevin, Carolina G.Della Sala, FabioPolák, BeataDella Sala, PaoloPolak, TomažDellacassa, EduardoPolčic, PeterDellafiora, LucaPoleshchuk, Oleg Kh.Dellis, SpiliosPolewski, KrzysztofDeLouise, Lisa A.Polishchuk, PavelDelsuc, Marc-AndréPolito, LetiziaDemarquoy, JeanPolizzi, ElisabettaDembska, Anna RenataPolonski, TadeuszDeme, PragneyaPolovic, NatalijaDemerdash, Omar N. A.Polz-Dacewicz, MałgorzataDemetrescu, IoanaPoma, AdolfoDemina, TatianaPoma, AlessandroDemirci, FatihPomari, ElenaDemirci, HasanPomerantz, WilliamDemopoulos, Vassilis J.Pomierny-Chamioło, LucynaDeng, HaitengPomorski, AdamDeng, Ming JayPonce-Alquicira, EdithDeng, QiliangPonce-Vargas, MiguelDenisenko, Yuriy G.Ponczek, Michał BłazejDenisow, BozenaPonder, AlicjaDenorme, FrederikPongtip, SithisarnDenton, AlisandraPonnambalam, VasDeochand, DineshPonti, AlessandroDe Oliveira, Ronaldo NascimentoPook, ChrisDepeint, FlorePoór, MiklósDeptuch, AleksandraPop, Laura AncuţaDeracinois, BarbaraPop, Lecturer Carmen RodicaDerchi, GiacomoPop, Raluca MariaDerewiaka, DorotaPoparić, GoranDerita, Marcos G.Popescu, Mihaela R.Derkowska-Zielinska, BeataPopescu, Paul-AlexandruDeRosa, MariaPopescu, Roxana CristinaD’Errico, StefanoPopescu, Sanda MihaelaDeryabina, Yu I.Popescu, VasilicaDesiderio, ClaudiaPoplawski, TomaszDesislava, TenevaPopović Đorđević, JelenaDesmaële, DidierPopovic, BorisDesmet, TomPorat, Ze’evDesogus, FrancescoPorcari, Andréia De MeloDeterman, JohnPorcayo-Calderon, JesusDetsi, AnastasiaPorro, BenedettaDettmer, UlfPorro, ChiaraDettori, Maria AntoniettaPorta, HelenaDev, SomPortier, ChristophDevkota, Hari PrasadPortilla, JaimeDhakal, HomPortugal, Camila C.Dhakal, PratikPorzel, AndreaDhakal, SomaPoŝa, MihaljDhaliwal, Gurjot S.Pospisil, JiriDhasarathy, ArchanaPospisil, TomasDhiman, MahakPossan, EdnaDhokale, RanjeetPotaczek, Daniel P.Di Bella, GiuseppaPotaniec, BartłomiejDi Fiore, AnnaPotapov, Vladimir A.Di Foggia, MichelePothula, SantoshDi Francesco, AlessandraPotoroko, IrinaDi Giacomo, SilviaPoudel, Tej NarayanDi Giacomo, VivianaPouli, NicoleDi Gilio, AlessiaPoulin, RemingtonDi Gioia, SantePoupot, RemyDi Giosia, MatteoPourbaghi, MiladDi Girolamo, RoccoPourquier, PhilippeDi Liberto, ValentinaPozdnyakov, Aleksandr S.Di Lorenzo, FrancescoPozharitskaya, OlgaDi Maro, AntimoPoznański, JarosławDi Martino, AntonioPozzi, AndreaDi Pierro, ProsperoPozzi, CeciliaDi Pietro, Maria EnricaPozzi, EleonoraDi Renzo, FrancescoPradhan, Dhiren K.Di Salle, AnnaPradotto, Luca GuglielmoDi Sanzo, RosaPramanik, BapanDi Sotto, AntonellaPrasad, SahdeoDi, LeiPrasadh, SomasundaramDi, XiaxiaPratesi, AlessandroDiaconu, Carmen CristinaPratsinis, HarrisDiana, RositaPraznik, WernerDias, Clarice NoletoPrazuch, JanuszDias, Fábio De SouzaPreda, NicoletaDias, Fernanda F. G.Premakumara, Galbada Arachchige SirimalDias, FernandoPreti, DeliaDias, Maria CelestePreto, JordaneDias, Maria Isabel RibeiroPrévost, SébastienDias, NicolinaPriego, Eva-MariaDiaz Pace, DiegoPrieto Dapena, FranciscoDiaz, GasparPrieto, CristinaDíaz-Castillo, FredycPrieto, Jose M.Díaz-García, DianaPrieto, Miguel ÁngelDiaz-Marrero, Ana R.Primas, NicolasDíaz-Molina, RaúlPrinsloo, GerhardDiaz-Oltra, SantiagoPriola, EmanueleDichiara, AnthonyPrior, Timothy J.Dietz, ChristianPriprem, AroonsriDiez, Emiliano RaúlPrivette Vinnedge, LisaDifonzo, GrazianaPrivitera, AlbertoDigiacomo, GrazianaPriyadarshi, RuchirDiliën, HannePriye, AashishDima, Stefan-OvidiuPrkić, AnteDimakopoulos, YannisProbert, ChrisDimas, KonstantinosProcházková, EliškaDimcheva, VanyaProença, CarinaDimić, DušanProença, LuísDimitrov, IvayloProsen, HelenaDimmock, Jonathan R.Proszkowiec-Weglarz, MonikaDimos, KonstantinosProto, AndreaDincheva, IvaylaProtti, MicheleDinda, BiswanathProux, OlivierDing, Bao-JianPrugovečki, BiserkaDing, JianxunPrzybyłek, MaciejDing, ShangwuPrzybylek, PiotrDing, XinhuaPsarras, Antonios C.Ding, YunjiPtaszek, MarcinDinic, JelenaPtaszek, PawełDinić, SvetlanaPtaszynska, AnetaDinică, RodicaPu, LumeiDinica, Rodica-MihaelaPuac, NevenaDinman, JohnatanPucci, MarziaDinos, GeorgiosPucci, NicolettaDiogo, Hermínio P.Pucelik, BarbaraDionisio, GiuseppePuchalka, RadoslawDippong, ThomasPuchalski, CzesławDiskin-Posner, YaelPuchart, VladimírDitaranto, NicolettaPuertas‐Mejía, Miguel A.Ditta, AllahPugajeva, IvetaDiuzheva, AlinaPuglisi, AlessandraDivis, PavelPuitel, Adrian CǎtǎlinDivol, BenoitPuizina, JasnaDjakovitch, LaurentPujals, SílviaDjedaïni-Pilard, FlorencePukalskas, AudriusDjuragic, OliveraPulavarti, Surya V. S. R. K.Djuran, Miloš I.Purgel, MihályDludla, Phiwayinkosi V.Purkrtová, SabinaDługaszewska, JolantaPușcaș, AndreeaDo Lago, Bárbara VieiraPüschel, Gerhard P.Doan, HuanPusey, Marc L.Dobrovolna, JanaPutra, Masteria YunovilsaDobrzynska, MalgorzataPutrinš, MartaDobrzyńska-Inger, AgnieszkaPutt, KarsonDobrzynski, PiotrPuttisong, YuttapoomDocoslis, ArisPyataev, Nikolay A.Doda, Sai ReddyPycia, KarolinaDodero, AndreaPyka, AlinaDoerksen, Robert J.Qasim, MuhammadDoganis, PhilipQhairul, NorDogkas, GeorgeQi, HangDogossy, GáborQin, LiguoDohanosova, JanaQin, XiaoliDolega, AnnaQin, ZhaohaiDolfing, JanQing, LinsenDolganov, Pavel VladimirovichQiu, BinDoligalska, MariaQneibi, MohammadDolle, MickaelQu, KeDołowy, MałgorzataQu, XiaozhongDolzhenko, Anton V.Quadrelli, PaoloDomagała, MałgorzataQuagliariello, VincenzoDomalik-Pyzik, PatrycjaQuan, Nguyen VanDoménech-Carbó, AntonioQuang Le, BachDomian, EwaQuesada, ErnestoDomin, HelenaQuevedo, Ivan R.Domingues, EvaQuijada-Maldonado, EstebanDomingues, Valentina F.Quijano, LeovigildoDomínguez Álvarez, EnriqueQuilez, JoseDominguez Vías, GermanQuílez-Bermejo, JavierDominguez-Martin, AliciaQuinn, Mark T.Dominguez-Robles, JuanQuiñonero, DavidDomínguez-Soberanes, JulietaQuintas, Luis Eduardo M.Domínguez-Vías, GermánQuiroga, DiegoDominikowska, JustynaRaasakka, ArneDonadu, MatthewRabezzana, RobertoDoncheva, Tsvetelina E.Rabiei, AminDong, FengpingRacovita, Radu C.Dong, HaiRacovita, StefaniaDong, HuaRaczyk, MariannaDong, XiaoboRaczyńska, EwaDong, XinyueRadan, MilaDong, YiRadchenko, Eugene V.Dong, YueshengRadenković, SlavkoDoni, Mihaela BadeaRadisavljevic, ZivDonnermeyer, DavidRadon, MariuszDoris, EricRadosavljević, TatjanaDoron, RamyRadotic, KsenijaDoroshenko, Andrey O.Radtke, ValentinDorożyński, PrzemysławRadu, BeatriceDos Santos Lima, MarcosRadu, Gabriel LucianDos Santos, CatarinaRaducan, AdinaDos Santos, Demetrio JacksonRadulescu, CristianaDos Santos, Jean LeandroRadušienė, JolitaDos Santos, José Cleiton SousaRadwan, Mohamed OsmanDosio, FrancoRafael Jesus Gonçalves, RubiraDotsikas, YannisRafael, Del Rio-SalasDou, JinzeRafi, MohamadDoucet, MathieuRafique, JamalDoulberis, MichaelRaggi, CarlaDouma, MountasserRago, DanielaDourtoglou, VassilisRagusa, AndreaDoustkhah Heragh, EsmaeilRagusa, SalvatoreDoutch, JamesRahim, Muhammad ImranDouvas, AntoniosRahimi, FaridDouvris, ChrisRahman, A. F. M. MotiurDovinova, ImaRahman, AsadurDoxakis, EpaminondasRahman, Khondaker MirazDoytchinova, IriniRahman, M. AzizurDrabik, AnnaRahman, Md AtiarDrabińska, NataliaRahman, Md. HabiburDrabowicz, JózefRahman, Md ToufiqurDragan, Ecaterina StelaRahman, Mohammad MizanurDrăgoi, Cristina ManuelaRai, RewaDragonieri, SilvanoRai, VineetaDrašar, Pavel B.Railean-Plugaru, VioricaDreassi, ElenaRaimondi, Maria ValeriaDreger, MariolaRaj, DanutaDrenkova-Tuhtan, AsyaRaja, RobertDrew, MikeRajauria, GauravDrewry, DavidRajčević, NemanjaDreyse, PaulinaRajić, ZrinkaDrgan, ViktorRajkovic, IvanDrijfhout, FalkoRajnarayanan, RajendramDrochioiu, GabiRajoka, Muhammad Shahid RiazDrozd, ArletaRakic, VesnaDryahina, KseniyaRalston, EmilyDu, HaifengRam Paudel, MuktiDu, HaishunRamadan, SherifDu, LinRamalho, Maria JoãoDu, LipingRamalho, Teodorico C.Du, XiaoboRamanauskienė, KristinaDu, XinRamanavicius, ArunasDu, XushengRambabu, KrishnamoorthyDu, ZhifengRambla, José L.Duan, PuRamesh, KalyanDuan, WeiRamírez, DavidDuan, YanRamirez-Castrillon, MauricioDuarte, BernardoRamírez-Jiménez, AureaDuarte, NoéliaRammohan, AluruDuarte-Correa, YudyRamona Moise, AdelaDuatti, AdrianoRamos, AnaDubey, NileshkumarRamos, CarmenDubey, SushilRamos, JavierDublan-García, OctavioRamos, M. TeresaDubnika, AritaRamos, Patricia A. B.Dubrova, YuriRamos, PatricioDucreux, SylvieRamos, Ricardo MartinsDuda-Chodak, AleksandraRamos-Organillo, ÁngelDuda-Madej, AnnaRamos-Ortiz, GabrielDudhipala, NarendarRamsbeck, DanielDudley, GregoryRanalli, GiancarloDufossé, LaurentRangelov, StanislavDughera, StefanoRanieri, AnnamariaDuh, Pin-DerRanitović, AleksandraDukarska, DorotaRanjit, SumanDulf, FranciscRao, AshitDumancas, GerardRao, YulanDumitrascu, LoredanaRâpeanu, GabrielaDumitriu, Georgiana-DianaRaposo, MariaDunkić, ValerijaRapson, Trevor D.Duong, Thuc HuyRascon, AgustinDurairaj, RajeshRašeta, MilenaDurante, MirianaRashidieh, BehnamDuranti, GuglielmoRaška, MilanDurán-Valsero, Juan JoséRasoga, OanaDurgo, KsenijaRasool, NasirDurka, KrzysztofRaspor, MartinD’Ursi, Anna MariaRassias, GerasimosDushkin, Alexander Valer’evichRastija, VesnaDuta, Denisa E.Rataboul, FranckDutartre, PatrickRatautaite, VilmaDutkiewicz, MariolaRateb, MostafaDutkiewicz, ZbigniewRather, GulamDutta, AdityaRathinasabapathy, AnandharajanDutu, LigiaRathke, BerndDvornic, Petar R.Rathore, Atul SinghDyakonov, VladimirRatiu, AndreeaDymerski, TomaszRatusz, KatarzynaDžamić, AnaRaugei, GiovanniDželetović, ŽeljkoRauk, ArviDzhambazov, BalikRaúl, Avila-SosaDziadas, Mariusz TomaszRault, FrancoisDziki, DariuszRauseo, JasminDziomba, SzymonRavagnan, GiampietroDziurka, DorotaRavallec, RozennDzolic, ZoranRavanidis, StylianosDzugan, MalgorzataRavi, Rama S.Eamens, Andrew L.Ravindranadh, KoutavarapuEar, JasonRawat, SwatiEbadollahi, AsgarRawson, JeremyEbeed, HebaRay, DurbarEbensperger, GermánRay, SupriyoEbrahimi, SamadRayas-durte, PatriciaEchevarria, AureaRaymundo-Pereira, Paulo A.Echeverri, FernandoRayson, Gary D.Eder, MatthiasRaza, Md KausarEder, ReinhardRazak, SuhailEdin, Nina JeppesenRazgonova, MayyaEdiriweera, Meran KeshawaRazo, RodrigoEdison, T. Jebakumar ImmanuelRe, NazzarenoEdith Rojas, AnayaRe, SuyongEdrees, Mastoura MohamedRead, Roger W.Eduardo-Figueira, MariaReal Oliveira, Maria Elisabete C. D.Edwards, Adam B.Rébé, CédricEedara, Basanth BabuReber, Eleanora A.Eevers, WalterRecabarren Gajardo, GonzaloEfimov, AlexanderReddy, Sudhir PuttyEfremenkova, Olga V.Reddy, Y. Veera ManoharaEgawa, ToruRedón, RocíoEgberts, PhilipRedondo, Pedro CosmeEgorin, AndreiReeves, Helen L.Egorova, KseniaRegel-Rosocka, MagdalenaEhm, LarsReggelin, MichaelEhman, Nanci VanesaRegier, MarcEickerling, GeorgRégnier-Vigouroux, AnneEid, NabilReichenwallner, JörgEimutis, JuzeliūnasReichert, David E.Eiroa, José LuisReid, Michael S.Ejfler, JolantaReid, ScottEl Aouad, NoureddineReiff, TobiasEl Bakri, YounessReinhardt, ChristophEl Moutaouakil, AminReinmöller, MarkusElad, DanielReinoso, SantiagoEl-Ansary, HosamReinscheid, Rainer K.Elaziz, Mohamed AbdReis, HeribertEl-Bacha, TatianaReis, Sara S.Eldridge, SandyReitzel, AdamElena, EnachiRej, SouravEl-Far, Ali H.Rejah, ShahiElgamel, AliRejinold, SanojEl-Gendy, Ahmed A.Remedio, RafaelEl-Huneidi, WaseemRen, DonglouElías-Zúñiga, AlexRen, QinlongEliav, EphraimRen, ZhiweiElKamhaw, AhmedRenaud, JustinElKelish, AmrRenault, JacquesEller, Cleiton BrederRendueles, ManuelEllert-Miklaszewska, AleksandraRengifo, Julián AndrésElliott, Paul I. P.Renzo, VannaElmoselhi, AdelRepetto, Marisa GabrielaEl-Newehy, MohamedReščič, JurijEl-Sayed, Nesrine Salah DineResztak, MatyldaEl-Sayed, Wael A.Retamal, MauricioElshamy, AbdelsamedRety, StephaneElvira-Torales, Laura InésReveglia, PierluigiElwakeel, KhalidReverchon, ErnestoElyashberg, MikhailReyes, EfraímEmilia, DrozlowskaReyes, Luis H.Emmanouil, ChristinaReyes-Ortega, FelisaEmmert, Luke A.Reynisson, JóhannesEncinar, Jorge RuizRey-Raap, NataliaEncina-Zelada, Christian R.Řezáčová, MartinaEndo, MakotoRial, CarlosEndoh, TamakiRial-Hermida, IsabelEnkvist, ErkiRiassetto, DavidEnriquez, Raul G.Riazuddin, SheikhEnserink, Jorrit M.Ribaudo, GiovanniEo, Soo HyungRibeiro, ArturEramo, StefanoRibeiro, ClaudiaEras, JordiRibeiro, José A.Erben, MauricioRibeiro, Luís FilipeEremin, DmitryRiboni, NicolòErfani, MostafaRicardo, GobatoEritja, RamonRiccardi, ClaudiaErland, LaurenRicchi, MatteoErnesto-Casanova, OscarRiccio, RaffaeleEroglu, AbdulkerimRicelli, AlessandraEsatbeyolgu, TubaRichert, MariaEscalante, JaimeRichtera, LukášEscárcega Bobadilla, Martha VerónicaRico-Yuste, AlbertoEscargueil, AlexandreRiddick, Eric WellingtonEscobedo-Avellaneda, ZamanthaRieder, ElizabethEscorihuela, JorgeRigalli, Juan PabloEscott, CarlosRigillo, GiovannaEscudero, OlgaRimoldi, IsabellaEseberri, ItziarRimondini, LiaEskiköy Bayraktepe, DilekRinaldi, RobertoEskin, MichaelRios Solis, LeonardoEspinach, Francesc XavierRios-Estepa, RigobertoEspinosa Escalona, Berta AlejandraRíos-Gutiérrez, MarEspinosa-Aguirre, JavierRistori, SandraEspinosa-Reyes, GuillermoRivadeneyra, AlmudenaEspinoza-Vázquez, AraceliRivas, Mariella O.Esposito, Fortunato PalmaRivas-Galindo, Verónica MayelaEsposito, VeronicaRiveira, Martín J.Espuelas, SocorroRiziotis, ChristosEsquerra, RaymondRizk, Mohamed AbdoEsquivel, BaldomeroRizvi, Syed A. A.Esteve Ràfols, MontserratRizzo, CarmenEstevez, Juan C.Rizzo, MilenaEsti, MarcoRizzo, ParideEstrada-Gutierrez, GuadalupeRizzuti, BrunoEstrada-Tejedor, RogerRoberts, TaraEstrada-Zúñiga, María ElenaRobertson, Mark J.Estrany, FrancescRobichaud, DavidEtheve-Quelquejeu, MélanieRobien, WolfgangEtienne, MathieuRobin, MaximeEtinski, MihajloRobinson, Jerome R.Ettayapuram Ramaprasad, Azhagiya SingamRobledo Quintos, Norma REudald, CasalsRobles, Eva SánchezEusébio, Maria Ermelinda S.Roca, SunčicaEvangelisti, LucaRoca-Sanjuán, DanielEvens, AnneRocca, RobertaEwa, SkwarekRocchetti, GabrieleEwen, JamesRocco, LuciaEymar, EnriqueRocha, CláudiaFabiola, Castorena-TorresRocha, HugoFabiszewska, AgataRocha, SilviaFabre, BrunoRocha, Vinícius P. C.Fabroni, SimonaRochat, BertrandFacchetti, GiorgioRochfort, KeithFadda, AngelaRockenbauer, AntalFahey, JedRockenfeller, PatrickFahmi, AmirRode, Joanna E.Fakis, MihalisRodrigo, LuisFalabella, EduardoRodrigues, AliceFalacho, RuiRodrigues, FranciscaFalanga, AnnaritaRodrigues, Márcio José De AbreuFali, TinhinaneRodrigues, Marili Villa NovaFalsini, SaraRodríguez Alfonso, Armando A.Fameau, Anne-LaureRodríguez Díaz, Juan CarlosFan, RuiqingRodríguez González, VicenteFan, YuxiaRodríguez López, María IsabelFandino, OliviaRodriguez Rojo, SorayaFang, KegongRodríguez Romero, AdelaFang, MingchihRodríguez Villanueva, JavierFanizzi, Francesco PaoloRodríguez, JaimeFantoni, RobertaRodríguez, José AntonioFarag, Mayada RagabRodríguez, MartínFaraone, ImmacolataRodriguez, NoelFaraone, NicolettaRodriguez, RicaurteFarcas, Anca CorinaRodríguez, SorayaFarcasanu, Ileana C.Rodriguez-Cueto, CarmenFarfan, NorbertoRodriguez-dorantes, MauricioFaria Ribeiro, Ana CristinaRodríguez-Gómez, ArturoFarias-Pereira, RenalisonRodríguez-Jimenes, GuadalupeFarinha, CarlosRodriguez-Monroy, MarcoFarkas, AttilaRodriguez-Morales, OliviaFarkas, EtelkaRodriguez-Rodriguez, JoséFarkaš, PavolRodríguez-Roque, María JanethFarooqi, Ammad AhmadRodríguez-Yoldi, María JesúsFasolato, LucaRodriquez, ManuelaFasoula, VasiliaRodygin, Konstantin S.Fatouros, Dimitrios G.Rodziewicz, PawelFausto, RuiRoeth, DanielFauzi, Mh BusraRogachev, AndreyFavre-Réguillon, AlainRogalska, EwaFawzy, MahmoudRoglans, AnnaFayed, Marwa A. A.Rohanifar, AhmadFeás, XesúsRohwerder, ThoreFecka, IzabelaRoj, EdwardFedorov, Yuri V.Rojas, AdriánFedorova, Olga V.Rojas-Chávez, HugoFedorowicz, Joanna DagmaraRojas-Gomez, Diana MarcelaFeher, GergelyRok, JakubFelletti, SimonaRokavec, MatjazFenaille, FrancoisRolo, JoanaFenelon, KarineRoman, Kamil KrzysztofFeng, QiangRomani, AndreaFeng, Sheng-WeiRomano, DarioFenix, Kevin A.Romański, JarosławFercher, ChristianRombaut, NatachaFerdov, StanislavRomeo, IsabellaFerenc, PajorRomero, MarianoFeresin, GabrielaRomero, NuriaFermo, PaolaRomero-González, RobertoFernadez-Bolanos, José G.Romerosa, AntonioFernandes, Ana I.Romero-Salguero, Francisco J.Fernandes, Elizabeth S.Romo-Mancillas, AntonioFernandes, João Paulo S.Romsdahl, TrevorFernandes, Ricardo M. F.Romualdi, PatriziaFernandes, Tiago A.Roncero, OctavioFernández Leyes, Marcos D.Roosen-Runge, FelixFernandez Ochoa, AlvaroRopciuc, SorinaFernández, InmaculadaRos Berruezo, GasparFernández-García, MartaRos, UrisFernandez-Garcia, RaquelRosa, Derval Dos SantosFernández-Gutiérrez, MarRosales Martínez, AntonioFernandez-Herrera, MariaRosales, RaulFernandez-Lafuente, RobertoRosandi, YudiFernández-Lázaro, FernandoRosenberry, TerroneFernández-Lucas, JesúsRosi, LucaFernández-Prior, María ÁfricaRoso, Juan M.Fernández-Quiroz, DanielRossa, Carlos, Jr.Fernández-Ruiz, JavierRossi, ClaudioFerracini, ChiaraRossi, Jean-ChristopheFerrandez-Villena, ManuelRossi, StefanoFerrando Soria, JesúsRosu, DanFerrante, AntonellaRoszak, SzczepanFerrante, ClaudioRotaru, AurelianFerreira Da Silva, ClassiusRoth, RolandFerreira Santos, Mauro SergioRotondo, ArchimedeFerreira, Ana M.Roullin, V. GaëlleFerreira, FilipeRousar, TomasFerreira, GuilhermeRousis, Nikolaos I.Ferreira, Helena Susana Costa MachadoRovisco, AnaFerreira, Luisa MariaRovito, DanielaFerreira, Luiz Fernando RomanholoRoy, DaipayanFerreira, MarceloRoy, DipankarFerreira, Paula M.Roy, IndranilFerreira, RodrigoRoy, UrmiFerreiro-González, MartaRozenberg, JulianFerrer, GerardoRozwadowski, TomaszFerri, NicolaRóżyło, RenataFerro, Emer SuavinhoRozza, ArianeFersing, CyrilRuan, KeFicai, AntonRuan, XuehuaFierascu, IrinaRuaro, BarbaraFiering, Steven N.Rubattu, SperanzaFierobe, Henri-PierreRubčić, MirtaFigliola, CarlottaRubilotta, EmanueleFigueiras, AnaRubino, Federico MariaFigueroa, MarioRubio, Ramón GonzálezFigueroa, RaulRubio-Arias, HectorFik-Jaskółka, Marta A.Rubio-Martinez, JaimeFilatov, EvgenyRuby-Figueroa, RenéFilatova, SerafimaRudajev, VladimirFilho, Elenilson De Godoy AlvesRuddraraju, Kasi ViswanatharajuFilice, SimonaRudrapal, MithunFilichev, VyacheslavRudzińska, MagdalenaFilip, PetrRufino, Ana Teresa OliveiraFilip, XeniaRufino-Palomares, Eva E.Filipčev, Bojana V.Rugman-Jones, Paul F.Filipe, HugoRui, LixinFilippi, Jean-jacquesRuivo, RaquelFilippi, LucaRuiz Téllez, TrinidadFin, AndreaRuiz, AntonietaFincur, NinaRuiz, EliseoFink, KarinRuiz-Gómez, GloriaFink, RokRuiz-López, Mario AlbertFiorino, FerdinandoRuíz-May, ElielFischer, JanRuiz-Sánchez, EsaúFischer, SilviaRuiz-Torres, VerónicaFischer-Fodor, EvaRuiz-Villafan, BeatrizFitta, MagdalenaRumora, LadaFitzgerald, NeilRuncie, John W.Fizer, Maksym M.Rusakova, IrinaFleet, GeorgeRusanova, IrynaFleszar, MariuszRusinek, RobertFlieger, JolantaRusinska-Roszak, DanutaFlorán, BenjamínRusmini, PaolaFlores, Mario E.Russo Krauss, IreneFlores-López, María L.Russo, EthanFlores-Morales, VirginiaRusso, FrancescaFloresta, GiuseppeRusso, Gian LuigiFlorowska, AnnaRusso, GiuliaFobofou Tanemossu, Serge AlainRusso, LuigiFocaroli, StefanoRusso, MarinaFodor, MariettaRusso, Mario VincenzoFogacci, FedericaRusso, PatriziaFogarasi, MelindaRusso, TeresaFogarasi, SzabolcsRusu, Alina GabrielaFogassy, ElemérRuta, LaviniaFoligni, RobertaRuth Alara, OluwaseunFonseca-Alves, Carlos EduardoRutkowska-Zbik, DorotaFontaine, VéroniqueRutkowski, KonstantyFontana, FabrizioRuwoldt, JostFontana, GianfrancoRuzik, LenaFontana, MarcoRuzza, ChiaraFontana, SébastienRybak, ZbigniewFord, NicoleRybarczyk, Maria K.Forero-Castro, MaribelRybczyńska-Tkaczyk, KamilaForezi, Luana Da Silva MagalhãesRybka, JakubFornarini, SimonettaRycek, LukasFornstedt, TorgnyRychkov, DenisForzato, CristinaRydosz, ArturFossa, PaolaRyms, MichalFoti, AntoninoRyskalin, LarisaFoti, ClaudiaRytel, ElżbietaFouad, HatemRyu, Bo MiFoubelo, FranciscoRyu, JaihyunkFousteris, ManolisRyu, Ji HyunFowkes, RobRyu, Mi-HeonFox, NiamhRyuzaki, SouFracassetti, DanielaRzelewska-Piekut, MartynaFrackowiak, StanisławSa, Jeong-HoonFraga-Corral, MariaSaadat, Yalda RahbarFraile, AlbertoSaady, NooriFraleoni-Morgera, AlessandroSaba, SumbalFranca, Eduardo F.Sabatier, Jean-MarcFrancesca, SelminSabatino, GiuseppinaFranceschini, GiordanoSabatino, LeoFrancescone, RalphSabbah, DimaFrancisco, Ana PaulaSabbasani, VenkataFrancisco, Gómez Aguilar JoséSabbatini, SimonaFranco, Camilo AndresSabbatino, FrancescoFranco, Carlos M.Sabiu, SaheedFranco, DomenicoSabol, IvanFranco, LourdesSacco, ElenaFranco, Marina SantiagoSachin, SharmaFranco, PilarSachs, Laurent M.Franco, WendySadeghi, MahdiFrank, EvaSadeghifar, HasanFrank, Ludmila A.Sadgrove, NicholasFrank, RenéSadiq, Muhammad BilalFranková, JanaSadjadi, SeyedAbdolrezaFrankowski, MarcinSadlej, JoannaFrański, RafałSadok, IlonaFranzini, RaphaelSadoul, KarinFrasson, IlariaSadowska, BeataFratantonio, DeborahSadowski, BartłomiejFrattaruolo, LucaSaeed, MohdFratti, Rutilio A.Safa, Ahmad R.Frau, JuanSafdarian, MehdiFrazzi, RaffaeleSaftić Martinović, LaraFrecer, VladimirSaga, YoshitakaFrediansyah, AndriSagar, Robin P.Freeman, LynettaSagnou, MarinaFreeman, SallySah, HongKeeFreiberg, ArviSaha, RanajayFreitas De Freitas, LucasSaha, SubhasishFreitas, AndreiaSaha, SubhrajitFreitas, MariaSahgal, PranshuFreixa, ZoraidaSahoo, Bikash R.Fries, Pascal H.Saiano, FilippoFrigo, LúcioSaied, Essa M.Frippiat, Jean-PolSaikia, BasantaFritsky, IgorSaikova, SvetlanaFrlan, RokSainlos, MatthieuFröde, Tânia SilviaSaint-Pol, JulienFröhlich, EleonoreSaito, AkikoFröhlich, PeterSaito, KazuyaFroidevaux, RenatoSaito, YoshiroFrolov, AndrejSajeva, MaurizioFrolova, Tatiana S.Sajid, MuhhamadFrontera, AntonioSajnóg, AdamFrosini, MariaSakaebe, HikariFrotscher, MartinSakai, HiromichiFu, LiSakamoto, MasahiroFu, LiansheSakavitsi, Maria EleniFu, QinSakellis, EliasFu, Xiao-AnSakkani, NagarajuFuchi, YasufumiSakr, Tamer M.Fuchs, BethSalafia, FabioFuenmayor, Carlos AlbertoSalafranca Lázaro, Francisco J.Fuentealba, ClaudiaSalak, AndreiFuentes, Jose ManuelSalamanca, Constain H.Fuentes, LidaSalapare, Hernando IIIFuentes-Rivas, Rosa MaríaSalaroli, RobertaFuertes, GustavoSalas, José BlancoFueyo González, FranciscoSałasińska, KamilaFujihara, TetsuakiSałat, KingaFujii, HideakiSalayová, AnetaFujii, TakayoshiSalazar, Juan J.Fujimoto, HiroyukiSalazar, Juan RodrigoFujisawa, KiyoshiSalcedo, RobertoFujisawa, KoichiSaldias, CesarFujita, MorifumiSaleem, MuhammadFujita, WakakoSaleemi, Muhammad KashifFukagawa, HirohikoSaleh, SayedFumagalli, FabioSaleh-E-In, Md MoshfekusFumagalli, LauraSalem, M. Z. MohamedFunazukuri, ToshitakaSaliev, TimurFunes-Ardoiz, IgnacioSalin, Alexey V.Fuochi, VirginiaSalinska, ElzbietaFürer, Sebastian O.Salles, ChristianFurko, MonikaSalluzzo, AntonioFurlan, AlessandroSalmerón, IvanFuruichi, YasuroSalo-Ahen, OutiFuruta, ToshiakiSalomone, AlbertoFusè, MarcoSalom-Roig, Xavier J.Fuse, ShinichiroSalud, SerranoFutakuchi, MitsuruSaluti, GiorgioFuu, SheuSalvador, PedroGabrić, DraganaSalvo, AndreaGabriele, BartoloSalzenstein, PatriceGabriele, MorenaSalzer, IsabellaGabrielli, BrianSamanidou, VictoriaGabrielli, PaoloSamardžić, JelenaGabriško, MarekSamasca, GabrielGackowski, MariuszSamburova, VeraGad, AhmedŠamec, DunjaGad, Heba A.Samec, MarekGadaleta, DomenicoSameera, W. M. C.Gadjanski, IvanaSamhan-Arias, AlejandroGado, FrancescaSamokhvalov, AlexanderGadžurić, SlobodanSampaio, Bruno LeiteGaffke, LidiaSampaio, Geni RodriguesGafurov, MaratSamperi, FilippoGagaoua, MohammedSamykutty, AbhilashGaggiotti, SaraSan Fabian, EmilioGaigneaux, AnthoulaSan Miguel, VeronicaGaikwad, HanmantSancesario, GiuseppeGaizauskas, EugenijusSanches Borges, Ana FláviaGajdoš Kljusurić, JasenkaSánchez Valdés, SaúlGajewska, MałgorzataSánchez, Diego HernánGajtanska, MiladaSanchez, GoarGal, Adrian FlorinSanchez-Andrada, PilarGalabov, AngelSánchez-Dena, OswaldoGalamba Correia, João DomingosSánchez-Gómez, RosarioGalamboš, MichalSanchez-Martinez, MelchorGalanty, AgnieszkaSánchez-Mendoza, AliciaGalazios, GeorgiosSanchez-Perez, ArturoGaldamez, AntonioSanchez-Rodriguez, AntonioGalego, LudovinaSanchis-Gual, RogerGaletti, MariclaSancineto, LucaGaliano, Antonio BernáSandbichler, AdolfGalic, AnteSanders, YanGalić, NivesSandhu, AmandeepGalicia-García, TomásSandjo, Louis PergaudGalina, GenchevaSandlers, YanaGalindo, AgustinSándor, KristyánGalindo, Velia Carolina OsunaSanei, Seyed Hamid RezaGaliniak, SabinaSango, KazunoriGalkin, Alexey P.Sanhueza, LuisGalkina, Svetlana I.Sanjay, RoyGałkowska, DorotaSanjeewa, Kalu Kapuge AsankaGall Troselj, KoraljkaSanjurjo Sanchez, JorgeGallagher, John F.Sannomiya, MiriamGallardo, EugéniaSanoh, SeigoGallegos-Arreola, Martha PatriciaSans, AlbertGallez, BernardSantacruz Ortega, Hisila Del CarmenGalli, FedericoSantamaría, RamónGalli, GiuliaSantana, AdinaGallier, FlorianSantana, Andrés GonzálezGallo, MonicaSantarem, NunoGalluccio, MicheleSantilio, AngelaGalović, OliveraSantini, CarloGalstyan, AnzhelaSantos Junior, Anibal De FreitasGalunska, BistraSantos López, Eva M.Galvão, Adelino M.Santos López, Jorge ArturoGálvez, JulioSantos Mendes, Luis FelipeGamal Mohamed, MohamedSantos, Agenor ValadaresGaman, Mihnea-AlexandruSantos, Carla S.Gamarra, LionelSantos, Célio DiasGambacorta, NicolaSantos, Diana I.Gamiño-Arroyo, ZeferinoSantos, JerranGamov, GeorgeSantos, MónicaGamsjaeger, SonjaSantos, Raquel De Cássia DosGan, RenyouSantos, Willy GlenGan, Samuel Ken-EnSantos-López, GerardoGanatsios, VassiliosSantos-Sánchez, Norma FranceniaGancarz, RomanSanyal, MrinmoyGanesan, A.Saraiva, M. Lúcia M. F. S.Ganesan, KumarSarantopoulou, EvangeliaGanga, María AngelicaSardari, Roya R. R.Gangopadhyay, AnupSarkar, SouravGanguly, PritamSarli, VasilikiGanguly, SayanSarragiotto, MariaGao, BoSaruhan, BilgeGao, ChunshengSasho, GligorovskiGao, JieSatake, HonooGao, JinSatake, MasayukiGao, MengyaoSathyamoorthi, ShyamGarab, GyozoSathyamurthy, RavishankarGarbayo, InésSato, DaisukeGarcía Fernández, José ManuelSato, ShinobuGarcía Martín, Fayna MaríaSato, TakeshiGarcía, JesusSato, YuichiroGarcía, Marta MesíasSatou, TadaakiGarcia, Vitor AugustoSatozono, HiroshiGarcía-Alcocer, GuadalupeSatriano, CristinaGarcia-Bustos, Jose F.Saturnino, CarmelaGarcía-Frutos, Eva MaríaSauer, StephanGarcía-Giménez, RosarioSauka, DiegoGarcia-Giralt, NataliaSaul, NadineGarcía-González, VíctorSaunders, GrahamGarcia-Granda, SantiagoSaura-Sanmartin, AdrianGarcia-Jares, CarmenSava, Bogdan A.García-López, José AntonioSavedboworn, WantichaGarcia-Macia, MarinaSavic Gajic, IvanaGarcía-Martínez, Jesús-MaríaSavić, AleksandarGarcia-Orduña, PilarSavic, Ivan M.Garcia-Salaberri, Pablo A.Savion, NaphtaliGarcia-Santisteban, IraiaSavovic, SvetislavGarcia-Vallve, SantiagoSavvidou, IoannaGardinar, MichaelSawada, Jun-ichiGardini, DavideSawicki, TomaszGargoubi, SondesSayago, Francisco J.Garner, Charles M.Sayed, Ahmed M.Garofulić, IvonaSberveglieri, VeronicaGaroufalis, ChristosScafato, PatriziaGarrido, Narciso M.Scala, Maria CarminaGarrido-Fernández, AntónioScandurra, RobertoGarriga, RosaScarcia, PasqualeGarroni, SebastianoScarso, AlessandroGartia, Manas RanjanScattolin, ThomasGartzia-Rivero, LeireScazzina, FrancescaGarus, SebastianSchabowicz, KrzysztofGarwolińska, DorotaSchatz, JürgenGarza, JorgeSchaufelberger, FredrikGarzoli, StefaniaSchenone, SilviaGaš, BohuslavScherbakov, AlexanderGašić, Uroš M.Scheunemann, MatthiasGaspar, Maria ManuelaSchicker, KlausGáspári, ZoltánSchinzer, DieterGasparri, RobertoSchio, LucaGasperini, SerenaSchipper, KerstinGasque, PhilippeSchiraldi, David A.Gaston, KirkSchleiffer, AlexanderGatesy, John E.Schmidt, BeataGathergood, NicholasSchmidt, FranziskaGatin, AndreySchmidt, MarkGatti, CarloSchmidt, MartinGatti, LauraSchmies, HenrikeGaucherel, CédricSchmitz, ChristianGauden, Piotr A.Schmitz, IngoGauffin-Cano, PaolaSchmitz, UlfGautam, AvneeshSchneider, BerndGavara, NúriaSchneider, ChristophGavara, RafaelSchneider, RaphaelGavrila, Adina IonutaSchnepf, AndreasGavrilov, NemanjaSchöler, Heinz FriedrichGawdzik, BarbaraSchöllhorn, BerndGawel, KingaSchorzman, Allison N.Gaweł-Bęben, KatarzynaSchreiber, IgorGawlik-Dziki, UrszulaSchreiber, MichaelGawliński, DawidSchripsema, JanGazda, PiotrSchröder, VerginicaGazivoda, TatjanaSchroeder, ChristinaGazzano, MassimoSchubert, MarioGdaniec, ZofiaSchulz, BurkhardGdula-Argasińska, JoannaSchulze, JohannesGe, Jun CongSchumann, JuliaGe, MaofaSchutte, StaceyGe, XiaoweiSchuur, BoeloGeary, Timothy G.Schwan, Adrian L.Geerlings, PaulSchwan, WilliamGeetha Bai, RenuSchwans, Jason P.Gell, David A.Schwarz, UlrichGellrich, UrsSchwarzer, ChristophGemma, SandraSciamanna, GiuseppeGendaszewska-Darmach, EdytaSciani, JulianaGeng, Liwei D.Ścianowski, JacekGenoni, AlessandroŚcibisz, IwonaGentile, CarlaScior, ThomasGentile, FabrizioŚciskalska, MilenaGeorgescu (Serb), AlinaScott, PeterGeorgiev, Georgi AsScotti, MarcusGeorgiev, Yordan NikolaevScozzafava, AndreaGeorgieva, MilenaScrimin, PaoloGeorgieva, RadostinaSdrobis, AnamariaGeorgii, RobertSeco, RogerGeorgin, JordanaSecu, Elisabeta-CorinaGeorgopanos, ProkopiosSedlačík, MichalGeraci, CorradaSedmak, GoranGeraldes, CarlosSeebacher, WernerGérard, StéphaneSegatto, MarcoGerasymchuk, Yuriy S.Seger, RonyGerbi, VincenzoSeguí Gil, LucíaGerbino, Darío CésarSegura Plaza, José FranciscoGerdol, MarcoSehgal, RakeshGere, AttilaSeifert, GotthardGeremia, SilvanoSeiquer, IsabelGerlza, TanjaSekar, GiridharGermán-Acacio, Juan ManuelSekar, KarthikeyanGerothanassis, Ioannis P.Sekatski, SergueiGervasoni, JacopoSeki, TakahiroGessler, FlorianSękowski, SzymonGezici, SevgiSeledtsov, Victor IvanovichGhalei, BehnamSeleiman, Mahmoud F.Ghanotakis, Demetrios DemetriosSellars, JonathanGhantasala, MuralidharSelopal, Gurpreet SinghGhasemi, RastaSemenova, Maria L.Ghavam, MansurehSemenyuk, PavelGhavami, SaeidSemenzato, AlessandraGhazali, Sheikh Ahmad Izaddin Sheikh MohdSemerdjieva, Ivanka B.Ghica, Mariana E.Semjon, BorisGhiciuc, Cristina MihaelaSen Gupta, ArnabGhilardi Lago, João HenriqueSen, AliGhimire, BalkrishnaSen, RwikGhițman, JanaSena, PaolaGholami, PeymanSenadeera, WijithaGhomi, MahmoudSenbonmatsu, TakaakiGhonge, TanmaySenćanski, MilanGhosh, PokhrajSenesi, NicolaGhosh, SoumenSengottuvelu, DineshkumarGhosh, SoumitaSengupta, ArkajyotiGiaccone, ValerioSengupta, BidishaGiacomazza, DanielaSenila, LacrimioaraGiampietro, LetiziaSensini, AlbertoGiannakas, ArisSeo, Dong-OhGiannazzo, FilippoSeong, GimyeongGiannoukos, StamatiosSepcic, KristinaGianoncelli, AlessandraSepúlveda, LeonardoGibuła-Tarłowska, EwaSequeda-Castañeda, Luis G.Gica, NicolaeSequeira, César A. C.Gierszewska, MagdalenaSerban, Andreea-irenGikas, EvagelosSerefko, Anna D.Gil, Cristiane DamasŠeregelj, VanjaGil, EricSereno, DenisGilberger, Tim WolfSerezhenkov, VladimirGilburd, BorisSergei, ShtykovGille, LarsSerna, SoniaGillings, Nic M.Serpil, DemirciGil-Mestres, AdriàSerra, MassimoGil-Ramírez, AliciaSerra, StefanoGil-Villegas, AlejandroSerra, VandaGimenes, Sarah CiriloSerrano, Dolores RemediosGiménez-Bastida, Juan AntonioSerrano, GiuliaGinaldi, LiaSerrano-Medina, AracelyGindl-Altmutter, WolfgangSerreli, GabrieleGinestra, PaolaSertić, MirandaGiokas, DimosthenisServa, LorenzoGiordano, AdySeth, Punit P.Giordano, MariaSethi, GautamGiordo, RobertaSeto, ShigekiGiorgetti, LuciaSettu, KalpanaGiorgi, GiorgioSetyaningsih, WidiastutiGiorgini, ElisabettaSetzer, WilliamGiorgio, SelmaSetzer, William N.Giovanella, UmbertoSeverino, PatríciaGiovannoni, Maria P.Sevic, DragutinGiovarelli, MirellaSevilla-Morán, BeatrizGîrd, Cerasela ElenaSeymour, JosephGirelli, Chiara RobertaSeynhaeve, Ann L. B.Giri, SibSforcin, JoséGiromini, CarlottaSgarbossa, AntonellaGiudice, Giuseppe LoShaaban, Khaled A.Giuffrè, Angelo MariaShahbaaz, MohdGiuliano, AristideShahid, Muhammad KashifGiunchedi, PaoloShaik, Gouse PeeraGiurgiulescu, LiviuShaikh, SibhghatullaGiuseppina, BasiniShainyan, BagratGiusti, Anna MariaShajahan, AsifGiziński, DamianShakeel, FaiyazGladkikh, IrinaShakun, AlexandraGladkikh, Natalia A.Shamonin, MikhailGladysheva, Inna P.Shamsuri, Ahmad AdlieGlass, MichelleShan, ShiyaoGlattauer, VeronicaShan, WenqianGlažar, IrenaShan, XindiGlibowski, PawełShang, LuoranGligor, FeliciaShang, ZhuoGligorijevic, NikolaShao, WeiGlinka, AdamShao, YihanGlinton, KevinShapiro, BruceGliozzi, MicaelaSharma, AjitGlišić, BiljanaSharma, BechanGlowinska, EwaSharma, DivyaGlytsis, EliasSharma, KavitaGniazdowska, EwaSharma, ShrikantGnuzzo, GenoveffaSharonova, Olga M.Góbi, SándorShave, StevenGobin, IvanaShee, Nirmal KumarGobis, KatarzynaSheikh Abdul Kadir, Siti HamimahGoclon, JakubSheka, Elena F.Godbout, StéphaneShen, JialongGoddard, AlanShen, JiangchuanGodinho, Maria HelenaShen, PeiyanGodiya, ChiragShen, ShichenGodlewska, PatrycjaShen, TongyeGoeser, FelixShen, WenfeiGoh, SherminShen, YangGok, OzgulShen, You-ChengGoka, KoichiShenderovich, Ilya G.Golani, LalitSheng, RuilongGold, AvramShenkarev, Zakhar O.Goldeman, WaldemarSher, FarooqGoldoni, LucaSheremet, MikhailGolgovici, FlorentinaSherman, EmmaGolmohamadi, AmirSherman, MichaelGolombick, TerrySherwood, JamesGolovin, Andrey V.Shestopalov, Michael A.Golub, IgorSheu, Jyh-HorngGomes, Ana Teresa P. C.Shi, chengGomes, NelsonShi, KaiyuanGomes, SóniaShi, QuanGomes-da-Silva, Lígia C.Shi, YangGómez Bravo, Carlos AlfredoShi, Yu GangGómez Castellà, CristinaShi, ZhiGómez De Cedrón, MartaShiau, LiedingGómez García, Carlos J.Shibahara, FumitoshiGomez, JesusShibata, HidekiGómez, JuanShibata, IkuyaGomez, NidiaShibatomi, KazutakaGómez-Graña, SergioShieh, Jeng-JerGómez-Manzo, SaúlShiga, TakuyaGómez-Ríos, DavidShih-Chung, WeiGomha, SobhiShikov, AlexanderGomis-Cebolla, JoaquinShikov, Alexander N.Goncalves, AlexandreShimabukuro, MasayaGonçalves, Ana CristinaShimada, YasuhitoGonçalves, VirginiaShimamoto, NobuoGonda, SándorShimizu, YoichiGondek, EwaShimpi, ManishkumarGong, Han-YuanShin, JongheonGonkowski, SławomirShin, SangwooGonzales, EvaShin, Seung-HeonGonzales, Ralph RollyShin, Young G.González Laredo, Rubén F.Shinada, TetsuroGonzález Maglio, Daniel H.Shinbayashi, TakuyaGonzález, Florenci V.Shinde, AparnaGonzalez, Miguel ToroShindyapina, Anastasia V.Gonzalez-Alvarez, MartaShinomiya, KazufusaGonzález-Baró, Ana C.Shirahata, TatsuyaGonzalez-Casado, AntonioShirokov, Vladimir B.González-Cortazar, ManasésShiuchi, TetsuyaGonzález-Cruz, LeopoldoShivachev, BorisGonzález-Delgado, Jose A.Shkondrov, AleksandarGonzález-García, JorgeShkryl, Yuri N.González-Laredo, Rubén FranciscoShmarakov, IgorGonzález-Lezana, TomásShoji, KanGonzález-Marrero, JoaquinShopska, VeselaGonzález-Maya, LeticiaShow, PaulokeGonzalez-Menendez, PedroShrestha, NamitaGonzález-Minero, Francisco JoséShrivastava, IndiraGonzález-Muñiz, RosarioShukitt-Hale, BarbaraGonzalez-Neves, GustavoShurpik, DmitriyGonzález-Pérez, José A.Si, MisiGonzález-Reza, RicardoŠic Žlabur, JanaGonzález-Sálamo, JavierSichaem, JirapastGonzález-Vergara, EnriqueSiddiqui, MasoomGonzález-Zamora, EduardoSiddiqui, Mohammed Rafiq H.Góra, ArturSideratou, ZiliGoradia, NishitSiedlecka, RenataGorbacheva, LiubovSiejak, PrzemyslawGordeliy, ValentinSieniawska, ElwiraGórecki, TadeuszSieradzan, AdamGori, AntonellaSierra, ArturoGorinstein, ShelaSignore, GiovanniGoris, TobiasSigolaeva, Larisa V.Gorkunov, MaximSihan, ZhaoGornostaev, Leonid M.Sikora, MariuszGornowicz-Porowska, JustynaSilacci, PaoloGoroncy, Alexander K.Silaghi-Dumitrescu, RaduGórska, SabinaSilberring, JerzyGórska-Drabik, EdytaŠiler, BranislavGorynski, KrzysztofŠilha, DavidGoswami, NirmalSilina, YuliyaGoswami, SumitaSilva, Amelia M.Goto, KeiSilva, Ana Margarida G.Goto, MotonobuSilva, AndreGoto, YuheiSilva, Andréa Alice DaGotsbacher, MichaelSilva, Artur M. S.Gotzias, AnastasiosSilva, AuroraGoulas, VlasiosSilva, Carlos AlbertoGould, RobertSilva, EdsonGoupil, PascaleSilva, Eric KevenGouveia, Julia RochaSilva, FranciscoGouveia, SusanaSilva, JoãoGovindarajulu, ManojSilva, JoséGowda, KrishneSilva, Lorena Mara Alexandre E.Goya, LuisSilva, PedroGozdowska, MagdalenaSilva, RicardoGrabarczyk, MałgorzataSilva, RuiGrabarek, Beniamin OskarSilva, SóniaGraber, Zachary T.Silva-Júnior, Edeildo FerreiraGrabowski, SlawomirSilvan, Miguel MansoGrabska-Zielińska, SylwiaSilveira, DamarisGracia, LourdesSilvennoinen, Olli JuhaniGraczykowski, BartlomiejSilvestre, Armando J. D.Graebin, CedricSilvestre, SamuelGragnano, FeliceSilvestri, DanieleGraham, AnnetteSilvino, PedroGrajewski, JakubSim, UkGramatica, PaolaSimanek, EricGramignoli, RobertoŠimek, MilanGramza – Michałowska, AnnaSimeonov, VasilGranados-Principal, Sergio M.Simionato, Ana Valéria C.Grande Tovar, Carlos DavidSimirgiotis, MarioGrande, FedoraSimmchen, JulianeGrande, RaffaeleSimões, João Carlos CaetanoGrandinetti, FeliceSimões, SandraGrandini, Carlos RobertoSimon, JozsefGranica, SebastianSimonescu, Claudia MariaGrassi, MarioSims, MichaelGray, AlisonSinclair, AndrewGrazioli, CesareSinebryukhov, Sergey LeonidovichGreatrex, Ben W.Singh, Ajay VikramGreber, KatarzynaSingh, AmrinderGreco, NicolaSingh, AnandGrée, RenéSingh, AnikaGreen, BenSingh, HarbinderGreenhalgh, DavidSingh, KamalendraGreenlief, C. MichaelSingh, ParveshGrembecka, MałgorzataSingh, RanbirGrieco, DomenicoSingh, SarbjitGriesbeck, Axel G.Singha, SubhankarGriesinger, ChristianSipos, EmeseGrillo, GiorgioSipos, PálGrimaldi, AnnalisaSippl, WolfgangGrimaldi, ManuelaSiramshetty, Vishal B.Grimaldi, PaolaSirohi, SunilGrimm, Wolf-DieterSironić, AndrejaGrishko, VictoriaSirugaloor, SenthilkumarGrivas, IoannisSiudem, PawełGriveau, SophieSivakumar, ManiGrobe, KaySivakumar, NallusamyGrochowicz, MartaSivakumar, PoopalasingamGroeneman, RyanSivaraj, Kishor KumarGromov, Alexander A.Skalicka-Woźniak, KrystynaGronowski, MarcinSkapin, Sreco D.Gros, Claude P.Skarżewski, JacekGross, Andrew JamesSkinkis, PattyGrosser, AnnaSkirda, Vladimir D.Grossi, GiancarloSkliros, DimitriosGrossman, JakeSkoczko, IwonaGrosso, ClaraSkok, MarynaGrosso, MichelaSkorik, Yury A.Grosso, Robson L.Skouras, PanagiotisGrosu, IonSkvortsov, IvanGrote, KarstenŚlachciński, MariuszGrotli, MortenSlawin, AlexandraGrubešić, Renata JurišićSlawomir, BakierGrujić-Milanović, JelicaSlim, SmaouiGrulova, DanielaSliwinski, TomaszGrumezescu, Alexandru MihaiSliwka, Hans-RichardGryszkin, ArturŚliwka-Kaszyńska, MagdalenaGrzegorczyk, IzabelaSłoczyńska, KarolinaGrzesiuk, ElżbietaSlominski, AndrzejGrzywacz, DariaSlouf, MiroslavGu, BingŚlusarczyk, SylwesterGu, HongzhouSmeriglio, AntonellaGu, JinzhongSmiesko, MartinGu, JiweiŚmigielski, Krzysztof B.Gu, SiyiSmiljanić, KatarinaGu, YueSmirnov, Konstantin S.Guajardo, NadiaSmirnov, MichaelGuan, YihuiSmith, David P.Guaragna, AnnalisaSmith, Mark A.Guarino, SalvatoreSmith, MicholasGuarraci, FaySmith, RobertGuarracino, Danielle A.Smith, StewartGuarrasi, ValeriaSmolarczyk, RomanGuazzelli, LorenzoSmolensky, DmitriyGubica, TomaszSmonou, IouliaGubitosa, JenniferSmulko, JanuszGudapati, HemanthSmyk, BogdanGude Rodríguez, LourdesSnapper, MarcGudkov, SergeySnegur, LubovGuduru, ShivaSnežana, PapovićGuella, GrazianoSnow, Nicholas H.Guérardel, YannSnowden, TimothyGuerra-Librero, AnaSoares, SofiaGuerreiro, OlindaSobeh, MansourGuerrieri, NicolettaSoberón-Chávez, GloriaGuglielmi, PaoloSobiech, MonikaGuichou, Jean FrancoisSobiesiak, MagdalenaGuidi, PatriziaSobolev, ArkadijGuillen Fretes, Rosa MariaSobral, HugoGuillén-Bejarano, RafaelSocaciu, CarmenGuillen-Grima, FranciscoSocas-Rodríguez, BárbaraGuillot, BenoîtSocha, RobertGuillou, Claude G.Soengas, Raquel G.Guimarães, DianaSoeta, TakahiroGuiné, Raquel P. F.Sofi, Sajad AhmadGuitton, JérômeSofie Møller, MarieGulbinas, AntanasSogawa, NorioGulea, MihaelaSohn, YoungkuGulicovski, JelenaSohrabi, SalmanGullon, BeatrizŠojić, BranislavGülses, AydinSojoudi, HosseinGumerova, Nadiia I.Sokalski, AndrzejGumieniczek, AnnaSokalski, W. AndrzejGumienna, MałgorzataSokolova, Maria P.Guminska, JolantaSokolova, Maria PetrovnaGumuscu, BurcuSokolová, RomanaGunjević, VeronikaSokovic, MarinaGun’ko, Vladimir M.Šolaja, Bogdan A.Günther, StefanSolano, FranciscoGuo, BingSolcan, CarmenGuo, DabinSoler, Miguel ÁngelGuo, JiahuaSolin, NiclasGuo, JingshuSolís, Luis Fernando MagañaGuo, Ming-quanSolis, Octavio ElizaldeGuo, NaSołoducho, JadwigaGuo, YannaSolovieva, SvetlanaGuo, YongSolovyev, PavelGuo, YouzhongSolyanikova, Inna P.Gupta, DhanojSomoza, Mark M.Gupta, KuldeepSon, Ki-HoGupta, MohitSon, Sang-KilGupta, Tripti ThapaSong, FuhangGuran, RomanSong, KenanGurgu, Leontina C.Song, Myoung ChongGurzadyan, GagikSong, PingGusarov, Sergey I.Song, QishiGuselnikova, Olga AndreevnaSong, Tze-BinGusev, AlexanderSong, XinbingGushchin, Artem L.Song, YuGus’kov, VladimirSoni, NisargGutbier, SimonSoni, PraveenGutiérrez Ramírez, ManuelSonowal, HimangshuGutiérrez-Gamboa, GastónSood, RamanGuven, OnurSoond, Surinder M.Guzmán, EduardoSorbi, ClaudiaGuzman-Quevedo, OmarSordon, SandraGwiazdowska, DanielaSoreq, HermonaGyémánt, GyöngyiSorice, MaurizioHa, EunyoungSorsa, TimoHa, Hyung-HoSortais, Jean-baptisteHa, Hyun-JoonSosa Díaz, TeresaHa, In JinSosa, Filipe Hobi BordónHa, Ki-TaeSosic, IzidorHaaparanta-Solin, MerjaSosio, MargheritaHabashi, FathiSotelo-Mundo, Rogerio R.Habib, AhsanSoto, PaulHać-Szymańczuk, ElżbietaSoto-Cruz, IsabelHada, MasahikoSotthewes, KaiHadjiivanov, Konstantin IvanovSoucek, LauraHadjikakou, SotirisSousa, Joana L. C.Hadjipavlou-Litina, DimitraSousa, João C.Haeckl, JakeSousa, Maria JoãoHaeussler, AnitaSousa, Sérgio FilipeHagège, AgnèsSouza, Israel Donizeti DeHagelueken, GregorSóvágó, ImreHaghi, MehraSoveral, GraçaHahn, Jae RyangSowa, IreneuszHaidar, SamerȘpac, AdrianHaidar, Ziyad S.Spada, LorenzoHaider, JunaidSpadaro, AngeloHajduk, BarbaraSpallarossa, AndreaHalberstadt, NadineŠpanič, ValentinaHalcovitch, Nathan R.Spanò, NancyHalevas, EleftheriosSpanu, DavideHalim, Mohd MahadiSpasojevic, AnneHalim, SobiaSpasov, Alexander A.Haliński, Łukasz P.Spătaru, NiculaeHall, Carl AlanSpeijer, DaveHallegger, MartinaSpeina, ElżbietaHallmann, EwelinaSpelzini, DaríoHaltiwanger, RobertSpencer, WalseHamedpour, VahidSpicer, Christopher D.Hamidi, YoussefSpiegler, VerenaHamman, JoiasSpiers, AndrewHamon, Jean-RenéSpinelli, LucianaHamperl, StephanSpínola, VítorHan, DongSpiridigliozzi, LucaHan, Dong WoonSpissu, YleniaHan, Mei-LingSpivak, DavidHan, SifeiSpochacz, MartaHan, SunwooSpur, Bernd W.Han, WenboSpychaj, RadosławHan, YiweiSpychaj, TadeuszHanafy, Nemany A. N.Spyridopoulou, KaterinaHanai, ToshihikoSquillacioti, CaterinaHanawa, TakehisaSreckovic, Vladimir A.Hǎncianu, MonicaSreerama, LakshmaiahHancu, GabrielSridhar, KandiHandayani, MurniSrinivasan, AlagarsamyHanganu, DanielaSrivastava, PranayHanif, NovriyandiSroka, ZbigniewHanifehpour, YounesSroka-Bartnicka, AnnaHanik, NilsSt John, Franz J.Hannan, Md. AbdulStacchiotti, AlessandraHannisdal, RitaStadlbauer, SvenHansen, NielsStadlmann, JohannesHanus-Fajerska, EwaStaedler, DavideHao, JihuaStafford, James L.Hao, JinsongStamatatos, TheocharisHao, MingangStamatin, Serban N.Hapke, MarkoStamatis, NikolaosHaponiuk, JózefStampor, WaldemarHaque, ArifulStan, LauraHaque, Md. MamunulStan, MirunaHarald, ClausStancu, Camelia SorinaHarasym, JoannaStaniak, MariolaHardacre, AllanStaniek, HalinaHardtke-Wolenski, MatthiasStanila, AndreeaHarduin-Lepers, AnneStanimirova, IvanaHardy, MicaelStanisz, BeataHarijan, Rajesh K.Staniszewska, MagdalenaHarp, Joel M.Stankov, KarmenHarriman, AnthonyStankovic, MilanHarrington, BrittneyStanzione, MariameliaHarris, MuhammadStappen, IrisHartanto, YusakStarecki, TomaszHartl, SandraStarek, AgnieszkaHartman, MariuszŠtarha, PavelHartman, Matthew C. T.Stark, Felicity C.Hartmann, PeterStarkuviene, VytauteHartmann, RobertStarowicz, MałgorzataHas, CristinaStarz-Gaiano, MichelleHasan, ImtiajStasevych, MarynaHasan, MehediStasiak-Różańska, LidiaHasan, RaquibulStaszak, KatarzynaHasegawa, MorifumiStathatos, EliasHashim, KhalidStatti, GiacarloHashimoto, MakotoStaudacher, ErikaHashimoto, TakeshiStavrianidi, AndreyHassan, AhmedStawicka, KatarzynaHassan, ErrolSteber, AmandaHassan, FahmySteenekamp, JanHassan, MohamedStefan, Sven MarcelHassan, Sherif T. S.Stefanczyk, OlafHassanali, Ali A.Stefanelli, ManuelaHasse, SybilleStefanescu, RuxandraHatano, TsutomuStefania, FilosaHathout, RaniaStefania, RacheleHatzdimitriou, EffieStefaniak, IreneuszHaudecoeur, RomainŠtefanić, Petra PeharecHaupt, KarstenŠtefániková, JanaHavelek, RadimStefaniu, AmaliaHay, Moya AnneStefanowicz, PiotrHayashi, NaotoStefanska, AnnaHayashi, YoshihitoStefanucci, AzzurraHaynes, RichardStefanuto, Pierre-HuguesHazai, LászlóSteinbücher, MihaHazekawa, MaiStelios, CourisHazer, BakiStellato, FrancescoHazra, SusantaStelzer, FranzHe, DelongStepanenko, Olesya V.He, GuanjieStepanic, VisnjaHe, HaishengStepanova, EkaterinaHe, JianStepanova-Khramtsova, EkaterinaHe, LiangcanStephenson, Mary C.He, MingyuStepnicka, PetrHe, XinStetsyshyn, YurijHe, ZhiqiSteuer, ChristianHe, ZhongqiSteup, MartinHearn, Michael J.Stilhano, Roberta S.Heber, DieterStjepanovic, DanielHeckman, Caroline A.Stobiecka, MagdalenaHeeb, StephanStoch, PawełHegazy, Mohamed-Elamir F.Stock, Naomi L.Hegedűs, LászlóStoev, StoichoHegedűsová, AlžbetaStogniy, Marina YuHeine, ElisabethStoica, AnicutaHeinrich, BenoîtStoika, Rostyslav S.Heise, Herbert MichaelStojakowska, AnnaHeitz, Mark P.Stojanović, ZoricaHelesbeux, Jean-JacquesStojmenović, MarijaHellebust, StigStojsavljević, AleksandarHempel, GeorgStokowa-Sołtys, KamilaHemraz, Usha D.Stone, Kari L.Henderson, BrandonStoyanova, AlbenaHengesbach, MartinŠtrac, Dubravka ŠvobHengjia, NiStraface, ElisabettaHennig, SvenStrahl, SabineHenri, JulienStranges, StefanoHenschel, JonasStrati, Irini F.Herman, Andrzej P.Strazewski, PeterHermann, PetrStreckiene, GiedreHernández Núñez, EmanuelStreicher, John M.Hernández, AgustínStrianese, MariaHernández, FranciscaStrout, Douglas L.Hernández-Carlos, B.Stroylova, Yulia YuHernandez-Chiñas, UlisesStrozzi, MatteoHernández-García, SamantaStrube, JochenHernández-Montelongo, JacoboStruck-Lewicka, WiktoriaHerosimczyk, AgnieszkaStruga, MartaHerr, PatrickStrunskus, ThomasHerráez-Hernández, RosaStrunz, Célia Maria CássaroHerrera Zaldivar, ManuelStrzelak, KamilHerrero, HernándezStrzemiecka, BeataHerres-Pawlis, SonjaStrzemski, MaciejHerzig, VolkerStuchlík, StanislavHetényi, CsabaStudzińska, SylwiaHeyder, RodrigoStudzińska-Sroka, ElżbietaHiberty, Philippe C.Stuparu, MihaielaHibi, TakaoSturm, ReneaHickey, NealStuzhin, PavelHidalgo, ManuelaStyskalik, AlesHiebert, PaulSu, ChengyongHiebl, BernhardSu, Chie-ShaanHigashi, NobuakiSu, CongHigashi, NobuyukiSu, PeifengHigo, ShimpeiSu, Wen-TaHii, Charles S.Su, XiaojunHill, MartinSu, XinHinds, Terry D.Suarez-Alcantara, KarinaHirano, YoshiakiSubbiahdoss, GuruprakashHirasaka, KatsuyaSubedi, DineshHirata, TetsuyaSubhadra, BinduHirata, YokoSubianto, SuryaHiroaki, HidekazuSubirats, XavierHirobe, SachikoSubramani Paranthaman, BalasubramaniHiscock, JenniferSubramanian, ParthasarathiHiziroglu, SalimSubudhi, SonuHlebova, MiroslavaSuchodolski, JakubHnatejko, ZbygniewSuckling, Colin J.Ho, Paul Chi LuiSudarikov, Denis V.Ho, Wing ShingSuddala, Krishna ChaitanyaHocek, MichalSue, Shih-CheHochlaf, MajdiSugahara, TakeshiHofbauer, StefanSugamata, KohHoffmann, Markus M.Sugawara, AkihiroHofmann, BettinaSugawara, ShigekiHogan, SeanSugiki, ToshihikoHohmann, Urszula GrabiecSuhandy, DidingHolder, AlvinSuhito, Intan RosalinaHolešová, SylvaSujkowska-Rybkowska, MarzenaHollywood, MarkSukhova, EkaterinaHomann, ThomasSulej, JustynaHong, LiangzhiSulek, AnnaHongjie, PengSulka, Grzegorz DariuszHooda, JagmohanSułowicz, SławomirHoracek, MichaSülsen, ValeriaHorbatsch, MarkoSumbatyan, NataliaHorel, ÁgotaSun, Chia-LiangHorhat, DeliaSun, JunHorii, YojiSun, MengtaoHorn, AnselmSun, Qing-FuHornak, Joseph P.Sun, Shih-JyeHornedo-Ortega, RuthSun, Wen-HuaHorsky, JiřiSun, XiangchengHortigón-Vinagre, MaríaSun, XiuxiuHorton, JohnSun, Yun-LuHorvath, DragosSunada, YusukeHorváth, KrisztiánSunasee, RajeshHorváth, OttóSundararajan, RajiHosen, AkterSundman, OlaHosokawa, NobukoSung, Wen-ChiehHossain, Md AshrafSuplatov, Dmitry A.Hosseinnezhad, MozhganSuresh, KuthuruHošťálková, AnnaŠurina, IgorHosur, VishnuSurmenev, Roman A.Hosztafi, SandorSusanna, CogoHou, Chih YaoSusi, ZaraHou, ChongSut, StefaniaHouee, ChantalSutherland, Hamish S.Hour, Mann-JenSutherland, James D.Houston, DouglasSutton, Adam T.Hoyos, PilarSuwan, KeittisakHrabal, RichardSuzuki, TakahiroHrbáĉ, JanSuzuki, YukiHrenar, TomicaSuzutani, TatsuoHryniewicka, AgnieszkaSuzzi, GiovannaHsia, Shih-MinŠvedienė, JurgitaHsieh, Chang-WeiSvetec, Ivan KrešimirHsieh, Lu-ShengSvirko, YuriHsieh, Ming-JuSvirshchevskaya, ElenaHsieh, Pei-WenSvoronos, Paris D. N.Hsieh, ShulingSwairjo, Manal A.Hsieh, Tusty-JuanSwami Vetha, Berwin SinghHsieh, Wen-TsongSwarup, SanjayHsieh, Yi-HsienSwatko-Ossor, MartaHsin, Kun-YiŚwiątek, ŁukaszHssaisoune, MohammedŚwiątek, PiotrHsu, Hao-JenSwiatly-Blaszkiewicz, AgataHsu, Pang-HungŚwiderski, GrzegorzHsu, Sodio C. N.Święch, DominikaHsueh, Yi-HuangŚwit, GrzegorzHu, DaodaoŚwit, PawełHu, HaoSychrovsky, VladimirHu, JinmingSyguda, AnnaHu, LianmeiSykłowska-Baranek, KatarzynaHu, MingyouSykora, DavidHu, NaSýkora, JanHu, TianhuiSykuła, AnnaHu, YingwenSyngouna, VasilikiHu, YuanSynytsya, AndriyHu, ZhigangSyrpas, MichailHua, RuimaoSytar, OksanaHuang, ChangshuiSzabó, AndrásHuang, Cheng-YangSzabó, RenátaHuang, ChiayiSzaciłowski, KonradHuang, Chun-YingSzacon, ElzbietaHuang, Chun-YungSzafraniak-Wiza, IzabelaHuang, Dong-MingSzafraniec-Szczęsny, JoannaHuang, Hui-ChiSzafranski, KrzysztofHuang, JianfengSzala, MarcinHuang, Jui-HsienSzaleniec, MaciejHuang, JunfengSzałowski, KarolHuang, KunSzántay, Csaba, Jr.Huang, Rong-NanSzczepanik, DariuszHuang, XudongSzczęśniak, BarbaraHuang, YadongSzczubialka, KrzysztofHuang, YanliangSzczukowski, ŁukaszHuang, YuSzegedi, AgnesHuang, ZhuangrongSzeiffova Bacova, BarbaraHuber, RobertSzeleszczuk, ŁukaszHuber, Roland G.Szeliga, MagdalenaHuber, TimSzeto, Savio Yim TongHubicka, UrszulaSzewczyk, AgnieszkaHübner, FlorianSzewczyk, KatarzynaHübner, RalphSzilágyi, LászlóHuck, OlivierSzklarzewicz, JanuszHuclier, SandrineSzlęk, JakubHuczyński, AdamSzlósarczyk, MarekHudita, ArianaSzłyk, EdwardHudz, NataliaSzoka, ŁukaszHuefner, AntjeSzopa, AgnieszkaHuh, Joo YoungSzostak, RomanHuh, SeongSzpyrka, EwaHuisman, MarcSztanke, KrzysztofHumayun, MuhammadSztanke, MałgorzataHung, Shih-HanSztiller-Sikorska, MalgorzataHung, Tai-FengSzulczyk, DanielHung, Yao-ChingSzumny, AntoniHunter, LukeSzwajca, AnnaHunter, Robert P.Szweda, PiotrHuntosova, VeronikaSzybowicz, MiroslawHursthouse, Andrew S.Szydlowska, AleksandraHussain, AfzalSzyja, Bartłomiej M.Hussain, HidayatSzymandera-Buszka, KrystynaHussain, SaddamSzymańki, PawełHuszcza, EwaSzymanowska, UrszulaHutter, MichaelSzymańska, Iwona B.Hwang, Gi-ByoungSzymczyk, AnnaHwang, In KooTaamalli, AmaniHwang, Long-ChihTabanca, NurhayatHwang, YuhoonTabaran, FlaviuHwangbo, CheolTabaszewska, MałgorzataHwu, Jih RuTabata, YasuhikoHyun, Tae KyungTachibana, ShinjiroIacobucci, IlariaTacias-Pascacio, Veymar GuadalupeIacumin, LucillaTadić, VanjaIannacchione, Germano S.Tafelska-Kaczmarek, AgnieszkaIannotti, Fabio ArturoTafuto, SalvatoreIannuccelli, ValentinaTaghavi, SomayehIatridi, ZacharoulaTagliatesta, PietroIbáñez, Pablo Rafael PardoTaglietti, Angelo MariaIbarretxe, JulenTaguet, AurélieIbba, Maria ItriaTaha, AhmedIbrahim, HasimTaha-Tijerina, JaimeIbrahim, MasoomaTaherzadeh, GhazalehIbrahim, Mohamed AliTaillebois, EmilianeIbrahimi, ManarTaiti, CosimoIchikawa, NatsukoTajti, ÁdámIchikawa, TakahiroTakada, AkihikoIdoate, MiguelTakagi, KiyoshiIelciu, IrinaTakahashi, FumikiIghigeanu, DanielTakahashi, HidenoriIglesias, Bernardo AlmeidaTakahashi, JacquelineIglesias, MartaTakahashi, KaitoIglesias-Carres, LisardTakahashi, KazuoIgnatowicz, KatarzynaTakahashi, MasakiIgne, BenoîtTakahashi, MasayukiIgnés-Mullol, JordiTakahashi, NobuakiIgnjatovic, NenadTakahashi, ShuntaroIgor, GornushkinTakahashi, ToshiyukiIhlemann, JürgenTakano, ToshiyukiIhmels, HeikoTakemura, HiroyukiIkeda, MasaomiTakenaka, ShigeoriIkeda, Tatsuya M.Takeuchi, HideyukiIkuta, NaokoTakikawa, OsamuIlagan, Ma. Xenia G.Takiyama, HiroshiIlaš, JanezTakizawa, ShinobuIlavsky, JanTałałaj, Izabela AnnaIlia, GheorgeTalapko, JasminkaIlia, GheorgheTaleb, AbdelhafedIlias, MiroslavTalmon, MariaIlic, NebojsaTalora, ClaudioIlieva, DarjaTalotta, CarmenIllescas, JavierTam, Lik-HoImai, JunTamaru, YutakaImam, SyedTamba, Bogdan IonelImamoto, YasushiTambaro, SimoneImamura, RyuTamura, KazuhiroImbimbo, BrunoTamura, KojiImhof, PetraTamura, TakahikoImperatore, RobertaTan, LiImperio, DanielaTan, YanglanImprota, RobertoTan, YingInan, SaadetTan, YutingInclán, MarioTanaka, MasatoIndurthi, DineshTanaka, MikiyaInestrosa, Nibaldo C.Tanasova, MarinaInglot, TadeuszTanco, SebastianIngrassia, Lorenzo PaoloTaneja, IshaIñiguez-Moreno, MaricarmenTang, ChongchongInostroza-Blancheteau, ClaudioTang, DamuInturri, RosannaTang, FujianInyushin, Mikhail Y.Tang, Gui-HuaIommelli, FrancescaTang, HaoyuIonita, PetreTang, JinglongIorizzi, MariaTang, LongtengIqbal, Hafiz M. N.Tang, Ri-YuanIrina, CarlescuTang, Shi-YangIsbrandt, MarekTang, Soon YewIshida, NaoyaTang, Ye-FengIshigaki, YasuhitoTangadanchu, Vijai Kumar ReddyIshii, DaisukeTani, FumitoIshizuka, TakumiTania Prontera, CarmelaIskra, JernejTaniguchi, MoyuIslam, AtaulTanizaki, YutaIslam, Md AminulTankiewicz, MaciejIslam, Md RashadulTannous, JoannaIslam, Mohammad MohiminulTantardini, ChristianIslam, SalmanTantos, ÁgnesIslas Osuna, Maria AuxiliadoraTao, Ya-XiongIslas-Jácome, AlejandroTao, YunwenIsman, Murray B.Tapia, Alejandro A.Ismaya, Wangsa TirtaTarasiuk, JolantaIsola, GaetanoTarcea, MonicaIsopescu, RalucaTarditi, Ana MariaIsozaki, KatsuhiroTariq, MohammadIsrar, Muhammad ZubairTarko, TomaszIstrate, BogdanTarraf, WaedIstyastono, Enade PerdanaTascon, MarcosIsvoran, AdrianaTashima, ToshihikoItabaiana, IvaldoTashiro, MitsuruItabashi, YutakaTasic, LjubicaIto, Jun-ichiTasneem, Shumaila AmanIto, ShunichiroTassieri, ManlioIto, TakamichiTasso, BrunoIto, TakuhiroTatarczak-Michalewska, MałgorzataItoh, ToshiyukiTatsuhiro, KojimaItsuno, ShinichiTava, AldoIturriaga-Vasquez, PatricioTavani, CinziaIvanets, AndreiTavanti, FrancescoIvankov, Dmitry N.Tavares, Josean FechineIvanov, IliyanTavoloni, TamaraIvanov, MarijaTayebati, Seyed KhosrowIvanov, Vladimir K.Taylor, Jason G.Ivanova, Juliana M.Tchekalarova, JanaIvanović, MilenaTchertanov, LubaIvanovska, NinaTchoukov, PlamenIvshina, IrenaTedeschi, PaolaIwabuchi, YoshiharuTedesco, IdoloIwai, AtsushiTeeranachaideekul, VeerawatIwaï, HideoTeixeira, Guilhermina FerreiraIwai, Leo K.Teixeira, João M. C.Iwanicki, AdamTeixeira, Marcos F. S.Iwasaki, MasaokiTeixeira, Maria C.Iwashina, TsukasaTeixidó, JordiIzak, PavelTeixidor, FrancescIzumi, KenjiTejedo, PabloJablan, JasnaTelatin, AndreaJabłońska-Trypuć, AgataTelbisz, AgnesJablonski, MiroslawTelles Biasoto, Aline CamarãoJabraoui, HichamTéllez Valencia, AlfredoJacak, LucjanTéllez-Téllez, MauraJacob, ChristopheTéllez-Valencia, AlfredoJacob, GopasTembu, JacquelineJacob, Maik H.Temel, AslihanJacobo-Velazquez, DanielTempera, GiannaJacobsen, ElisabethTemraz, SallyJaeger, PhilipTeneva, Desislava GeorgievaJaffe, MichaelTeng, PengJaffrezic, NicoleTenorio-Alfonso, AdriánJaganjac, MoranaTeobald, KupkaJäger, MartinTeodora, CosteaJagerovic, NadineTeodorescu, FlorinaJagga, SupriyaTeodorescu, Valentin ŞerbanJahan, SadafTeodoro, Anderson JungerJahangir, IfatTeplyakov, VladimirJain, DharamdeepTepper, GaryJaiswal, Jagdish KumarTeramoto, NaozumiJakas, AndrejaTerán, CarmenJakimovski, DejanTercjak, AgnieszkaJakobusic Brala, CvijetaTerec, AnamariaJakovlevic, DraganaTerentyev, AlexanderJakovljevic, VladimirTermine, RobertoJakubczyk, KarolinaTermopoli, VeronicaJakubek, MilanTerrasson, VincentJakubíková, JanaTerraza, Claudio A.Jaleel, AbdulTerrinoni, AlessandroJalil, AbdelTeslić, NemanjaJama-Kmiecik, AgnieszkaTessem, Jeffery S.Jambrina, Pablo G.Teter, KenJames, Nicholas G.Tetsuo, OkadaJameson, CynthiaTeusch, NicoleJameson, DavidTezuka, YasuhiroJamin, NadègeThakur, Vijay KumarJamrógiewicz, MarzenaThamban Chandrika, NishadJan Laskowski, ŁukaszThangavel, ChellappagounderJana, AtanuThanh Nhut, DoJana, BimalThannickal, Thomas C.Jana, NavenduThapa, ManojJancikova, SimonaThapa, SurendraJanda, KatarzynaTheerthagiri, JayaramanJandl, KatharinaTheis, ThomasJaneček, ŠtefanTheocharidou, AnnaJang, CholsoonTheoduloz, CristinaJang, HohyounThi, Thai Thanh HoangJang, JaewonThiebault, ThomasJangra, HarishThirumalai, DinakaranJaniak, MichalThiruvengadam, MuthuJanić Hajnal, ElizabetThoma, MarkusJanicka, MałgorzataThoma, Markus H.Jankiewicz, UrszulaThomas, ChloeJankowska, ElzbietaThomas, Eric JimJanovák, LászlóThomas, SajeshJans, Judith J. M.Thomas, Stephen P.Jantrawut, PensakThorek, DanielJanus, WladyslawThulstrup, Peter W.Janzen, Daron E.Tiago, GonçaloJaradat, NidalTian, GuocaiJarak, IvanaTiano, LucaJaramillo-Morales, Osmar AntonioTidgewell, KevinJaramillo-Quintero, Oscar AndrésTikhonov, Aleksei YaJarocka-Karpowicz, IwonaTikhonova, MariaJaroń, TomaszTilea, IoanJarończyk, MałgorzataTimar, Maria CristinaJaros, Sabina W.Timmerman, PeterJarosz, SlawomirTimofeev, SergeyJarosz-Wilkołazka, AnnaTirado, Diego F.Jarzebski, MaciejTirillini, BrunoJasenka, Gajdoš KljusurićTiritan, Maria ElizabethJasiński, MariuszTischler, DirkJasinski, RadomirTisovský, PavolJaśkowska, JolantaTitma, TiinaJastrzębska, AnetaTitov, Aleksei A.Jastrzebska, BeataTius, MarcJastrzębska-Więsek, MagdalenaTiwari, Arjun PrasadJasutiene, InaTiwari, Rakesh K.Jaszcz, KatarzynaTjahyono, Daryono HadiJatkowska, NataliaTkac, JanJaudzems, KristapsTkachenko, KirillJaumot, JoaquimTkacz, KarolinaJaworska, MariaTlak Gajger, IvanaJayanthi, SankarTo, Dao CuongJayaraman, SelvarajTobaldi, David MariaJayawardena, DulariTobiszewski, MarekJayawardena, Thilina U.Tobrman, TomášJazvinšćak Jembrek, MajaTocmo, RestitutoJaźwiński, JarosławTodorov, RossenJedrejek, DariuszTodorov, SvetoslavJedrzejewska, BeataTodorović, Vanja M.Jeleń, MałgorzataToepp, Angela J.Jena, Himanshu SekharTofana, MariaJenkins, IanToffano, ZenoJensen, FrankTokarz, BarbaraJeong, DaewonTokarz, PaulinaJeong, HanseobTokarz-Sobieraj, RenataJeong, Keun-YeongTolborg, KasperJeong, Seong HoonTolgyessy, PeterJeremic, JovanaTolkou, AthanasiaJerez, NancyTolochko, OlegJerković, IgorToma, VladJeromel, AnaTomalia, DonaldJeruc, JeraTomás-Barberán, Francisco A.Jerzsele, ÁkosTomasevic, IgorJesionowski, TeofilTomašič, TihomirJethava, Krupal P.Tomaz, IvanaJeucken, AikeTomažin, UrškaJevrić, LidijaTomi, FélixJezierska, AnetaTomonaga, TaisukeJha, Saurav KumarTonelli, MicheleJheng, Li-ChengTonello, FiorellaJi, GuozhaoTong, LinyueJi, ShaofeiTong, TaoJia, ShangTong, Woei YennJia, ZheTong, YexiangJia, ZixianTopete Camacho, AntonioJian, ZhongbaoTopin, JeremieJiang, CanTopunov, Alexey F.Jiang, GuangdeTornabene, FrancescoJiang, XuntianTorneiro, MercedesJiang, ZhenTornesello, Anna LuciaJiang, ZiwenToro, AyelénJianu, CalinTorović, LjiljaJiao, YanTorras, JoanJicsinszky, LászlóTorre, Maria LuisaJiménez Arellanes, AdelinaTorregiani, ElisabettaJiménez-Monreal, Antonia M.Torrent-Sucarrat, MiquelJiménez-Tenorio, ManuelTorres, AscensionJin, CongruiTorres, MarielaJin, Guo-XinTorri, GiangiacomoJin, Hyun-SeokTorrisi, SebastianoJin, MinTorroba, TomasJing, HuiTortora, PaoloJing, YuTósaki, ÁrpádJing, ZhifengToseland, Christopher P.Jiri, VrbaToshchevikov, Vladimir P.Jitǎreanu, AlexandraTošović, JelenaJo, KyubongTosti, TomislavJoão Ferreira, MariaTot, AleksandarJodłowski, GrzegorzTóth, GergőJohannes, Hans-HermannToth, RachelJohannissen, Linus O.Toti, KiranJohansen, BeritTotsingan, FilbertJohansson, AndersTouloupakis, EleftheriosJohnsen, Kasper BendixTouraud, DidierJohnson, EricTozar, TatianaJohnston, Linda J.Trader, DarciJohnston, Martin R.Tramonti, AngelaJoksović, LjubinkaTran, Anh VyJoly, NicolasTran, Quang AnhJones, MarjorieTrandafirescu, Cristina-MariaJones, RichardTrapani, MariachiaraJong, Chian JuTravagli, ValterJonnalagadda, SreekanthTravaglini-Allocatelli, CarloJönsson, FranziskaTrcek, JanjaJoo, TaihaTrdan, StanislavJorge Guiomar, AntónioTremlová, BohuslavaJorge, J. C. LuisTrencsényi, GyörgyJorrin-Novo, Jesus V.Trentini, AlessandroJosé, Vidal-GancedoTreps, LucasJoubert, AnnieTres, Marcus ViniciusJourné, FabriceTrevisan, AndreaJovancicevic, BranimirTribst, João Paulo MandesJozanović, MarijaTricoire, LudovicJu, Wei-JhongTrif, MonicaJuan García, CristinaTrifan, AdrianaJuárez, EsmeraldaTrifonova, Oxana P.Judeh, ZaherTrigo-Rodríguez, Josep M.Judek, JarosławTrimigno, AlessiaJudge, Peter J.Trinh, ThuatJudzentiene, AstaTriola, GemmaJukič, MarkoTripathi, Jaindra NathJulien, Christian M.Tripathi, MadhulikaJullian, NathalieTripathi, NishantJumbo-Lucioni, PatriciaTrivedi, VivekJuncan, Anca MariaTroczka, BartlomiejJung, ChristianeTroncoso-Ponce, AdrianJung, Gang SeobTrong Tuan, NguyenJung, HyunsookTrotta, TeresaJung, JaehanTrubetskaya, AnnaJung, Ji HoonTrujillo-Cayado, Luis AlfonsoJung, Julie Cathy Antoinette OdileTrushkov, Igor V.Jung, Sung KeunTruzzolillo, DomenicoJungnickel, ChristianTryznowski, MariuszJunk, PeterTrzaskowski, BartoszJunka, AdamTrzcińska, MonikaJurado, Amalia S.Trzebicka, BarbaraJurasek, MichalTsafantakis, NikolaosJurcek, OndrejTsai, FumingJurczak, JanuszTsai, Kun-LinJurić, SlavenTsai, Shu-YaoJusticia, JoséTsai, Wan-YuJuszczak, LesławTsai, YuhsuanJůza, JosefTsai, Yung-HsiangKabelác, MartinTsakovska, IvankaKacjan Maršič, NinaTsaltas, DimitrisKącka-Zych, AgnieszkaTsatsanis, ChristosKaczmar, JacekTseng, Chih-HuaKaczmarek, Małgorzata T.Tseng, Ping-HuiKaczmarek-Kędziera, AnnaTsiafoulis, Constantinos G.Kaczor, Agnieszka A.Tsikritzis, DimitrisKadam, AvinashTsim, KarlKadeethum, TeeratornTsimidou, MariaKadela-Tomanek, MonikaTsingotjidou, AnastasiaKadikova, RitaTsipa, ArgyroKafarski, PawełTsirelson, Vladimir G.Kahr, BartTsironi, FannyKaim, WolfgangTsivileva, OlgaKaiser, AnetteTskhovrebov, Alexander G.Kaizer, JakubTsubaki, ShuntaroKajimoto, ShinjiTsuchida, KunihiroKajiyama, SatoshiTsuda, HidetoshiKakkassery, VinodhTsuji, HidetoKakoschke, NaomiTsukamoto, TakashiKakoti, AnkanaTsukuda, ToshiakiKakuchi, RyoheiTsuneda, TakaoKálai, TamasTsunoda, MakotoKałas, WojciechTsutsumi, HiroshiKalayda, GannaTubaro, CristinaKalemba-Drożdż, MałgorzataTucci, PaoloKalidindi, Teja MuralidharTudela, JoseKalíková, KvětaTullius Scotti, MarcusKalinin, VladimirTůma, PetrKalinova, JanaTůma, ZdeněkKalinowska Lis, UrszulaTumbas Šaponjac, VesnaKalinowski, SlawomirTünde, JurcaKalish, AndreyTundis, RosaKalisz, StanisławTuñón, María J.Kaliyan, NalladuraiTurco, AntonioKaljurand, MihkelTurek, ArturKällbom, SusannaTuriák, LillaKallithraka, StamatinaTurk, TomKallitsis, Joannis K.Turkiewicz, Igor PiotrKalogiouri, NatasaTurło, JadwigaKalogirou, Andreas S.Turner, DavidKalsi, MeghaTurrini, FedericaKaltsa, OlgaTurutin, AndreiKalušević, AnaTuttolomondo, TeresaKamal, Muhammad ShahzadTuzimski, TomaszKamali, HosseinTvrdá, EvaKamatou, Guy P.Tyagi, ChetnaKamble, Shyam H.Tykarska, EwaKamdem, Jean PaulTyler, RobertKamecka, AnnaTzanavaras, Paraskevas D.Kamedulski, PiotrTzani, AndromachiKamel, KarolTzanova, MilenaKameta, NaohiroTzeli, DemeterKamgang Nzekoue, FranksTzen, Jason T. C.Kamiloglu, SenemTziritis, EvangelosKaminska, BozenaTziveleka, Leto-AikateriniKaminski, KamilUbeda, CristinaKaminski, Rafal M.Ubukata, TakashiKamzolova, SvetlanaUchida, LéoKan, Chi-WaiUchiyama, TaketoKan, TaoUdal’tsov, AlexanderKanamarlapudi, VenkateswarluUddin, Md JamalKanamori, TakaoUddin, Mohammed JashimKanaujiya, JitendraUdrescu, LucretiaKanazawa, KenUdvardy, AntalKancharla, PapireddyUeda, TaroKandikattu, HemanthUekusa, HidehiroKandil, SaharUemura, KoichiKang, BongchulUenishi, HirohideKang, ChulhunUeno, MikinoriKang, Hee EunUetz, PeterKang, JianmingUgo, PaoloKang, JinheUivarosi, ValentinaKang, Jun-GillUkalska-Jaruga, AleksandraKang, Sung GuUlasov, IlyaKaňková, KateřinaUllah, Mohammad FahadKanna Reddy, Hariprasada R.Ullah, RiazKannan, KurunthachalamUllrich, CarstenKano, NaokiUl’yanovskiy, NikolayKanoun, Mohammed BenaliUmdale, SurajKantminienė, KristinaUmerska, AnitaKao, Richard Yi TsunUmoren, Saviour A.Kao, Shao-HsuanUnković, NikolaKapelko-Żeberska, MałgorzataUrakawa, OsamuKar, Rajiv KumarUrayama, KenjiKaradžić Banjac, MilicaUrbanc, BrigitaKaraffova, VieraUrbańczyk-Lipkowska, ZofiaKaralis, Vangelis D.Urbanek, PavelKaraman, RafikUri, AskoKaramanov, AlexanderUribe, Kepa B.Karczmarczyk, AgnieszkaUribe-Calderon, Jorge A.Karczmarzyk, ZbigniewUrmann, CorinnaKarelson, MatiUrraca, Javier L.Kargar, HadiUrriolabeitia, EstebanKarimov, DenisUrzi, ClaraKarioti, AnastasiaUsman, AnwarKarl, RougerUsmani, ZebaKarnati, Konda ReddyUspenskaya, Elena V.Karpiński, Tomasz M.Usuki, YoshinosukeKarrouchi, KhalidUyanik, MuhammetKartalović, BrankicaVaccaro, MariaKartini, KartiniVagelas, IoannisKartnaller, ViniciusVahcic, NadaKarunarathna, Samantha C.Vaiano, FabioKaruppan, Mohan Kumar MuthuVaiano, VincenzoKaruppasamy, K.Vaidyanathan, GanesanKarushev, MikhailVakhin, AlexeyKarvaly, Gellért BalázsValandro, SilvanoKarwowski, BoleslawValarezo, EduardoKasapidou, EleniValduga, EuniceKasarda, RadovanValencia-Cantero, EduardoKashman, YoelValente, Anabela A.Kasicka, VaclavValente, Artur J. M.Kasiotis, KonstantinosValente, Ériton Egídio LisboaKasiotis, KostasValentová, KateřinaKaškonienė, VilmaValgimigli, LucaKašparovský, TomášValicherla, Guru RaghavendraKasprzak, ArturValik, LubomirKasprzak, KamilaVallée, YannickKassama, LaminVallejo Perez, Moises RobertoKataev, EvgenyValletta, AlessioKatalinić, MajaValoti, ErmannoKatharigatta, Venugopala NarayanaswamyVamanu, EmanuelKathiravan, SubbanVan Beusechem, VictorKati, FinzelVan Deenen, NicoleKato, KeisukeVan Den Meiracker, Anton H.Kato, KoichiVan Der Maelen, Juan F.Katsamakas, SotiriosVan Der Nest, Magriet A.Katsiotis, AndreasVan Dyke, MichaelKatsuyama, YoheiVan Hal, JaapKatz, EvgenyVan Hecke, KristofKauss, TinaVan Herk, AlexKawabata, KyuichiVan Mourik, TanjaKawahara, MasahiroVan Quan, NguyenKawakami, AkinoriVančik, HrvojKawakami, JunVande Velde, ChristopheKawakami, SatoshiVanderlei, Maria De FátimaKawamura, AkiraVandevoorde, CharlotKawamura, FumioVanella, LucaKawasaki, KiyonoriVanelle, PatriceKawasaki, RikuVanin, Fernanda MariaKawato, TakayukiVankayala, RavirajKawczak, PiotrVannam, RaghuKawecki, R.Vannini, AndreaKawiak, AnnaVanti, GiuliaKay, RichardVanura, KatrinaKayaki, YoshihitoVaraksin, MikhailKazachenko, AlexandrVarela-Ramirez, ArmandoKazakova, Oxana B.Varga, GáborKazemi-Lomedasht, FatemehVarga, ZoltanKazeto, YukinoriVargas-Baca, IgnacioKazokaite, JustinaVargas-Murga, LilianaKędziora, AnnaVargas-Rodriguez, EverardoKeglevich, GyörgyVargas-Sánchez, Rey DavidKeglevich, PéterVari, Camil-EugenKelava Ugarković, NikolinaVarma, Rajender S.Kele, PeterVarna, MarianaKellogg, GlenVarol, EsinKelly, HelenaVarone, AlessandraKennedy, David C.Varvaresou, AthanasiaKenry, K.Varvounis, GeorgeKepinska, MartaVasco Vidal, AldrinKeppert, MartinVashist, SandeepKerch, GarryVashurin, Arthur S.Kern, WolfgangVasic, VesnaKerr, IanVasicek, OndrejKerr, PhilipVasilatos, CharalamposKert, MatejaVasileva, LiliyaKertmen, AhmetVasilevskaya, ValentinaKesari, Kavindra KumarVasiliev, AleksandrKesavulu, C. R.Vasiliev, Mikhail M.Keshavarz, MeysamVasilyev, Aleksander V.Kęska, PaulinaVasko, PetraKeutgen, AnnaVaskoska, RozitaKeyel, Peter A.Vasos, PaulKeyzers, RobVasquez, MarlenKhachatryan, DerenikVassalini, IreneKhadka, Dhruba B.Vassilev, NikolayKhafizov, Kamil F.Vattikuti, Surya Veerendra PrabhakarKhairullina, VeronikaVayá Pérez, IgnacioKhalifa, ShadenVaz Junior, SilvioKhallouki, FaridVaz, Pedro D.Khan, AmjadVaze, Nachiket D.Khan, AnzarVazquez Espinosa, MercedesKhan, FahadVázquez-Flota, FelipeKhan, FazlurrahmanVázquez-Ovando, AlfredoKhan, Firdos AlamVázquez-Tato, M. PilarKhan, HaroonVaz-Velho, ManuelaKhan, Md. ShakhaoathVeclani, DanieleKhan, MerajuddinVédova, Carlos O. DellaKhan, Mohammad EhtishamVega-Holm, MargaritaKhan, MujeebVega-Rios, AlejandroKhan, NaimVegvari, AkosKhan, Riaz A.Veiga Junior, Valdir F.Khanal, AadityaVeiga, SilvioKhanal, Hari DattaVeiga, Thiago André MouraKhanh Le, Nguyen QuocVek, ViljemKhatri, VishalVelasco, DiegoKhiari, RamziVelasco, Maria ValériaKhlebnikov, Alexander F.Velázquez-Campoy, AdrianKhlestkin, VadimVelena, AstridaKhodade, VinayakVelepic, MarkoKhodorkovsky, VladimirVelgosova, OksanaKhoo, CheangVelkovic, DusanKhotimchenko, Maxim Y.Vella, Filomena MonicaKhouchaf, LahcenVeloso, AntonioKhrapak, SergeyVeloso, Cristiane MartinsKhrennikov, AndreiVeltri, LuciaKhubezhov, Soslan A.Vembris, AivarsKhurshid, ChrowVemulpad, Subramanyam R.Kia, RezaVenditti, MassimoKicel, AgnieszkaVenditto, VincentKiec-Kononowicz, KatarzynaVénien-Bryan, CatherineKiegiel, KatarzynaVenieraki, AnastasiaKijenska, EwaVenkatasubramanian, AnandramKikuchi, TakeshiVenuti, ElisabettaKikushima, KotaroVenuti, ValentinaKilbane, JohnVerbanac, DonatellaKiliś-Pstrusińska, KatarzynaVerdejo, BegoñaKim, Byoung SuhkVerdú-Andrés, JorgeKim, ChaekyunVergallo, AndreaKim, ChangsooVerho, OscarKim, ChealVerissimo, PaulaKim, Cheol-SuVerma, AnilKim, Hei-SungVerma, PragyaKim, Hong-DuckVerma, RajniKim, HoonVermathen, MartinaKim, Hyung MinVermeire, FlorenceKim, Hyun-OukVernardou, DimitraKim, HyunsungVernuccio, SergioKim, Jae KwangVeronesi, FedericoKim, JaehanVerri, TizianoKim, Jae-SeokVeselova, Svetlana V.Kim, Jin-ChulVetica, FabrizioKim, Jong-SangVia, BrainKim, Joo-HyungVianello, RobertKim, JuhyungVianna, María FlorenciaKim, JunheonVicaș, Laura GrațielaKim, Ki-YoungVicens, Quentin JeromeKim, Kyoung SubVicente, Filipa A.Kim, Kyung BoVicente, TaniaKim, KyunggonVidic, JasminaKim, Kyung-MinVidovic, BojanaKim, MinVidović, SenkaKim, Min-KyuViegas, CarlosKim, Min-SikVieira, Melissa Gurgel AdeodatoKim, Moon IlVieira, Nicole S. M.Kim, MyungwoongVieira, VanessaKim, NamdooViennet, ThibaultKim, Seok-HoVigneresse, Jean LouisKim, SeonghunVijayakumar, SekarKim, SeonilVijayan, MadhuvanthiKim, Seung-HyunVijgenboom, ErikKim, SeungjuVikulina, AnnaKim, ShinVil’, Vera A.Kim, Soo-ahVila, NeusKim, Tae HeeVilaivan, TirayutKim, Tae-HyunVilardi, GiorgioKim, TeayounVilela, AliceKim, Woo YoungVillarreal, Vicent Sixte SafontKim, Woo-HeeVillarreal-Gómez, Luis JesúsKim, YoosikVillarruel-López, AngélicaKim, Young JunVillegas, VictoriaKim, Young SikVillena, JuanKim, YoungmeeViña, DoloresKim, YoungminVincenzi, ColombinaKim, Young-SangVincenzi, SimoneKimbaris, AthanasiosVinci, GiulianaKimura, Ken-ichiVinet, RaúlKimura, YoshiharuVinodh, RajangamKinarivala, NiharVinokurov, SergeyKinder, DavidVinšová, JarmilaKing, Joan M.Virágh, MátéKinspergher, StefaniaVirjamo, VirpiKintz, PascalVirt, Ihor S.Kirchmaier, AnnVirta, PasiKirillov, AlexanderVisa, AureliaKirillova, MarinaVisentin, FabianoKirilov, PlamenVisioli, FrancescoKirsch, Gilbert H.Viškelis, PranasKirubakaran, PalaniVismara, ElenaKiryukhantsev-Korneev, PhilipVisnapuu, TriinuKiselev, Konstantin V.Visseaux, MarcKiselev, VitalyVistas, Claudia R.Kishore Sakharkar, MeenaVistoli, GiulioKiskin, MikhailVitturi, DarioKiss, Nóra ZsuzsaViuda-Martos, ManuelKiss, TivadarVivas, MarceloKitagawa, HaruakiViveros‐Ceballos, José LuisKitagawa, ToshikazuVizireanu, CameliaKitamura, MitsuruVizler, CsabaKitamura, YoshiakiVladescu, MarianKitano, YoshikazuVladić, JelenaKitaura, HidekiVladkova, TodorkaKitrytė, VaidaVlahopoulos, SpirosKittl, SonjaVlaicu, Ioana DorinaKitulwatte, Indira D. G.Vlam, MartKiuru, PaulaVliegenthart, HansKlang, VictoriaVlk, MartinKlapötke, Thomas M.Vo, Dat DuyKlar, AgnesVo, Duc-ThangKlaschka, UrsulaVoća, NevenKlein, AxelVochita, GabrielaKlementeva, SvetlanaVoelcker, GeorgKlemeš, Jiří JaromírVogel, ChristophKlenk, ChristophVögeli, BeatKlepel, OlafVoges, IngaKlika, Karel D.Vogt, NicolasKlimaszewska, EmiliaVoidarou, ChrysaKlochkov, Sergey G.Voit, BrigitteKlokov, DmitryVojnovic, SandraKlontzas, MichailVojtek, MartinKłopotowska, DagmaraVolcho, KonstantinKlotz, JamesVollbrecht, JoachimKlučáková, MartinaVolnova, Anna B.Kluczyk, AlicjaVon Dreele, RobertKlyachko, Natalia L.Vora, LalitkumarKmetič, IvanaVorauer-Uhl, KarolaKmiecik, DominikVorob’ev, Mikhail M.Kmita, AngelikaVostrikova, Kira E.Knecht, Kathryn T.Vougogiannopoulou, KonstantinaKnez, DamijanVouyiouka, StamatinaKnežević, AnamarijaVozzi, GiovanniKnippenberg, StefanVrabel, MilanKnoll, AlešVračko, MarjanKnuplez, EvaVranes, MilanKo, Kyung-SeokVrankova, StanislavaKo, Min-jungVrbacký, MarekKo, YounheeVu, Danh C.Koay, Yen ChinVukmirovic, SasaKobayashi, KenseiVukoje, MarinaKobayashi, YayoiVuts, JozsefKobiela, TomaszVysetti, BalaramKobrak, Mark N.Vyšniauskas, AurimasKoch, MatthiasWada, TohruKocić-Tanackov, SunčicaWaffo-Téguo, PierreKocisek, JaroslavWagemans, Anja MariaKöckerling, MartinWagler, JörgKoczorowski, TomaszWagner, AnikaKodani, Sean D.Wagner, LianeKodrin, IvanWagner, NathanielKoen, BoikoWagner, PawelKoetz, JoachimWaksmundzka-Hajnos, MonikaKoga-Ito, CristianeWałajtys-Rode, ElżbietaKogan, EugeneWalch, StephanKoh, Cho YeowWalczyński, KrzysztofKohli, KanchanWałecka-Zacharska, EwaKoikawa, MasayukiWali, Adil F.Koike, HarukiWali, QamarKoike, KenzoWalimbe, TanayaKoim-Puchowska, BeataWalker, Heather JaneKoivisto, JanneWalkiewicz, KatarzynaKoizumi, TakeakiWall Medrano, AbrahamKokan, ZoranWallace, M. ArielKokkinakis, EmmanouilWaluk, JacekKokkola, TarjaWan, ZhiliKokkonda, PraveenWang, Be-JenKokol, VanjaWang, BoKokoska, LadislavWang, ChaoKokotou, Maroula G.Wang, Chia-YihKolanowski, WojciechWang, Chih-ChiehKolar, MitjaWang, Chin-KunKolawole, Abimbola O.Wang, Chun-KaiKołczyk-Siedlecka, KarolinaWang, ConanKolesniková, LucieWang, DaweiKolesov, Boris A.Wang, DiKoleva, VioletaWang, DingKoliopoulos, George T.Wang, FazuoKolling, StefanWang, FuhuiKolocouris, AntoniosWang, GuankuiKolodiazhnyi, Oleg IvanovWang, GuanwuKołodyńska, DorotaWang, HengbinKolodziejczyk, JoannaWang, HuKołodziejczyk-Czepas, JoannaWang, HuiKolodziejski, Pawel AntoniWang, Hui-MinKolomeǐchuk, SergeyWang, JeffreyKomatsu, SatoshiWang, JiajieKomnenov, DraganaWang, JiangKomoike, YutaWang, JiaoKomolov, AlexeyWang, JingyiKomsiyska, LidiyaWang, JinyanKonchenko, Sergey N.Wang, LiqiangKończak, MagdalenaWang, LirongKondo, TakahiroWang, LonggangKonev, AlexanderWang, LuguangKong, XiangpengWang, MengjingKong, XiangruiWang, NanKong, XiaoqingWang, PengchengKongkiatpaiboon, SumetWang, PingyuanKönig, JörgWang, QinghuiKoniorczyk, PiotrWang, RenjieKonno, AyumuWang, Robert Y.-L.Konno, HiroyukiWang, ShaoboKonopka, AnnaWang, ShengnianKonopka, IwonaWang, ShunliKonrad, LutzWang, Steven Sheng-ShihKonstantinos, LitinasWang, TingKontek, BogdanWang, WeiKontogiannopoulos, KonstantinosWang, WenjiaKontogiorgis, ChristosWang, WenyuKontominas, MichaelWang, WenyuanKopach, OlgaWang, XiaoliangKopeć, AnetaWang, XiaomingKopitzky, RodionWang, XiaoyuKopjar, MirelaWang, YanKopke De Aguiar, Jair AdrianoWang, Yang-KaoKoppaka, AnjaneyuluWang, YanwenKopra, KariWang, Yeng-TsengKopustinskiene, Dalia M.Wang, YinKopyl, SvitlanaWang, YirenKopylova, Galina V.Wang, Yong-LeiKorbecki, JanWang, YueshengKorczeniewska, OlgaWang, Yun-MingKordulewska, NataliaWang, Yun-YanKordyum, ElizabethWang, ZheKorla, Praveen KumarWang, ZhenKorolkov, Ilya V.Wang, ZhenxinKoronkiewicz, StanisławaWang, ZijunKortaberria, GalderWang, ZiwenKos, BlaženkaWang, ZongjieKoschella, AndreasWang, ZunyaoKościelniak, PawełWant, Muzamil YaqubKöse, KazımWarren, J. DavidKoshino, HiroyukiWarren, Patrick BKosicka-Noworzyń, KatarzynaWasilewski, TomaszKosinsky, Robyn LauraWasylka, Justyna PłotkaKosma, Ioanna S.Watanabe, KazukiKosma, PaulWatanabe, YasuoKosmala, MonikaWaterlot, ChristopheKošmerl, TatjanaWaterman, RoryKost, BartłomiejWawrzyniak, JolantaKostakis, IoannisWazed, Md AbdulKostantinidis, TheocharisWdowin, MagdalenaKostas, IoannisWeber, MatthieuKostić, Aleksandar Ž.Weger, StefanKostić, MarinaWei, Da-HuaKostov, Konstantin GeorgievWei, GangKosuge, YasuhiroWei, HaoranKosyakov, Dmitry S.Wei, JunhongKőszegi, TamásWei, KajiaKoszelewski, DominikWei, LijunKot, AnnaWei, PingrongKótai, LászlóWei, WeiKotek, JanWeigand, WolfgangKothandaraman, JotheeswariWeinhold, FrankKothari, Vishal M.Welch, KevinKotkowiak, WeronikaWelker, MarkKotnik, PetraWemyss, AlanKotora, MartinWen, JianjunKotra, LakshmiWeng, ZhihuanKotynia, AleksandraWenthold, Paul G.Koufaki, MariaWenzel, BarbaraKoulman, AlbertWerner, EnriqueKouretas, DimitriosWesołowski, MarekKoutelidakis, Antonios E.Weßels, IngaKovač, TihomirWest, James D.Kovačević, AndjelkaWeston, ChrisKovacheva, DanielaWestwell, AndrewKovacs, Melinda HaydeeWhitehill, AndrewKoval, AlexeyWhitfield, Phil.Kovalchuk, VolodjaWhyte, ClaireKovalenko, AndriyWianowska, DorotaKovinich, NikWidelski, JarosławKowalczyk, MariuszWidemann, EmilieKowalczyk, TimWieczfińska, JoannaKowalczyk, TomaszWieczór, MiłoszKowalewska-Kudłaszyk, AnnaWieczorek, DariaKowalska, HannaWieczorek, EwaKowalski, RadosławWiedey, RaphaelKoyioni, MariaWiegand, ThomasKozak, JoannaWieland, D. C. FlorianKozaki, AkikoWiener, ReuvenKozaki, DaisukeWiglusz, KatarzynaKozakiewicz, AnnaWiklander, OscarKozakiewicz, JanuszWilber, Andrew C.Kozioł, Anna E.Wilkowska, AgnieszkaKozlevčar, BojanWilliams, FrederickKozlík, PetrWilliams, Noelle S.Kozlov, Sergey M.Willmore, William G.Kozlova, TaisiyaWilson, Alphus D.Kozłowska, JoannaWilson, BriceKozłowska, MariolaWilson, Erica GeorginaKoźmiński, WiktorWilson, JulieKozyra, MałgorzataWimalasinghe, RasangiKragović, MilanWimmer, ZdenekKragulj Isakovski, MarijanaWindshügel, BjörnKrajewska, BarbaraWinkler, AnjaKralj, SamoWińska, KatarzynaKrálovec, KarelWiraja, ChristianKranjc, KrištofWitczak, Carol A.Kratky, LukasWitczak, MariuszKratochvilova, IrenaWitczak, Zbigniew J.Kraus, TobiasWitkiewicz, ZygfrydKravets, NinaWitola, William HaroldKravets, VolodymyyrWitt, KatarzynaKrawczyk, TomaszWlaz, PiotrKrawiec, MariuszWłoch, MarcinKrcma, FrantisekWłodarczyk, BarbaraKreft, IvanWłodarczyk, MaciejKreitman, GalWłodarczyk, RenataKrichevtsov, BorisWłodarczyk-Stasiak, MarzenaKrieghoff-Henning, EvaWoeltje, MichaelKrieke, GunaWojaczyńska, ElżbietaKrikstolaityte, VidaWojaczyński, JacekKrishna, ShagunWójciak, AdamKrishna, Suresh Babu NaiduWójciak, MagdalenaKrishnakumar, DevadasWójcicka, GrażynaKrishnan, NatrajWojciechowska, AgnieszkaKrishnasamy, GopinathWojciechowski, KamilKrivoshapkina, Elena FedorovnaWójcik-Pszczoła, KatarzynaKrizman, MitjaWojdylo, AnetaKrokidis, MariosWojnicz, DorotaKról, EwelinaWojtunik-Kulesza, Karolina AnnaKról, MałgorzataWojtyczka, RobertKrólicka, AgnieszkaWolf, KatarinaKrompiec, StanisławWölfle, UteKronek, JurajWollanke, BettinaKronekova, ZuzanaWołowiec, StanisławKronenberger, ThalesWolska, JoannaKrstic, DanijelaWolski, KarolKrstić, MarkoWong, Chi-MingKručaitė, GintarėWong, ChungKruchinina, Natalia EvgenievnaWong, Clarence Tsun TingKruk, AndrzejWong, Fung-FuhKrulj, JelenaWong, Ka KanKrupa, KristenWong, Man-kinKrupa, RenataWong, Tak-WahKrupa-Kozak, UrszulaWorrall, StephenKruszewski, BartoszWöstemeyer, JohannesKruszka, DariuszWright, DenisKrystofiak, TomaszWright, JosephKryštofová, SvetlanaWrobel, KamilKrystyna, Skalicka-WoźniakWrobel, MariaKryuchkov, FedorWroniak, MałgorzataKrzemiński, Marek P.Wu, BingKu, Bon-CheolWu, Chang-JerKu, Kang-MoWu, ChongmingKubala, LukášWu, Chung-ChihKubáň, PetrWu, Chung-HsinKubatka, PeterWu, ChunshengKubica, PawełWu, ChunyiKubíček, VojtěchWu, Ding-taoKubíčková, AnnaWu, Hsyueh-LiangKubina, RobertWu, JiandongKubínová, RenataWu, JohnKubinyi, MiklósWu, KunweiKubissa, WojciechWu, LeiKubyshkin, VladimirWu, LihuaKucerakova, MonikaWu, Min-HuanKucerik, JiriWu, QinglinKuch, BertramWu, RuilianKucharska, KarolinaWu, Shu-PaoKucharski, JanWu, WeiKucinska, MalgorzataWu, XinKucuk, IsrafilWu, YanyouKucukkal, TugbaWustoni, ShofarulKucwaj-Brysz, KatarzynaWychowaniec, JacekKudaibergenov, SarkytWydro, UrszulaKuddannaya, ShreyasWylie, Benjamin J.Kudłak, BłażejWysokiński, RafałKudo, FumitakaWyszkowski, MirosławKudryavtsev, Denis S.Wzorek, AlicjaKuehne, IrinaXavier, Cristina P. R.Kühn, OliverXi, YumengKuhn, RamonaXia, GuanglinKuhn, StefanXiang, XiaoqiangKühne, Irina A.Xiao, ChunshengKuhnle, GunterXiao, DequanKujawska, MałgorzataXiao, ZhengtaoKukina, Tatyana P.Xie, ChaomingKukovec, Boris-MarkoXie, FangKukula-Koch, WirginiaXie, LishiKula, SławomirXie, Wei-dongKulakovskaya, Tatiana V.Xiong, ChuanyinKulbacka, JuulitaXiong, JiaKulda, VlastimilXochitl Lopez-Martinez, LeticiaKulikova, Natalya A.Xu, ChungangKulsing, ChadinXu, HongtaoKumada, KanakoXu, JiaxiKumar, AbhishekXu, JiminKumar, AmitXu, JingKumar, HirdeshXu, LuKumar, K. J. SenthilXu, MinghaoKumar, KeshavXu, NanKumar, ManishXu, RuoyuKumar, PawanXu, ShuKumar, PradeepXu, WeiKumar, PraveenXu, XinfangKumar, RajXu, Yan-MingKumar, RajeevXu, YongquanKumar, SanjayXuan, TongtongKumar, ShaileshXuan, Tran DangKumcuoglu, SeherXue, ChundongKumirska, JolantaXue, YongboKumpulainen, TatuXynias, Ioannis N.Kung, Chung-WeiYablonsky, Gregory S.Kung, Yu-RueiYabuki, YasushiKunsági-Máté, SándorYadav, DhananjayKuo, I-feng WilliamYadav, Dharmendra K.Kuo, Ping-ChungYadav, Hemraj MKuo, ShiaoweiYadav, Pramod KumarKuo, Shu-JuiYadav, ShrutiKuo, WatsonYajima, ShunsukeKuo, Yao-HaurYakovtseva, OlgaKupfer, StephanYakushev, VladimirKurata, HiroyukiYamada, MichioKurc, BeataYamada, TsuyoshiKurdziel, MagdalenaYamagishi, TakehiroKureha, TakumaYamaguchi, AyakoKurek-Górecka, Anna M.Yamaguchi, MakotoKurg, ReetYamakoshi, HiroyukiKurganov, BorisYamamoto, AkihikoKurka, OndrejYamamoto, TsuyoshiKuroda, ChiakiYamano, YoshiKuroda, MasashiYamauchi, SatoshiKuroda, TakayoshiYamazaki, ToshioKuropka, PiotrYan, HaiKurtán, TiborYan, HuiKuryłowicz, AlinaYan, Yong-BinKusakabe, KatsukiYanagi, HisaoKushibiki, ToshihiroYanaka, SaekoKuśtrowski, PiotrYanase, EmikoKusukawa, TakahiroYaneva, Zvezdelina L.Kusuma, Victor A.Yang, Chen-IKutasi, KingaYang, Chia-MinKutikhin, AntonYang, ChuanyuKuwabara, TakuyaYang, ChunzhangKuźma, ŁukaszYang, Ding-YahKuzmin, AlexeiYang, GuangdongKuznetsov, AlekseyYang, HongshunKuźniarska-Biernacka, IwonaYang, In-SangKvasnica, MiroslavYang, Jiann-JouKwak, Hyo WonYang, JingKwak, SeonyeongYang, JunKwaw-Mensah, DavidYang, Kai MinKwiatkowski, MirosławYang, LeiKwiecień, MałgorzataYang, LetaoKwofie, Samuel K.Yang, LijiangKwok, Hang Fai (Henry)Yang, MingKwon, Duck-HeeYang, Nae-CherngKwon, Ji EonYang, QiangKwon, SunohYang, Seung HwanKyriakopoulou, KonstantinaYang, Seung-JaeKyzas, GeorgeYang, WeiKyzioł, AgnieszkaYang, Wei-HsiungLa Clair, JamesYang, Yu-LiangLa Motta, ConcettinaYang, ZhaogangLa Russa, DanieleYang, ZheLabay, CedricYang, ZhujinLabbozzetta, ManuelaYanina, IrinaLabuckas, DianaYano, MasatoLabudda, MateuszYanshole, Lyudmila V.Lacaille-Dubois, Marie-alethYao, ChunsuoLachenmeier, Dirk W.Yao, Pei-LiLadero, MiguelYao, XiwenLado-Touriño, IsabelYaoita, YasunoriLaezza, AntonioYap, Pei LayLafrenie, Robert M.Yaraki, Mohammad TavakkoliLagarde, MichelYaromina, AlaLages Rodrigues, BernardoYasui, KyuichiLaghi, LucaYavari, MiladLagoa, RicardoYe, HangLagravère, Manuel O.Ye, HuilinLaguna, HumbertoYe, ShufengLai, Chien-ChenYe, YaozongLai, OlimpiaYe, ZhuolinLai, Ping-ShanYeh, Chun-LiangLai, Pin-KuangYeh, Shu-LanLaine, Roger A.Yeh, Ting-FengLaity, Peter R.Yemmireddy, Veerachandra K.Lakard, BorisYen, Clive H.Lakin, Matthew R.Yener, IsmailLakowski, TedYeo, SynLalas, StavrosYi, Dong KeeLam, Sio-HongYi, LongLamac, MartinYi, MyunggiŁamacz, AgataYi, TaoLamanna, JacopoYilmaz, OnurLamari, Fotini N.Yin, ShengLambreva, Maya DimovaYin, WenqingLambrinidis, GeorgeYin, ZhouyangLamichhane, PradeepYingkun, QiuLamponi, StefaniaYochelis, ShiraLamzin, VictorYoganathan, SabesanLan, HangzhenYogeswara, Ida Bagus AgungLan, WenjianYoneyama, KaoriLan, YuchengYoneyama, TatsuroLancaster, PhillipYong, Kar WeyLandi, GianlucaYoo, Hah YoungLandreh, MichaelYoo, Michelle Ji YeonLangella, EmmaYoon, SukeunLankhorst, PeterYoshida, MasahitoLante, AnnaYoshida, Nidia CristianeLányi, KatalinYoshie, NaokoŁapiński, AndrzejYoshihara, ToshitadaLaporta, VincenzoYoshimitsu, TakehikoLaprevote, OlivierYoshioka, NaokiLaptev, RomanYou, DiLara, Hernán E.Youssef, Fadia SalahLaricchiuta, AnnaritaYu, BianyunLarin, AlexanderYu, Chang-FengLarin, SergeyYu, DaquanLarionov, VladimirYu, FabiaoLarios, Alejandro PérezYu, Feng-YihLaRocque, JanYu, HaiLaronze-Cochard, MarieYu, HaoLasalvia, MariaYu, Hsu-ShengLasik-Kurdyś, MałgorzataYu, Jae-WoongLaskaris, NikolaosYu, JieLaskowska, EwaYu, LeixiaoLasonder, EdwinYu, QiLatajka, RafałYu, QiangLatos-Brozio, MalgorzataYu, QingsongLatouche, CamilleYu, RongminLatté, Klaus PeterYu, WenquanLau, Andy T. Y.Yu, XinzhuLau, Beng FyeYu, YanboLau, ChunghoYu, YangxinLauberts, MarisYuan, RuiLaudadio, EmilianoYuan, Wei-EnLaughton, Charles A.Yuan, YeLauro, GianluigiYuan, ZhengnanLavric, VasileYuba, EijiLaw, Henry Chun HinYue, YuanyuanLaw, RubyYulong, LiLawal, Ismaheel O.Yun, Cheol-WonLawrence, NicoleYurchenko, EkaterinaLawrence, WalterYushina, IrinaLazar, CatalinYusuf, KamranLazar, Simone T.Zabetakis, IoannisLazarevic-Pasti, TamaraZabierowski, PiotrLazim, RaudahZabka, MartinLazzarato, LorettaZaboronok, AlexanderLazzari, MassimoZacconi, FlaviaLe Ferrand, HortenseZacharis, Constantinos K.Le Gall, TonyZadražil, AlešLe Guellec, DominiqueZafar Iqbal, ShahzadLe Pogam, PierreZafeiraki, EffrosyniLe Questel, Jean-yvesZafrilla, PilarLe Roes-Hill, MarilizeZagorchev, LyubenLe, Ngoc-TramZagórska, AgnieszkaLeal, João PauloZagotto, GiuseppeLebar, MatthewZaguła, GrzegorzLebeau, JulianaZaharia, CarmenLebedeva, Olga E.Zaharia, CatalinLebedyeva, IrynaZahoor, Ameer FawadLebouvier, NicolasZaid, HilalLebreton, JacquesZairov, RustemLeclerc, EstelleZając, WojciechLeclercq, LoicZajda, JoannaLecomte, PhilippeZajíčková, ZuzanaLedesma, Ana EstelaZakharova, ElenaLedwon, PrzemyslawZakharova, LuciaLee, Byoung-SunZakłos-Szyda, MalgorzataLee, ChangguZakrzewski, JerzyLee, Chang-HeeZalevsky, ArturLee, Cheng-IŽalga, ArtūrasLee, Chia-HungZaltariov, Mirela-FernandaLee, Ching-KuoZalubovskis, RavisLee, Dae YoungZambito, YleniaLee, Dae-SungZambrano, Maria De La LuzLee, Dong-ShengZammit-Mangion, MarionLee, DongyunŻamojć, KrzysztofLee, Hak LaeZamparini, FaustoLee, Hwa-JinZamudio-Flores, Paul BarukLee, Hyuck JinZamudio-Rivera, Luis S.Lee, HyunZanardi, EmanuelaLee, HyunbokZanetti, StefaniaLee, I-TaZang, LiqingLee, Jae SungZangrando, EnnioLee, JaegeunZani, Bianca MariaLee, Jey-JauZani, LorenzoLee, Jong HwaZanuttini, RobertoLee, Jong KilZaprutko, LucjuszLee, Jong-DaeZara, SeverinoLee, JunseongZara, VincenzoLee, JunsooZarca, GabrielLee, Ki-TeakZarić, Snežana D.Lee, Kuan-HanZarkov, AleksejLee, Li-TingZarkovic, NevenLee, Sang-HanZarrelli, ArmandoLee, SanghyunZarrintaj, PayamLee, SejoonŻary-Sikorska, EwaLee, Seong SeoZatsepin, Anatoly F.Lee, Shyi-LongZavala-Rivera, PaulLee, SihyunZavodnik, IlyaLee, SujinZavoianu, RodicaLee, Sung HoonZawada, KatarzynaLee, Sung-BauZayed, MohammedLee, SunwooZdziennicka, AnnaLee, Tse-MinZechel, StefanLee, Tsung-HsienZedda, MarcosLee, VictorZedler, ŁukaszLee, Yeon-JuZeeb, BenjaminLee, YohanZekker, IvarLee, YonghyunZeković, ZoranLee, YoungminŻelaszczyk, DorotaLee, ZhenghongŻelaziński, TomaszLees, Alistair J.Żelechowska, KamilaLegrand, François-XavierZelenovskiy, Pavel S.Leherte, LaurenceZelepuga, ElenaLehtonen, AriZeljkovic, AleksandraLeitão, Gilda GuimarãesZeljkovic, CavarLeitao, Ruben ElvasZeljković, Sanja ĆavarLelli, MorenoZemła, JoannaLeluk, KarolZendehboudi, SohrabLemercier, GillesZendo, TakeshiLemli, BeataZeng, JuqinLenaic, CouedelZeng, MinxiangLenci, ElenaZeng, TaoLeneweit, GeroZerani, MassimoLeng, YuxinZeremski, TijanaLengyel, JozefZgoda, VictorLennartz, MichelleZgură, IrinaLennernas, HansZhan, ZhuchangLentini, GiovanniZhang, ChaoLentz, Michael R.Zhang, ChengLenucci, Marcello SalvatoreZhang, ChunpengLenzi, Marcelo KaminskiZhang, Daniel XinLeón, FranciscoZhang, DianbaoLeón, Ignacio E.Zhang, DongshiLeon, JuanZhang, ErshuaiLeonardi, Antonio AlessioZhang, FanLeong, Max K.Zhang, FenghuaLeoni, AlbertoZhang, GenweiLeonidas, Demetres D.Zhang, HaiyuanLeón-Rivera, IsmaelZhang, HanLeontopoulos, StefanosZhang, HongweiLepik, Kirill V.Zhang, JianbinLeporatti, StefanoZhang, JianfeiLépori, Cristian M. O.Zhang, JianyingLerno, Larry A.Zhang, JianyongLeskovac, Andreja R.Zhang, JiaweiLeśniewska, BarbaraZhang, JinLešnik, SamoZhang, JinweiLesyk, Roman B.Zhang, Jun-BoLeszczyńska-Sejda, KatarzynaZhang, KaiLetsiou, SophiaZhang, LaibaoLetsyo, EmmanuelZhang, LiangLeu, Ching-ChichZhang, LiangrenLeus, KarenZhang, MingyiLevanat, SonjaZhang, MingziLevandowski, Brian J.Zhang, QiangLevesque, DominiqueZhang, QichunLevi, MosheZhang, QuanqingLévi, YvesZhang, RuimengLevin, Oleg VladislavovichZhang, RunLevy, DanZhang, ShanjuLewandowska, KatarzynaZhang, ShengLewińska, AgnieszkaZhang, WeiLewis, RandyZhang, WenLeychenko, ElenaZhang, WenhanLeyuan, LiZhang, WenjunLeyva-Gómez, GerardoZhang, WenkaiLeyva-Jimenez, Francisco JavierZhang, WenxuanLhiaubet, Virginie LyriaZhang, XiaoningLi, anboZhang, XinxiangLi, BinZhang, YanLi, ChengleiZhang, YiLi, ChenshuangZhang, YinfengLi, FengZhang, Yi-QiLi, FenghuaZhang, YongLi, GangZhang, YueLi, GuangliZhang, YuemeiLi, Guang-nZhang, YuezhouLi, GuangzhaoZhang, YujuanLi, GuohuiZhang, YuminLi, HanxiangZhang, YunLi, Hou-JinZhang, YuningLi, HuZhang, ZheLi, HuiZhang, ZhenLi, JianmeiZhang, ZhenganLi, JiapengZhang, ZhidongLi, JieZhang, ZhongzhengLi, JingZhao, DingLi, Jun YingZhao, FengyuLi, KaiZhao, HuiLi, LeiZhao, NingLi, NingZhao, PengyiLi, PenghaoZhao, Qin-ShiLi, Po-HsienZhao, WenjieLi, QunZhao, XiLi, ShimingZhao, XuebingLi, ShizhaoZhao, YanchuanLi, TianhuZhao, YaoLi, WeiminZhao, YongfengLi, XiaoZhao, YuxiaLi, XiaoweiZhao, ZhiminLi, YangZhao, ZongshanLi, YanweiZheng, AnminLi, YaozongZheng, Cai-MeiLi, Yi-PeiZheng, ErjinLi, YiwenZheng, FangLi, YuZheng, GuangchaoLi, YuxinZheng, HongLi, ZheZheng, HuandaLiagre, BertrandZheng, JieLiang, Chi-TeZheng, LibingLiang, JianwenZheng, ShilongLiang, Shun-XingZheng, SixunLiao, ChenghengZheng, UiLiao, Chien-SenZheng, ZhenjiaLiao, Hsiang-RueiZherikova, Kseniya V.Liao, Hsiao-WeiZhi, KainingLiao, Jyh-feiZhiponova, MiroslavaLiao, ZhihuaZhizhin, Konstantin YuLiaras, KonstantinosZhong, ChenhanLiaudanskas, MindaugasZhong, FangruiLiaw, Chia-ChingZhong, LixiangLicea, AlexeiZhong, Yu-WuLidon, FernandoZhou, AiminLiebman, JoelZhou, DongfangLieder, BarbaraZhou, GuangfengLigabue-Braun, RodrigoZhou, HongyiLigor, MagdalenaZhou, QihuiLigor, TomaszZhou, QingtongLigrone, RobertoZhu, ChangfuLikhatskaya, GalinaZhu, HuiLikozar, BlažZhu, XiaominLiliana, Mititelu-TartauZhu, XuejunLim, JiseokZhu, YuyangLim, Jun HeokZhu, ZhiwenLim, Kwang SukZhukov, IgorLim, SangbinZhuo, YueLima Ribeiro, MarceloZhuravleva, Olesya I.Lima, BeatrizZic, MarkLima, DênisŽídek, LukášLima, ElisabeteZięba, AndrzejLima, SofiaZielecka, MariaLimami, YounessZielińska, SylwiaLin, Chen-SiZielińska-Błajet, MariolaLin, Cherng-YuanZiembowicz, SabinaLin, Chia-HerZiemkowska, WandaLin, Chi-ChenZieniuk, BartłomiejLin, Chih-ChienZierkiewicz, WiktorLin, Chun-PingZiko, LailaLin, FengŽilić, DijanaLin, HaishuZille, AndreaLin, HanZimmerman, WilliamLin, HoŽinić, BiserkaLin, Hong-Ting VictorZinovi, DashevskyLin, Hsing-ChunZipori, IsaacLin, Hui-JuZippin, Jonathan H.Lin, Hung-YinZivanovic, JasminaLin, Hung-YuZiyatdinova, GuzelLin, Jer-AnZizzo, Maria GraziaLin, Long-LiuZjalić, SlavenLin, Mei-HsiangZlotin, Sergey G.Lin, RuweiZmudzki, PawelLin, ShaoyangZnamirowska, AgataLin, Shin-PingZochowska-Kujawska, JoannaLin, Shuo ParkZoidis, EvangelosLin, Tzu-EnŻołek, TeresaLin, WangŻołnierczyk, AnnaLin, Wei-ChaoZoratti, MarioLin, Wei-YiZubair, MuhammadLin, Yueh-ChienZubarev, RomanLin, Yu-LingZubrienė, AstaLin, ZhiweiZubrik, AntonLinciano, PasqualeŻubrowska-Sudoł, MonikaLindequist, UlrikeZucca, AntonioLinder, BenediktZuffo, MichelaLindsey, Jonathan S.Zukiewicz-Sobczak, WiolettaLindstrom, Jon MartinZúñiga, LeandroLinhari Rodrigues Pietro, Rosemeire CristinaZuo, HaoLinko, VeikkoZuverza-Mena, NubiaLipparini, FilippoZvorykin, VladimirLiritzis, IoannisZwergel, ClemensLisa, GabrielaZych, DawidLiška, AnitaZych, MariaListos, JoannaZyśk, MarlenaLiszkowska, JoannaŻyżelewicz, DorotaLiţescu, Simona Carmen



